# Unraveling Evolution in the Homoploid Complex of *Baccharis* L. in Chile

**DOI:** 10.1002/ece3.72249

**Published:** 2025-10-07

**Authors:** Fabian Schneider, Olga Zafra‐Delgado, Tobias G. Köllner, Frank Hellwig

**Affiliations:** ^1^ Systematic Botany With Herbarium Haussknecht and Botanical Garden, Faculty of Biological Sciences Friedrich‐Schiller‐Universität Jena Germany; ^2^ Department of Biochemistry Max Planck Institute for Chemical Ecology Jena Germany

**Keywords:** *Baccharis × intermedia*, *Baccharis linearis*, *Baccharis macraei*, GBS, homoploid hybrid, hybrid swarm, population genetics

## Abstract

*Baccharis × intermedia* (Asteraceae), found in central Chile, is a naturally occurring hybrid derived from the parent species *B. macraei* and *B. linearis.* It represents an extraordinary example of admixture with additive plant chemistry; however, the genetic structure of the hybrid complex and its evolution are still unclear. Intensive field sampling and Genotyping‐by‐Sequencing (GBS) were used to clarify the structure of the *B. × intermedia* hybrid complex. In addition, *B. vernalis*, another species that resembles the morphology of *B. macraei*, was subjected to analysis to ascertain its role in the hybridization process. A total of 3724 SNPs and 378 individuals were analyzed using clustering, PCA, Treemix, Patterson's *D*‐ and *f*‐statistics. Furthermore, other genetic indicators, such as levels of heterozygosity, Tajima's *D*, and nucleotide diversity (*π*) also provided further insight into the hybrid complex. Our results show that *B*. *× intermedia* consists mainly of F_1_‐hybrids with 18% backcrossing to both parental species. *B. vernalis* was not involved in recent hybridization with *B. macraei* and *B. linearis.* Additionally, a recent introgression into the Quintay population of *B. macraei* from *B. linearis* was detected during the analysis. There is no indication of hybrid speciation. Altogether, our extensive field sampling combined with genetic analyses has provided deeper insights into the genetic structure and evolution of the *B*. *× intermedia* hybrid complex in Chile.

## Graphical abstract

Genotyping‐by‐Sequencing (GBS) was used to characterize hybrids originating from the species *B. macraei* and *B. linearis* and identifying the substructure of the hybrid complex.

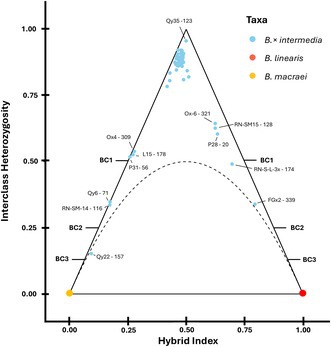

## 1 Introduction

Hybridization among higher plants is not a rare phenomenon. Mallet (2005) estimates, referring to a survey by Stace (1975) for the Flora of the British Isles, that a quarter of all species in higher plants are involved in hybridization. In another publication, Stace (1986) reports the number of 77,000 natural hybrids in vascular plants or 308 hybrid combinations in 1000 plant species. On the other hand, Yakimowski and Rieseberg (2014) estimate a much lower frequency of hybridization (~10%).

While there are no doubts about the relatively high frequency of hybrids in (higher) plants, its relevance for plant evolution is still under debate (Yakimowski and Rieseberg 2014). While it may have positive effects on diversity by hybrid speciation or by widening the space for recombining alleles through uniting allele pools of two species with divergent evolutionary history, some authors point to the effects of hybridization causing diversity loss (by extinction of rare species or the merger of lineages that had been distinctive before; for discussion, see Todesco et al. 2016).

Hybridization can be seen as mere accidents in the usual reproduction of outcrossing plants or considered a major factor in plant evolution and especially plant speciation (Stebbins 1959; Abbott 1992; Arnold 1997; Wissemann 2007; Soltis and Soltis 2009).

The evolutionary perspective on hybridization focuses on the question of whether, as a consequence, new stable lineages become established. The resulting lineages have to be protected against segregation and resorption by one parental lineage. The first biologist addressing this issue explicitly was Kerner von Marilaun (1871) when he asks: “Können aus Bastarten Arten werden?” (“Can hybrids become new species?”). Grant (1976) distinguishes seven ways to accomplish this, but only three of them involve sexual reproduction: amphiploidy, speciation by recombination, and segregation of a novel lineage isolated by external (ecological) barriers. Only the latter alternative allows for hybrid swarm production and introgression among the parental lineages.

Often but not always, hybridization is followed by genome doubling. The resulting allopolyploid hybrids may exhibit some advantages over their diploid parents, phenotypically apparent as heterosis, due to gene dosage effects, or as changes in the reproductive system (Osabe et al. 2012; Qiu et al. 2020 for review).

In some cases, however, hybridization is not followed by polyploidization. If several species are involved in hybridization, a homogamic complex may be formed, called a syngameon by Grant (1976). Grant states that such complexes may be able to quickly react to environmental change, rapidly filling novel ecological niches with suitable new lineages. Groups of species showing homoploid reticulation have received attention in the past, especially oaks (Bacilieri et al. 1996; Manos et al. 1999; Dodd and Afzal‐Rafii 2004; Hauser et al. 2017), willows (Kerner von Marilaun 1891; Grant 1976; Hardig et al. 2000; Fogelqvist et al. 2015; Marinček et al. 2023), or in *Ceanothus* (Rhamnaceae), another example of homoploid reticulation mentioned by Grant (Nobs 1963; Burge et al. 2013).

During the revision of the genus *Baccharis* L. (Asteraceae) in Chile (Hellwig 1990) morphological as well as ecological observations suggested the existence of a homoploid hybrid complex in this genus, comprising all species south of the Atacama Desert southward to Patagonia. Almost all species are endemic to Chile, with only few of them crossing the Andes in their southern part. Abundance of intermediate forms bridging morphological gaps between species further led to the hypothesis, that external factors maintained the identity of species, while no evidence could be found supporting hybrid speciation by recombination. Thus, the author interpreted the hybrid complex of Chilean *Baccharis* as an example for a syngameon sensu Grant (1976).

Encouraged by the improvement of molecular analytical techniques and increasing genomic information about the species involved in putative reticulation, *Baccharis* in Chile was revisited. We used GBS to analyze a small group of species within *Baccharis* L. to gain insight into the frequency and intensity of hybridization and the process of hybridization itself and discuss possible effects on the evolution of the species.

The genus *Baccharis* L. (Asteraceae, Astereae, Baccharidinae) is a genus native to the majority of Chile. However, it is absent in a region between 18° and 28°30′ (S. latitude) that is characterized by an almost vegetation‐free desert and the presence of the “loma” vegetation on the coastal hills. Furthermore, *Baccharis* does not occur in the “travesía” (a dwarf shrub heath) between Copiapó and Vallenar (Hellwig 1990). Hellwig (1990) identified 16 species of the subgenus *Baccharis* in Chile. Additionally, 21 hybrids of the aforementioned species have been described in the same geographical area based on morphological characteristics. In general, the genus itself is dioecious, and hybridization occurs when two or more species coexist in sympatry and overlap in flowering time (Hellwig 1990). All *Baccharis* hybrids and their parental species described so far by chromosome number are diploid (Hellwig 1990). Based on available morphological and genetic data, *Baccharis* in Chile is thought to form a complex hybrid swarm (see Schumer et al. 2014 for concept; Schneider and Hellwig 2024).

Several studies have been conducted on the most prevalent *Baccharis* hybrid complex in Chile, comprising the parents *B. macraei* Hook. & Arn. and *B. linearis* (Ruiz & Pav.) Pers., and the hybrid *B. × intermedia* DC. *Baccharis linearis* is found in central Chile, although it is absent from the coast in most areas. In contrast, *B. macraei* is restricted to the sandy dunes at the seashore. *Baccharis × intermedia* is only found in locations where both species are sympatric. The flowering periods of *B. linearis* and *B. macraei* overlap in February and March (Hellwig 1990). In gravelly sites in close proximity to the coastline, *B. macraei* is supplanted by *B. vernalis* F.H. Hellw., which exhibits morphological similarities to *B. macraei*, with the exception of its inflorescence and flowering period (Hellwig 1990). Schneider and Hellwig (2024) detected one individual that may represent a hybrid between *B. vernalis* and *B. macraei*. Yet this type of hybrid seems rather rare (Hellwig 1990; Schneider and Hellwig 2024).

Hellwig (1990) identified *Baccharis × intermedia* as a hybrid between *B. macraei* and *B. linearis* , based on morphological traits. In a study by Faini et al. (1991), the three taxa were also investigated morphologically. The hybrid was observed to display an intermediate morphology with respect to leaf shape, capitulum arrangement, and branch angles. Additionally, Faini et al. (1991) conducted a chemical analysis of the samples, focusing on the identification and quantification of terpenes and flavonoids. *Baccharis × intermedia* showed additive inheritance from both parental species. Furthermore, the authors concluded that *B*. *× intermedia* consists of a hybrid swarm. Based on the preliminary chemical work of Faini et al. (1991) it was shown by Zafra‐Delgado et al. (2025) that the chemistry of *B. × intermedia* combines compounds of the two parental species. Similar results were observed in studies on the hybridization of other plants (Orians 2000; Cheng et al. 2011). While the primary metabolism is highly conserved, differences between the parental species and the hybrid were mainly observed in secondary metabolites.

The results of a recent genetic study indeed have demonstrated that *Baccharis × intermedia* is a hybrid between *B. macraei* and *B. linearis* (Schneider and Hellwig 2024). Screening the distribution range of *B. macraei* for genetic structure revealed the potential differentiation of *B. macraei* into a southern and a northern group of genotypes (Schneider and Hellwig 2024). This finding was further investigated by Zafra‐Delgado et al. (2025). *Baccharis linearis*, however, did not demonstrate such differentiation. Additionally, it remains uncertain whether *B. vernalis* is also divided into north–south genotypes similar to *B. macraei*. *Baccharis vernalis* species exhibits remarkable morphological variability along a north–south gradient. This study focuses on analyzing the genetic structure of each species at the population level using a broad field sampling, a methodology that has not been carried out in previous studies with *Baccharis*.

Furthermore, it remains uncertain whether the observed genetic structure of *Baccharis macraei* is a consequence of genetic isolation by distance or if some populations were potentially founded or altered by human activities such as unintentional transport or land use change. The precise timing of the formation of the contact zones between the parental species also remains uncertain. *Baccharis linearis* has been observed along larger rivers, which could have served as migration routes (Hellwig 1990). In this context, contact zones with *B. macraei* may have been formed a long time ago, whereas at other localities, secondary contact may be much more recent, potentially due to anthropogenic alteration of primary vegetation.

The hybrid nature of *Baccharis × intermedia* having been validated, yet it remains unclear whether hybrid speciation could be observed. Schneider and Hellwig (2024) reported only backcrosses in the direction of *B. macraei*. This raises the question of whether this was a sampling error or whether one parent species is explicitly favored in backcrosses. If backcrosses with *B. macraei* were predominant, we would expect that *B. × intermedia* would be more similar to *B. macraei* than to *B. linearis* . Furthermore, the extent to which hybridization has influenced the genetic composition of the parental species remains uncertain. It is unclear whether introgression occurred into one or both parental species.

This study aims to characterize the hybrid swarm genetically and addresses the following questions in depth: (1) Is the genetic structure of each taxon best explained by isolation by distance or by other effects, such as human influence? (2) Are all hybrids F_1_‐hybrids, or can F_*n*_‐hybrids be observed? (3) Are there any indications that introgression may have occurred into the parental species? (4) Does hybrid speciation occur in the taxa under study?

## 2 Materials and Methods

### 2.1 Sample Collection

In March and September 2022, in total 231 plant tissue samples and herbarium vouchers from *Baccharis linearis*, *B. macraei*, and their corresponding hybrid *B. × intermedia* were collected in central Chile. In addition, 147 samples of *B. vernalis* were collected, mainly in September 2022. Most of the samples were collected in their flowering state, to identify the sex of the dioecious species. The leaf material was desiccated and stored in silica gel. The voucher specimens derived from these samples are housed in the Herbarium Haussknecht (JE). The precise geographical coordinates of all sampling sites were recorded using GPS. Where feasible, population size was estimated in the field, along with the relative abundance of males and females within a given population. Additionally, the vegetation at each site was characterized, and the abiotic constraints (including soil properties, exposure, and inclination) were documented.

### 2.2 General Sampling Strategy

**FIGURE 1:**
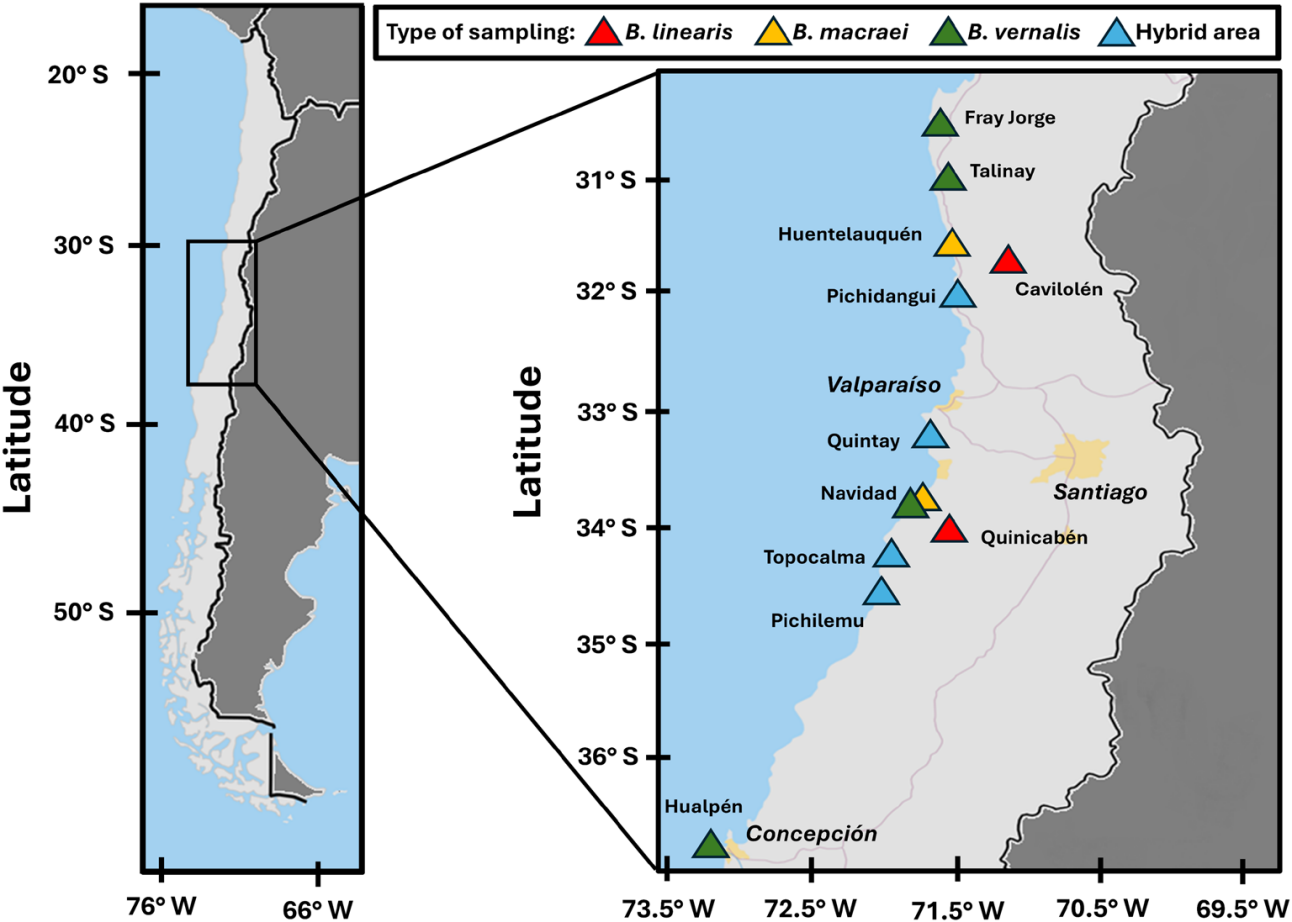
Sampling area of *Baccharis* hybrid swarms and reference populations in central Chile. Map showing the distribution of the different field locations, distributed across the coastal areas of Chile. Red triangles: *Baccharis linearis* reference populations; yellow triangles: *B. macraei* reference populations; green triangles: *B. vernalis* reference populations; blue triangles: Hybridization zones (transects).

To ascertain the extent of hybridization between the two parental species, *Baccharis macraei* and *B. linearis* , population samples were gathered along transects that encompassed both the parent species (*B. macraei* and *B. linearis* ) and the hybrid species (*B. × intermedia*). The populations sampled spanned the range from the coast (*B. macraei* – habitat) to inland ( *B. linearis* – habitat). Given the gene flow observed in the hybrid zone (see Hellwig 1990; Schneider and Hellwig 2024), the sampling strategy incorporated reference populations of the parental species situated at a considerable distance. These reference locations are presumed to represent the “pure” parental species, exhibiting no evidence of hybridization. As the parental species show internal variation in morphological characteristics (Hellwig 1990) and given that hybrid formation has been observed in disparate isolated localities, a sampling strategy was implemented across multiple sites encompassing the entire distributional range of the hybrid zone in Chile (Hellwig 1990). This area encompasses the entire distribution range of all taxa, with the exception of *B. linearis* . The selection includes localities where larger rivers may have served as migration routes for *B. linearis* (Pichidangui, Navidad). Here, contact zones with *B. macraei* may have been formed a long time ago, while at the other localities, secondary contact may be much more recent. Before starting the collection, the sites were visited, and the populations were assessed to determine whether they represented a suitable transect or reference population. See Figure 1 for the distribution of the sample areas.

A total of 15 individuals were sampled from reference populations, while 50 individuals (of *Baccharis macraei*, *B. linearis* , and *B. × intermedia*) were sampled from transect populations in autumn 2022. Due to the uncertain role of *B. vernalis* in the formation of the hybrid swarm of *B. × intermedia* and the morphological similarity of *B. vernalis* and *B. macraei*, this species was also included in this study. Reference populations were sampled for *B. vernalis* , and the same hybrid transects as mentioned before were revisited in spring 2022, and *B. vernalis* was sampled in a flowering state. For a general overview of samples per location, see Table 1. For a detailed description of the locations, see Appendix S1.

**TABLE 1:** Sampling design of transects and reference populations in the hybrid complex: Number of individuals selected from *Baccharis linearis* (Lin), *B. × intermedia* (Int), *B. macraei* (Mac), and *B. vernalis* (Ver).

Population	Lin	Int	Mac	Ver
Reference				
Fray Jorge	—	—	—	14
Talinay	—	—	—	21
Huentelauquén	—	—	—	15
Cavilolén	15	—	—	—
Navidad		2	16	22
Quinicabén	15	—	—	—
Hualpén	—	—	—	15
Transect				
Pichidangui	9	12	32	20
Quintay	16	19	15	—
Topocalma	14	20	16	25
Pichilemu	8	12	10	15

### 2.3 DNA Extraction and Sequencing

The tissue samples were transported from Chile in a desiccated state using silica gel. Extraction, sequencing, and Genotyping–by–Sequencing (GBS) were conducted by LGC Genomics GmbH (Berlin, Germany, https://www.lgcgroup.com/). DNA was isolated using the sbeadexTM mini plant kit from LGC Genomics GmbH (Berlin, Germany, https://www.lgcgroup.com/). The lysis buffer was additionally mixed with 1% thioglycerol and RNase. All DNA extracts were eluted in Tris buffer containing EDTA (10 mM Tris, 0.1 mM EDTA).

GBS was employed to generate genome‐wide polymorphism data (Elshire et al. 2011). The GBS library was prepared using the enzyme combination *Pst*I‐*Ape*KI, and pooling was conducted for 2 × 150 bp sequences on the Illumina NextSeq 500/550 v2. For library preparation, the LGC—Library preparation protocol (see Appendix S2) was followed. The objective was to achieve an average of 1.0 million reads per sample.

A total of 384 million raw reads were obtained across the entire data set. All library groups were demultiplexed using the Illumina bcl2fastq v. 2.20 software. The data were processed using the Illumina bcl2fastq v. 2.20 software, with the application of filters allowing for one or two mismatches or *N*_s_ in the barcode read, contingent upon the barcode distances between all libraries on the lane. Additionally, (1) no mismatches or *N*_s_ were permitted in the inline barcodes, although *N*_s_ were allowed in the restriction site. (2) Removal of residual sequencing adapters from all reads. (3) Elimination of reads with a final length of less than 20 bases. (4) Filtration of restriction enzyme sites at the 5′ end of reads. (5) Exclusion of all reads containing *N*_s_. (6) Trimming of reads at the 3′ end to achieve a minimum average Phred quality score of 20 over a window of 10 bases.

In the absence of a complete *Baccharis* genome, the “reference” sequence from Schneider and Hellwig (2024) was utilized, with a particular focus on the same four taxa. The software BWA‐MEM v. 0.7.12 (Li 2013) was employed for the purposes of alignment and reference creation.

The variant discovery and genotyping of samples was conducted using the Freebayes software v. 1.2.0 (Garrison and Marth 2012). The following specific parameters were employed: minimum base quality = 10, minimum supporting allele qsum = 10, read mismatch limit = 3, minimum coverage = 5, no indels, minimum alternate count = 4, exclude unobserved genotypes, ploidy = 2, no multi‐nucleotide polymorphisms (MNPs), no complex, mismatch base quality threshold = 10. The locus count was 63,348, with a mapping rate of 74.3%. A total of 30,773 polymorphic loci were identified, with a total of 120,064 SNPs across all samples. Subsequently, the variants were filtered using a GBS‐specific rule set with Freebayes v. 1.2.0, whereby the following criteria were applied: (1) the read count for a locus must exceed 8 reads; (2) genotypes must have been observed in at least 64 of the samples; (3) the minimum allele frequency across all samples must exceed 5%. This resulted in 4463 SNPs, which were used for further population genetic analysis (see Table S1 in Appendix S2). The data were stored in variant call format (vcf), as described by Danecek et al. (2011).

### 2.4 Linkage Disequilibrium

Linkage disequilibrium (LD) is a population‐based parameter describing the degree to which two alleles are linked and thus inherited together within a given population. Pruning of highly linked alleles can improve the correlation between principal components and geography as well as the quality of further genetic differentiation analyses (Abdellaoui et al. 2013). LD was tested with the R package GWLD v. 1.3.4 in R v. 4.0.3 (Zhang et al. 2023). Each species was tested for LD. SNPs with a reduced mutual information (RMI) value higher than 0.7 were removed, as in similar population genetic studies (see Henn et al. 2012). In *Baccharis linearis*, 248 SNPs were linked; in *B. macraei*, 277; and in *B. vernalis* , 384. In total, 739 SNPs were removed (some SNPs being identical in two or three species), ending up with 3724 SNPs for further analysis.

### 2.5 Sparse Non‐Negative Matrix Factorization—Clustering

To gain further insight into the genetic structure of the data set (comprising 378 individuals and 3724 SNPs), the samples were clustered using a non‐spatial, non‐Bayesian approach provided by the LEA package v. 3.14.0 in R v. 4.3.1. The sparse Non‐Negative Matrix Factorisation (sNMF) employs the least squares optimization and is capable of faster clustering than Bayesian methods such as the “structure” or “TESS 2.3” algorithms (Frichot et al. 2014). The sNMF is a model‐free approach that does not make any assumptions about the underlying biological processes. It is therefore robust to deviations from population genetic models, in contrast to the “structure” algorithm, which employs Hardy–Weinberg models (Pritchard et al. 2000).

The clustering was performed with varying numbers of clusters (*k* = 1–20), a regularization parameter *α* = 5, a tolerance parameter *ε* = 10^−4^, 5% of masked genotypes when calculating the cross‐entropy criterion, and 100 replicates per cluster. The fitting of the model and estimation of the number of clusters are based on the cross‐entropy value (Wold 1978; Eastment and Krzanowski 1982; Frichot et al. 2014). The CLUMPAK software was used to consolidate the outputs of the various runs (Kopelman et al. 2015). The optimal run of the selected clusters was visualized using the “pophelper” package (Francis 2017), the “dplyr” package (Wickham et al. 2020), and the “ggplot2” package.

### 2.6 Principal Component Analysis

A principal component analysis (PCA) was performed on the .vcf file, which was converted into a .geno file and an .indv file using the software pdg‐spider v. 2.1.1.5 (Lischer and Excoffier 2012). The principal component analysis (PCA) and plotting were conducted in R v. 4.3.1 (R Core Team 2023), utilizing the LEA package v. 3.14.0 for PCA (Frichot and François 2015) and the ggplot2 package v. 3.4.4 (Wickham 2016) for plotting.

### 2.7 Hybrid Index, Interclass Heterozygosity, and Triangle Plot

The percentage of heterozygous alleles (*S*_H_) was measured over all SNPs, excluding missing data. Frequency distribution of that percentage is presented as a violin plot for all taxa (*Baccharis linearis*, *B. macraei*, *B. vernalis* , and *B × intermedia*). Additionally, each locality was investigated separately and plotted using the package “ggplot2” v. 3.3.3 for all taxa. Furthermore, for *B. linearis* , *B. macraei*, and *B. vernalis* , the value *S*_H_ was plotted in comparison to the distance to the most northern sample location, forming a transect from north to south through central Chile.

To detect the generation of hybrids (F_1_, F_2_, …, F_*n*_) and potential backcrosses in the hybrid complex, the hybrid index and the interclass heterozygosity under Hardy–Weinberg Equilibrium were calculated with the standard settings using the package triangulaR v. 0.0.1 and plotted with the same package (Wiens and Colella 2024). *Baccharis macraei* and *B. linearis* were set as parental species. The allele frequency threshold was set to 0.999, leading to 230 ancestry‐informative markers that passed this threshold. Those 230 sites were then used to calculate the hybrid index and the interclass heterozygosity. The threshold from 0.999 was chosen to include the highly informative sites. A lower threshold would include more but less informative sites (Wiens and Colella 2024).

### 2.8 Genetic Differentiation

Another approach elucidating relationships and differentiation among populations is Nei‐F_ST_. Pairwise Nei‐F_ST_ considers mutation and genetic drift (Nei 1987). Both algorithms are applied as implemented in the package “hierfstat” v. 0.04‐22 in R v. 4.3.1. (Goudet and Jombart 2021). Furthermore, with the same package expected heterozygosity (*H*_e_), observed heterozygosity (*H*_o_) were calculated using Nei's (1987) methods. Also, the fixation index or inbreeding coefficient (*F*_IS_) was calculated for each population using (Nei and Chesser 1983) algorithm.

### 2.9 Tajima's *D* and Nucleotide Diversity (*π*)

Tajima's *D* (Tajima 1989) is a value to distinguish between sequences that evolve randomly (“neutrally”) and others that evolve under non‐random processes such as introgression, selection, demographic expansion, or shrinking. Tajima's *D* was calculated in R using the PopGenome v. 2.7.7 (Pfeifer et al. 2014) and calculated for each population. Nucleotide diversity (*π*) was calculated using vcftools v. 0.1.16. It was calculated for each population and SNP, and a mean was calculated for each population.

### 2.10 Isolation by Distance

To detect patterns of isolation by distance, genetic distances and geographic distances were compared. Therefore, pairwise *F*_ST_‐values (Nei's genetic distance) of each population were correlated to the geographic distance between those populations (Diniz‐Filho et al. 2013). Correlations between two matrices were tested using a Mantel test (Mantel 1967) with the Pearson correlation coefficient and 1000 permutations in the R package vegan v. 2.5.7 (Oksanen et al. 2024).

### 2.11 *f*‐Statistics and Patterson's *D*‐Statistics

To describe the relationships between populations, Patterson's *f*‐statistics were employed. To test whether a population is admixed, f3 and f4 were calculated. Negative f3 values correspond to a branch with negative genetic drift, which is not permitted under the null assumption and is indicative of hybridization (Peter 2016). f4 is a measure of the degree of shared drift between two population pairs. It is defined as the covariance of allele frequency differences in each pair. f3 and f4 partition genetic drift into two components: one specific to a single population and the other shared between two populations. Evolutionary relationships between those populations can be unraveled by measuring the shared drift between pairs of populations (Patterson et al. 2012; Peter 2016). *f*‐Statistics were calculated for different scenarios. (1) for each taxon at each location. (2) for each taxon at each location, excluding *Baccharis vernalis*. (3) for each taxon at each location, excluding *B. vernalis* and *B. × intermedia* (4) dividing the taxa into a north, south and “Quintay”‐cluster according to the clustering results. With scenario four, *B. vernalis* and *B. × intermedia* were excluded similarly as mentioned before. f3 and f4‐statistics were calculated with Treemix v. 1.13 (Pickrell and Pritchard 2012). In order to detect and measure hybridization, Patterson's *D*‐statistics were calculated with Dsuite v. 0.4 using Jackknife = 2000 (Malinsky et al. 2021). Dsuite also calculated the f4‐ratio, BBAA, ABBA, BABA, and f3. For calculating f3, the data were divided into 7 blocks of 500 bp each. An individual from the genus *Haplopappus* Cass. was chosen as an outgroup for the analysis.

### 2.12 Treemix

To construct a tree topology including potential hybridization events, Treemix v. 1.13 (Pickrell and Pritchard 2012) was utilized. The analysis was conducted with a range of 0–20 migration events, i.e., hybridization events, employing bootstrap with 500 repeats and sample size correction. Maximum likelihood was plotted to estimate the amount of migration events. The same outgroup as for *D*‐statistics and *f*‐statistics was selected. The Treemix tree and residuals were plotted in R v. 4.3.1. Treemix was performed for each taxon at each location separately as well as according to the clusters formed above in the *f*‐statistics. The analysis was performed for an unrooted tree and a rooted tree with *Baccharis linearis* as an outgroup.

## 3 Results

### 3.1 Sampling

Sampling showed that the habitat of *Baccharis macraei* extends from Huentelauquén in the north to Bucalemu in the south, spanning a distance of 340 km. The southernmost occurrence mentioned by Hellwig (1990) was on the beach of Topocalma. The species was not present north of Huentelauquén and was not seen south of Bucalemu to Putú. The dioecious species showed a sex ratio of 50% male and 50% female plants in most populations. However, in the Pichilemu transect in the south, the sex ratio was not maintained, and there were more female plants than males. The estimated ratio was 70% females to 30% males.

### 3.2 Genetic Relationships in *Baccharis* Populations

**FIGURE 2:**
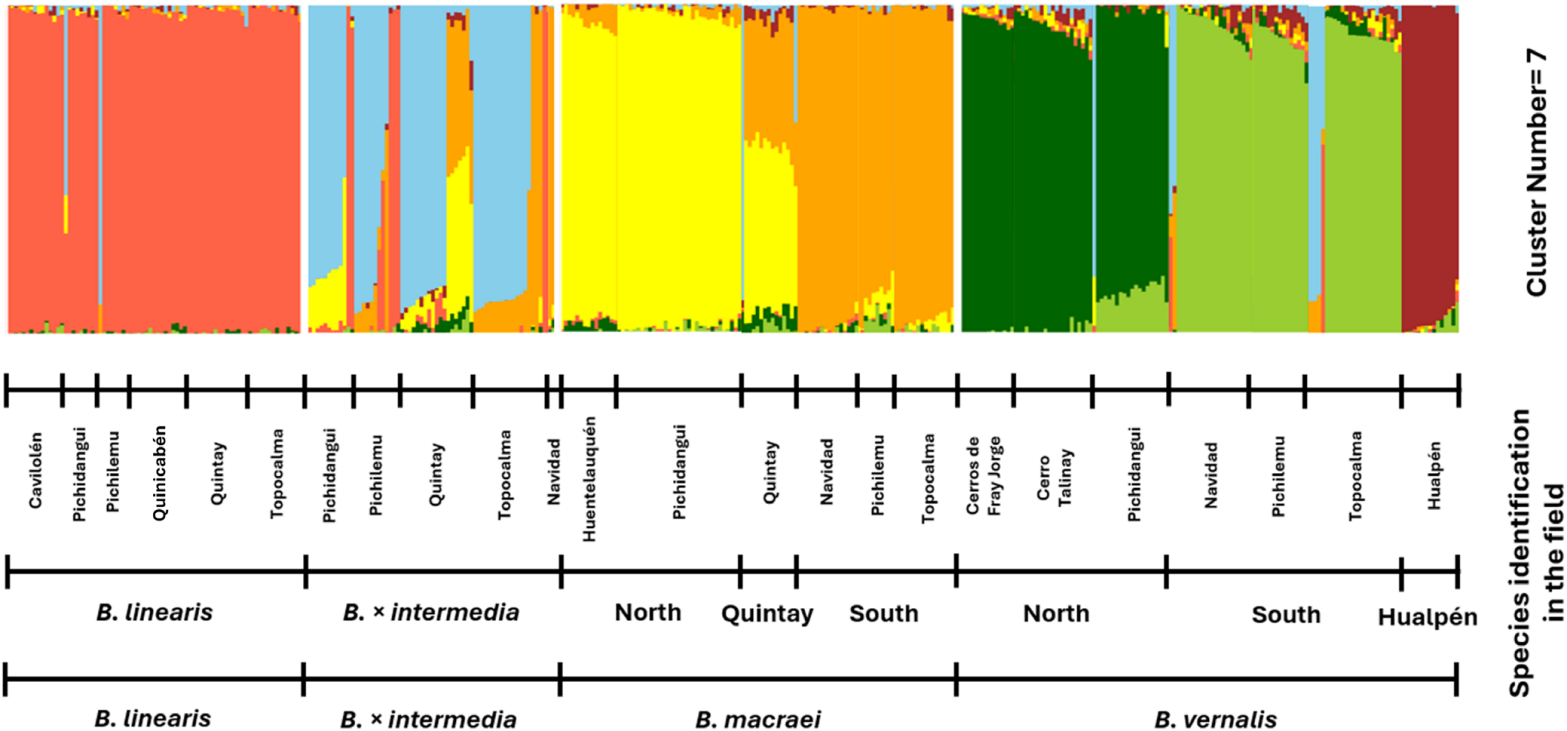
Genetic cluster assignment of the investigated *Baccharis* taxa in Chile shown as a barplot. The figure shows seven clusters. *Baccharis macraei* separates into two clusters (North and South) corresponding to its geographical distribution along the latitudinal gradient, while *B. vernalis* separates into three clusters (North, South, and Hualpén). The samples are ordered by preliminary field determination. Localities are ordered from left to right in a North–South sequence.

**FIGURE 3:**
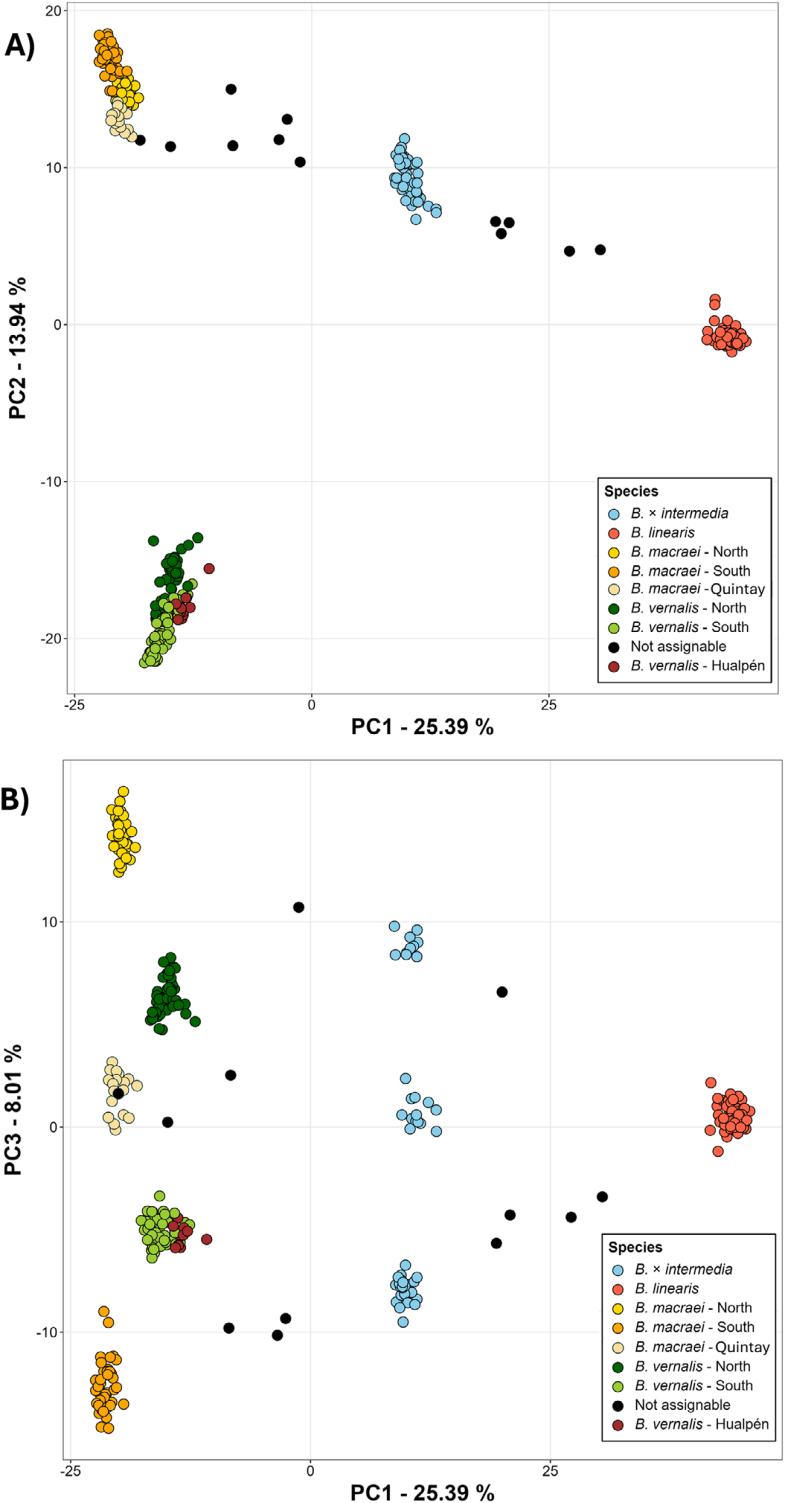
PCA results for samples of *Baccharis* in Chile. (A) Shown are axis PC 1 (25.39%) and PC 2 (13.94%) and (B) PC 1 (25.39%) and PC 3 (8.01%). Assignment of individuals is done according to sNMF‐clustering using *K* = 7 clusters; “Not assignable” was applied to individuals that belong more than 25% to a different group in the sNMF‐clustering. *Baccharis macraei*—Quintay population got a different pale‐yellow color in order to distinguish this population from the hybrid backcrosses in black.

In order to elucidate the genetic structure, a clustering analysis was initially conducted. Several numbers of clusters (*K* = 1–20) were tested. In the current study included also *B. vernalis* and four (*K* = 4) representing the parental species (*Baccharis macraei*, *B. linearis* ), *B. vernalis* and the hybrid or seven (*K* = 7) clusters were considered to be appropriate (see Figure S1 in Appendix S3). For four clusters, most of the samples were assigned to clusters corresponding to their corresponding a priori field determination (see Figure S2 in Appendix S3). Seven clusters divided *B. macraei* into two subgroups, showing geographical subdivision into a northern and a southern group. However, within *Baccharis* × *intermedia* as sampled in the field and labeled accordingly, several individuals from Quintay genetically belonged to the *B. macraei* group and not to the hybrid (see Figures 2 and 3). *Baccharis linearis* did not split into different clusters, while within *B. × intermedia* there were indications for existence of groups of genotypes (see Figure 3), that are, however, not recognized through sNMF‐clustering with *K* = 7. Furthermore, *B. vernalis* was divided into a northern and a southern group of populations, and the population in Hualpén in the extreme South.

PCA was used to get an overall impression of the structure of the data set. The different *Baccharis* species were well separated and formed distinct groups (Figure 3), including the putative hybrid *Baccharis* × *intermedia*, which lies midway between *B. macraei* and *B. linearis*. Specimens of *B. linearis* formed a densely packed cluster in this figure. In contrast, *B. macraei* and *B. × intermedia* formed three groups, each of which resembles a geographical region. The northern *B. macraei* group included specimens from Pichidangui and Huentelauquén. The southern group included specimens from Navidad, Pichilemu, and Topocalma. In between these two groups, there are the individuals from Quintay. These were separated from the other groups. The hybrid *B. × intermedia* was divided like *B. macraei*. Again, three subgroups had been observed, following the same pattern as for *B. macraei*. In the southern subgroup of *B. × intermedia*, seven backcrosses with the parental species had been observed, whereas in the North only two specimens showed signs of backcrossing (Figure 3). The clustering revealed two hybrids in the population at Navidad.

The most informative principal components (PC) in the PCA were PC 1 and PC 2 (Table S1 in Appendix S3). Axis 1 with a PC of 25.4% explains the largest portion of variation in this data set. In comparison, axis 2 has a PC of only 13.9%. Based on clustering and PCA, the transects were not treated as a continuum, but rather were divided into separate “populations”, thus reflecting taxonomic treatment of the individuals. The evolutionary paradigm of a population as a reproductive community was not used here, as *Baccharis linearis*, *B. × intermedia*, and *B. macraei* together are not in panmixia and are morphologically and genetically well separated (Waples and Gaggiotti 2006). A definition for a population suitable for this study would be: a local group of individuals from the same species (i.e., taxon having the same evolutionary history) and therefore sharing the same gene pool. Separating the parental species and the hybrid also allowed for obtaining further information about evolutionary processes in the parental species like e.g., potential introgression at the transect locations.

### 3.3 Genetic Differentiation

The value of the mean percentage of heterozygous SNPs (*S*_H_) was higher (27%–34%) in the hybrids than in the remaining taxa (12%–15%, see Table 2). In addition, the standard deviation (SD) was significantly higher than in the parent species as well as in *Baccharis vernalis*. Strong variation between the different locations could only be recognized in the hybrids. Plants collected near Navidad (*N*_S_ = 2) had a value of 27%, and plants collected near Pichilemu (*N*_S_ = 11) had a value of 28%, while plants collected at the other sites showed values from 33% to 34%. The values for nucleotide diversity (*π*) were similar. *Baccharis × intermedia* has the highest values between 28% and 30%. The values of the other taxa were 12%–17% (see Table 2).

**TABLE 2:** Genetic diversity parameters of *Baccharis × intermedia*, *B. linearis* , *B. macraei* and *B. vernalis* populations in Chile: Estimated population size (*N*_T_), number of sampled plants (*N*_S_), mean percentage of heterozygous alleles (*S*_H_) in the population and their standard deviation (SD); expected heterozygosity (*H*_e_), observed heterozygosity (*H*_o_), nucleotide diversity (*π*), inbreeding coefficient (*F*_IS_), and Tajima's *D*.

Population	*N*_T_	*N*_S_	*S*_H_ ± SD	*H*_e_	*H*_o_	*π*	*F*_IS_	Tajima's *D*
*B. × intermedia*								
Navidad	2	2	0.27 ± 0.005	0.55	0.88	0.28	−0.66	—
Pichidangui	50	12	0.33 ± 0.028	0.54	0.91	0.28	−0.68	−0.85
Pichilemu	50	11	0.28 ± 0.088	0.54	0.88	0.30	−0.61	−0.84
Quintay	100	16	0.33 ± 0.046	0.55	0.91	0.29	−0.66	−1.22
Topocalma	100	20	0.34 ± 0.035	0.54	0.91	0.29	−0.68	−1.44
*B. linearis*								
Cavilolén	100	16	0.13 ± 0.007	0.42	0.72	0.15	−0.65	0.32
Pichidangui	75–100	9	0.14 ± 0.006	0.43	0.73	0.14	−0.66	0.23
Pichilemu	300	9	0.13 ± 0.007	0.42	0.72	0.15	−0.66	0.06
Quinicabén	100	15	0.14 ± 0.007	0.43	0.73	0.15	−0.65	0.29
Quintay	150–200	15	0.14 ± 0.016	0.43	0.72	0.15	−0.65	0.14
Topocalma	100	16	0.14 ± 0.009	0.43	0.72	0.15	−0.65	0.04
*B. macraei*								
Huente‐lauquén	4000	15	0.13 ± 0.005	0.50	0.90	0.13	−0.78	0.35
Navidad	75	18	0.13 ± 0.006	0.50	0.89	0.12	−0.77	−0.07
Pichidangui	4000	32	0.14 ± 0.005	0.51	0.91	0.14	−0.76	0.17
Pichilemu	500	8	0.13 ± 0.005	0.50	0.89	0.16	−0.76	0.15
Quintay	150–200	21	0.14 ± 0.007	0.52	0.89	0.17	−0.70	−1.42
Topocalma	1500	19	0.13 ± 0.008	0.50	0.89	0.13	−0.76	0.13
*B. vernalis*								
Fray Jorge	10,000–12,000	14	0.15 ± 0.006	0.51	0.88	0.15	−0.72	0.17
Navidad	50	20	0.14 ± 0.007	0.51	0.87	0.14	−0.70	0.29
Pichidangui	200	19	0.14 ± 0.010	0.50	0.87	0.14	−0.73	0.37
Pichilemu	200	16	0.14 ± 0.007	0.51	0.88	0.14	−0.70	0.27
Talinay	10,000–12,000	21	0.15 ± 0.006	0.51	0.88	0.15	−0.70	0.25
Hualpén	200	15	0.12 ± 0.007	0.50	0.86	0.12	−0.72	0.65
Topocalma	1000	19	0.14 ± 0.007	0.51	0.87	0.15	−0.69	0.14

Observed heterozygosity (*H*_o_) was in every case higher than expected heterozygosity (*H*_e_) under Hardy–Weinberg equilibrium (HWE). Observed heterozygosity was highest in *B. × intermedia* (ranging from 0.88 to 0.91) and *B. macraei* (ranging from 0.89 to 0.91). *Baccharis vernalis* had values from 0.86 to 0.88, and the lowest values were reached in *B. linearis* with 0.72 to 0.73. Expected heterozygosity (*H*_e_) was also lowest in *B. linearis* ranging from 0.42 to 0.43 and highest in *B*. *× intermedia* with values from 0.54 to 0.55. *Baccharis vernalis* and *B. macraei* have values from 0.50 to 0.52.

The inbreeding coefficient (*F*_IS_) was negative in all taxa and locations. The maximum value was reached in the taxon *Baccharis × intermedia* in the population Pichilemu (*F*_IS_ = −0.61) and the minimum value in *B. macraei* in Huentelauquén (*F*_IS_ = −0.78).

The calculation of Tajima's *D* showed that all hybrids had a negative value. The values laid between *D* = −0.84 (Pichilemu) and *D* = −1.44 (Topocalma). Further negative values were calculated for *Baccharis macraei* in Quintay (−1.42) and in Navidad (−0.07). Values around 0 were calculated for *B. linearis* in Pichilemu (0.06) and Topocalma (0.04). Most other populations showed slightly positive values in the range of *D* = 0.14–0.29. Particularly high values were obtained for *B. linearis* in Cavilolén (0.32), *B. macraei* in Huentelauquén (0.35) and for *B. vernalis* in Pichidangui (0.37) and in Hualpén (0.65).

### 3.4 Percentage of Heterozygous SNPs

**FIGURE 4:**
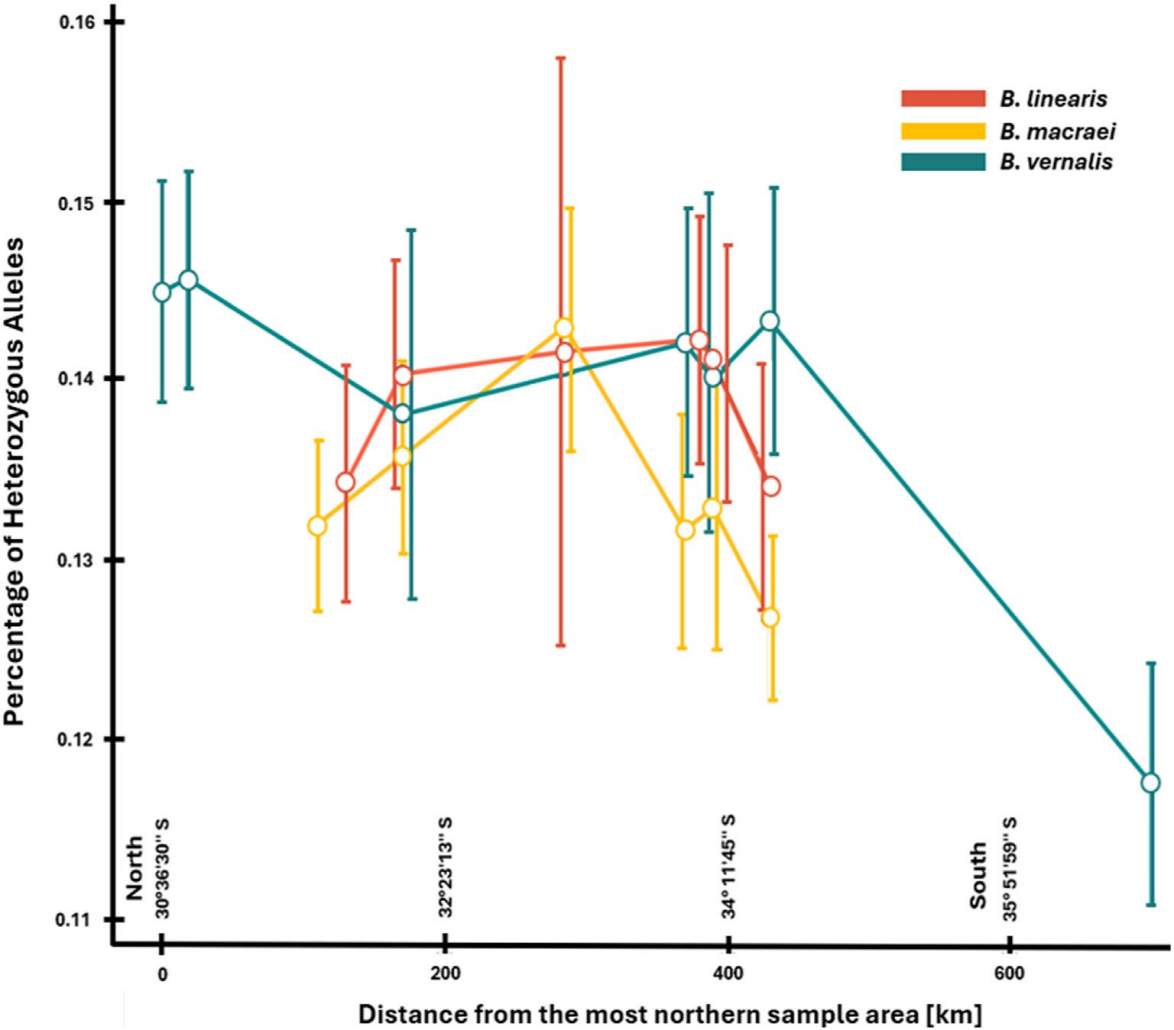
Percentage of heterozygous alleles by different taxa corresponding to their geographical distribution from North to South, excluding the hybrid *Baccharis × intermedia* for better visibility. Highest percentage for *B. vernalis* is in Talinay population, for *B. macraei* in Quintay, and for *B. linearis* in Quinicabén.

Percentage of heterozygous SNPs per individual was plotted as a violin plot using different groupings, as can be seen in Figures S1–S4 in Appendix S4. Mean and standard deviation were calculated for each location and taxa and plotted together with the distance to the northernmost location (or latitude; see Figure 4). The hybrid was excluded from this plot as its high heterozygosity makes it more challenging to identify trends in the parental species.

For *Baccharis vernalis*, Fray Jorge and Talinay in the north were the locations with the highest heterozygosity. This dropped slowly at the next site in Pichidangui, while the standard deviation was higher. Heterozygosity remained at a comparable value for the populations in Navidad, Topocalma, and Pichilemu and then dropped sharply towards Hualpén. An optimum function was seen for *B. macraei*. Heterozygosity was lower at the edges of the range in Huentelauquén and Pichilemu than in the center of the species distribution in Quintay. A similar pattern was observed in *B. linearis* , although Cavilolén and Quinicabén populations were not situated at the very edges of the range of that species in Chile. Within *B. linearis* , the mean value of heterozygosity and the standard deviation were also high in the center of the sampled region and were declining towards the northern and southern limits at Cavilolén (north) and Pichilemu (south).

### 3.5 Hybrid Index, Interclass Heterozygosity, and Triangle Plot

**FIGURE 5:**
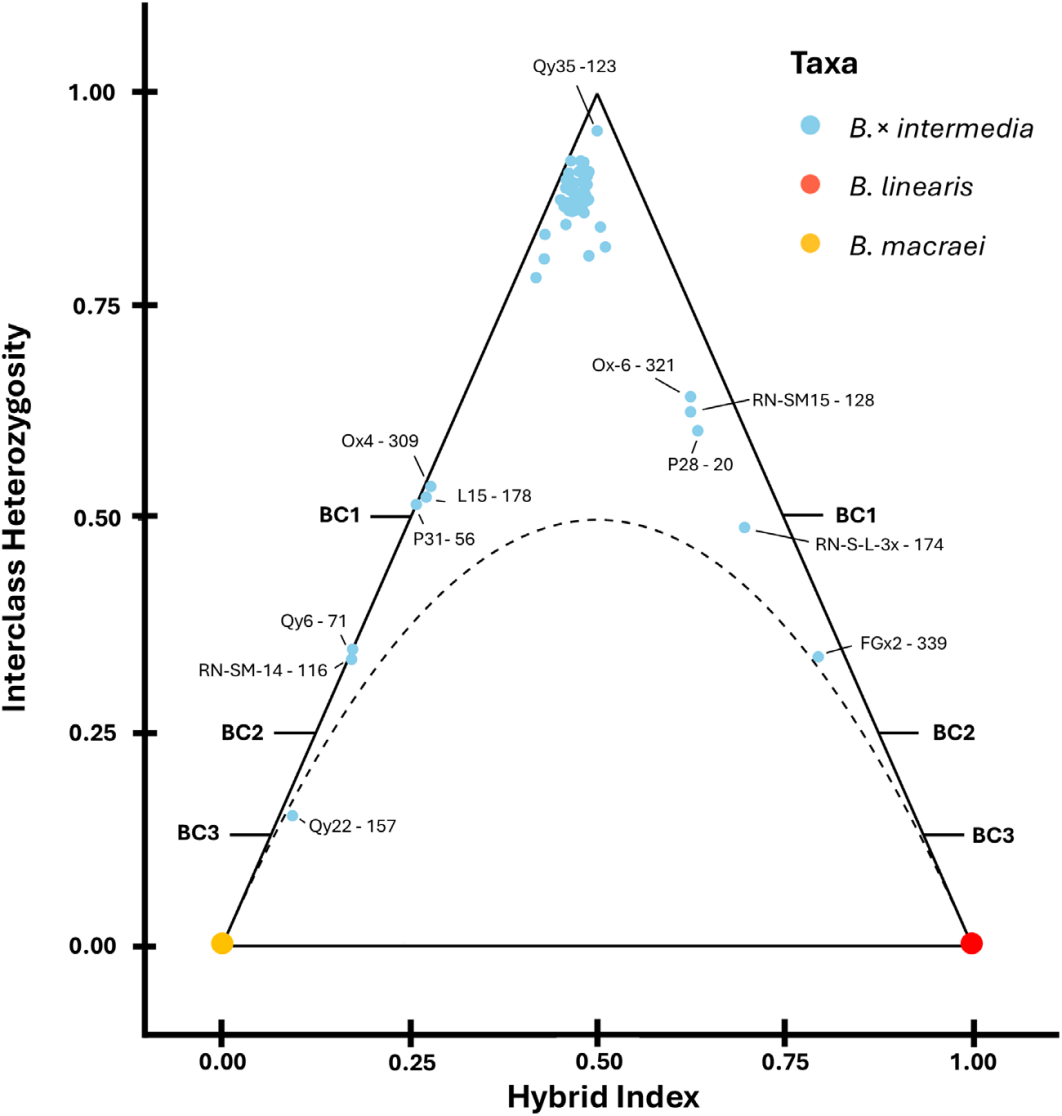
Triangle plot illustrating hybrid index and interclass heterozygosity under Hardy–Weinberg Equilibrium (HWE). Theoretical backcrosses with parental generations are marked additionally on the side. bc1 = First Generation Backcross, bc2 = Second Generation Backcross, bc3 = Third Generation Backcross. Parental taxa are *Baccharis macraei* and *B. linearis* . Hybrid taxa is *B. × intermedia* forming a lot of F_1_‐hybrids leaning more to *B. macraei*. Eleven backcrosses with the parental species were detected. Solid black lines indicate the possible space on a triangle plot, and the dotted black curve indicates the boundary below which individuals cannot occur, assuming Hardy–Weinberg Equilibrium (Wiens and Colella 2024).

A triangle plot was created to better describe the hybrid complex (see Figure 5). This combined hybrid index and interclass heterozygosity (proportion of loci with alleles from both ancestral taxa). The triangle plot can be used to recognize potential backcrosses, the filial generation, and possible hybrid speciation derived from this (Wiens and Colella 2024). Most hybrids are F_1_‐hybrids. These displayed typical high interclass heterozygosity. However, the hybrids with a medium hybrid index were shifted by 5% towards *Baccharis macraei*, i.e., they had 55% proportions of parental ancestry from *B. macraei* and only 45% of *B. linearis* . There was one hybrid “Qy35” from (Quintay), with the highest interclass heterozygosity, that has 50% proportions from both parental species. No F_*n*_‐hybrid population was detected.

Furthermore, numerous backcrosses to both parental species were observed. The backcrossing rate (number of backcrosses/number of hybrids) was 18%. Backcrossing took place in every transect investigated. Six backcrosses were observed in the direction of *Baccharis macraei* and five in the direction of *B. linearis* . Furthermore, no site preference was recognized. Since the number of backcrosses observed was too low, it is still unclear whether certain populations favor backcrosses to a certain parent species. The theoretical backcrosses of the first, second, and third order are shown in Figure 5 (see Wiens and Colella 2024). The hybrids “L15”, “L31”, and “Ox4” were presumably backcrosses of the first order with *B. macraei*. “Qy6” and “RN15” lay between theoretical backcrossing of the first order (bc1) and theoretical backcrossing of the second order (bc2) and were probably descendants of a backcrossing bc1 with an individual from *B. macraei*. “Qy22” was close to the backcrossing of the third order (bc3) with the parent species *B. macraei*. The individual “Rn‐S‐L3x” was probably a bc1 with the parent species *B. linearis* . “Ox6”, “P28”, and “RN‐Sm15” lay between bc1 and the F_1_‐hybrid population. It was assumed that these were backcrosses of a bc1 with the primary hybrids. The individual “FGx2” lay between bc1 and bc2 of *B. linearis* and was probably a descendant of the latter.

### 3.6 Isolation by Distance

Pairwise Nei‐*F*_ST_ was calculated and is shown in Tables S1–S6 in Appendix S5. Genetic distances were calculated for each population as well as for the four clusters representing the four taxa (*Baccharis macraei*, *B. linearis* , *B. vernalis* , and *B. × intermedia*) and for the division proposed for seven clusters. For the four taxa, the results did not differ from Schneider and Hellwig (2024). *Baccharis linearis* had the highest *F*_ST_ values to *B. macraei* (0.151–0.179), followed by *B. vernalis* (0.147–0.165). In *B. macraei*, the lowest value was found in Quintay, and for *B. vernalis* , it was in Talinay. The distance between *B. macraei* and *B. vernalis* was only 0.053–0.092. They were most similar between Quintay and Talinay and least similar between Hualpén and Huentelauquén. The difference to the hybrid was on average 0.088 to *B. macraei* and 0.108 to *B. linearis* . Nei's genetic distance was tested for isolation by distance using a Mantel‐Test. The distance matrix (see Tables S1–S3 in Appendix S6) between the populations was measured by spherical geometry. For all three species *Baccharis linearis*, *B. macraei*, and *B. vernalis* , the Mantel‐Test was significant (see Figure S1 in Appendix S6). The Pearson correlation coefficient (*r*) was 0.93 for *Baccharis linearis*, 0.94 for *B. macraei*, and 0.91 for *B. vernalis* .

### 3.7 *f*‐Statistics and Patterson's *D*

A total of 608 combinations were significant for the f3 statistics. Regarding the loci that were suitable for indicating introgression, only 22 negative values were reported in this study (see Tables S10–S12 in Appendix S7). All tested scenarios showed a similar topology ((*Baccharis × intermedia*—population, *B. linearis*—population), *B. macraei*—population). This indicated that the *B. × intermedia* populations are mixed from the parent populations. There were 1222 significant topologies in the f4 statistics. Only the 22 most positive values were reported. All scenarios followed the topology (((A, B), C), D). Significant negative values indicated gene flow between either C and B or D and A. Significant positive values indicated gene flow between A and C or B and D. F4‐ratios showed that there is intraspecific gene flow between the individual populations of the species (see Tables S1–S9 in Appendix S7). For example, there was potential gene flow between *B. linearis* –South and *B. linearis* –Quintay or between *B. vernalis*–South and *B. vernalis* –Hualpén.

Patterson's *D* was calculated both for the cluster groups (see Figure 2 and Figure S2 in Appendix S3) and for each population individually (see Tables S1–S9 in Appendix S7). The results can be found in Table 3. For reasons of space, only the significant values are recorded here. Significant values were presented for all hybrid populations with their parental species. Furthermore, significant *D*‐values (*D* = 0.08–0.24) were found between *B. macraei–*Quintay and all *B. linearis* populations. Furthermore, significant values were calculated between *B. linearis* and the northern *B. vernalis* populations (*D* = 0.12–0.15), as well as for *B. linearis*. Furthermore, there were significant values (*D* = 0.08–0.09) for *B. macraei*–Topocalma with *B. vernalis* –Pichilemu and *B. vernalis*–Navidad, respectively.

**TABLE 3:** *D*‐statistics and f4‐ratio of different *Baccharis* populations in Chile.

P1	P2	P3	*D*	*Z*‐score	*p*	f4‐ratio	BBAA	ABBA	BABA
*B. macraei* South	*B. macraei* Quintay	*B. linearis* North	0.24	9.07	0.00	0.07	184.26	35.07	21.32
*B. macraei* South	*B. macraei* Quintay	*B. linearis* Quintay	0.22	8.29	0.00	0.06	183.79	34.98	22.16
*B. macraei* North	*B. macraei* Quintay	*B. linearis* South	0.19	7.64	0.00	0.06	179.52	35.16	23.72
*B. macraei* South	*B. macraei* Quintay	*B. linearis* South	0.19	7.58	0.00	0.06	182.55	34.53	23.06
*B. macraei* North	*B. macraei* Quintay	*B. linearis* North	0.20	7.41	0.00	0.06	180.01	34.65	22.90
*B. macraei* North	*B. macraei* Quintay	*B. linearis* Quintay	0.20	7.22	0.00	0.06	180.13	35.17	23.44
*B. vernalis* South	*B. macraei* Quintay	*B. linearis* South	0.09	4.54	0.00	0.04	137.55	42.36	34.77
*B. vernalis* South	*B. macraei* Quintay	*B. linearis* Quintay	0.10	4.26	0.00	0.04	137.79	41.87	33.97
*B. vernalis* South	*B. macraei* Quintay	*B. linearis* North	0.08	3.46	0.00	0.03	137.30	40.50	34.29
*B. vernalis* North	*B. macraei* Quintay	*B. linearis* South	0.08	3.18	0.00	0.03	135.2	38.88	33.10
*B. macraei* Quintay	*B. macraei* South	*B. vernalis* South	0.09	3.77	0.00	0.17	88.39	42.59	35.52
*B. macraei* North	*B. macraei* South	*B. vernalis* South	0.08	3.22	0.00	0.16	89.90	41.21	34.60
*B. macraei* South	*B. vernalis* North	*B. linearis* North	0.15	4.07	0.00	0.05	140.32	36.55	26.68
*B. macraei* North	*B. vernalis* North	*B. linearis* North	0.12	3.46	0.00	0.04	140.99	34.43	26.55
*B. macraei* South	*B. vernalis* South	*B. linearis* North	0.11	4.02	0.00	0.04	146.94	36.40	28.865
*B. macraei* South	*B. vernalis* Hualpén	*B. linearis* North	0.17	4.14	0.00	0.06	138.07	40.00	27.80
*B. macraei* South	*B. vernalis* Hualpén	*B. linearis* Quintay	0.14	3.36	0.00	0.05	137.66	38.77	28.80
*B. macraei* North	*B. vernalis* Hualpén	*B. linearis* North	0.14	3.21	0.00	0.05	134.11	41.29	31.08
*B. macraei* South	*B. vernalis* Hualpén	*B. linearis* South	0.11	3.11	0.00	0.04	137.03	38.74	30.97

*Note:* *Haplopappus* was used as an outgroup (without *B. × intermedia*).

Abbreviations: *D*, Patterson's *D*; P1, Population 1; P2, Population 2; P3, Population 3.

### 3.8 Treemix

**FIGURE 6:**
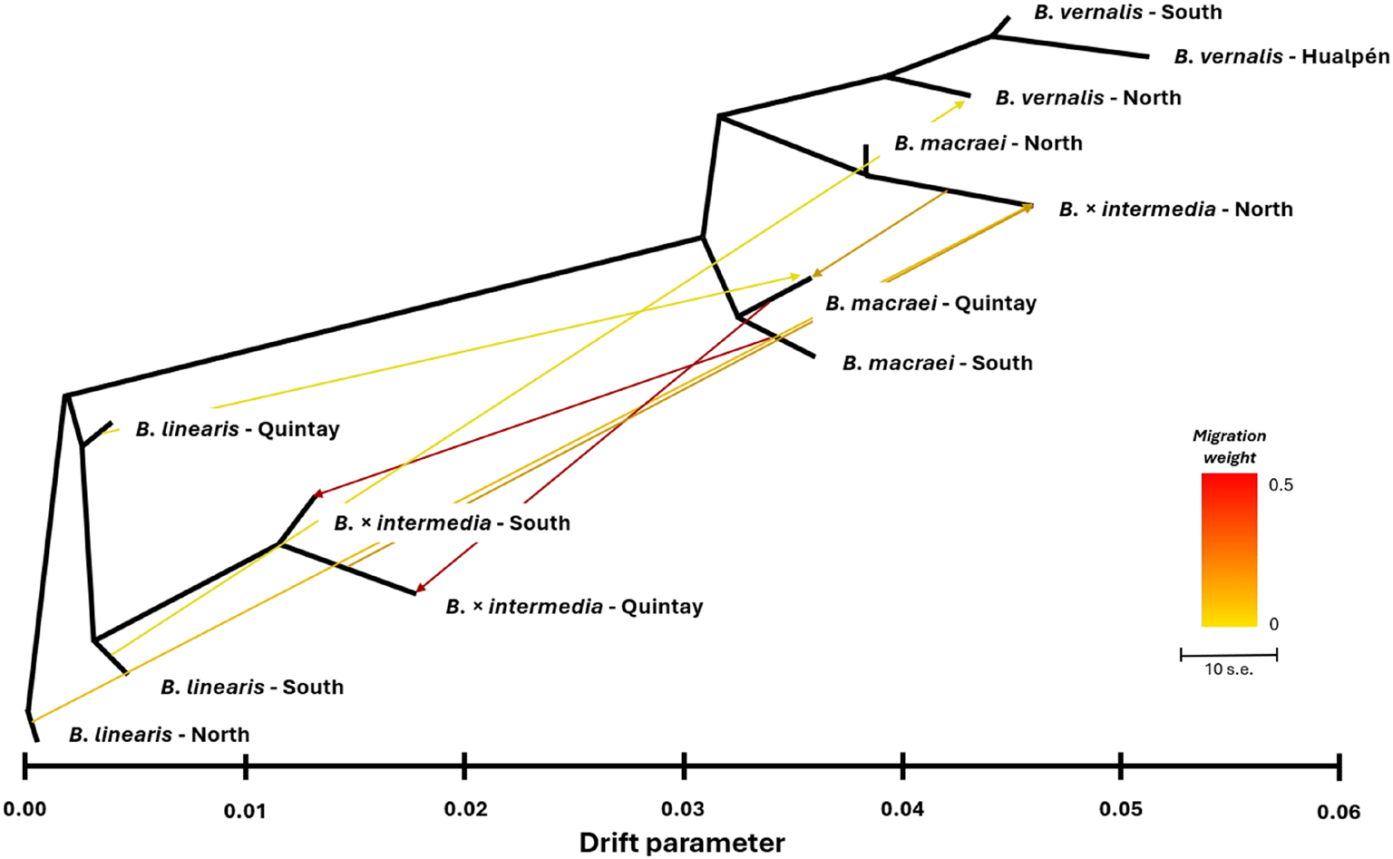
Maximum likelihood tree including seven migration events between *Baccharis linearis*, *B. macraei*, *B. vernalis* , and *B. × intermedia*. The arrow is colored by migration weight, and branch lengths are proportional to genetic drift. The topology indicates a close relationship between *Baccharis macraei* and *B. vernalis* . *Baccharis linearis* was chosen as the outgroup, similar to Schneider and Hellwig (2024).

In the Treemix analyses, scenarios with different numbers of migration, i.e., hybridization events were tested. The test for every population separately suggested a minimum of 20 hybridization events between the populations (see Figure S1 in Appendix S8). For the sake of clarity, the populations were grouped again according to the clustering results, but still separating the populations in Quintay, as well as dividing the hybrid populations into “North”, “South” and “Quintay” groups. For this scenario, seven migration events were assumed. Introducing further migration events increased the likelihood only marginally (see Figure S2 in Appendix S8). *Baccharis linearis* in this analysis was also separated from the other groups that stand closer to each other than to *B. linearis* . *Baccharis vernalis* was closely related to *B. macraei* in this topology. The values of the drift parameter indicated that *B. macraei* (North and South) and *B. vernalis* originated in close temporal sequence followed by extended evolution within the different clades. The topology of the tree shows *Baccharis × intermedia* diverged between the parental species. *Baccharis* × *intermedia*–South and –Quintay were placed in the *B. linearis* category, while the *B. × intermedia*–North was linked to *B. macraei* in this topology (see Figure 6). *Baccharis* × *intermedia*–North received gene flow from *B. linearis* –North as well as from *B. linearis* –South. *Baccharis* × *intermedia*–Quintay got gene flow from the corresponding *B. macraei* population in Quintay. *Baccharis × intermedia*–South had a migration event coming from *B. macraei*–South. In general, each hybrid received gene flow from the parent species from which it was most distant in the topology. Furthermore, the following gene flows were observed: *Baccharis macraei*–Quintay received gene flow from *B. linearis* –Quintay and *B. vernalis*–North received gene flow from *B. linearis*–South.

## 4 Discussion

Intensive hybridization was observed between *Baccharis macraei* and *B. linearis* . This was also detected previously in the study by Schneider and Hellwig (2024). The present study represents the first investigation of this phenomenon across the entire distribution range of the hybrid zone, employing a comprehensive field sampling approach to characterize the hybrid complex in its entirety. Such studies using multiple transects are especially well suited to reveal the genetic substructure of the parental species as well as the hybrid species (Harrison and Larson 2016). So far, they are still relatively rare even in more intensely investigated taxa like *Salix* (Marinček et al. 2023). Moreover, it should be emphasized that, due to the limited geographical distribution of *B. macraei* and the study's design, the entire genetic variability of the species is encompassed. In contrast, in *B. linearis* , only a fraction of the genetic variability is present in the contact zone.

*Baccharis vernalis* showed no connection to the hybrid *B. × intermedia*, and no hybrids between *B. vernalis* and the other species have been observed. Schneider and Hellwig (2024) detected one individual that may represent a hybrid between *B. vernalis* , *B. linearis* , and/or *B. macraei*. In the light of the present study, the hybrid *B. × septentrionalis* ( *B. vernalis* *× B. macraei*) appears to be an extremely rare occurrence. Neither field observations nor genetic sampling of the entire distribution range of *B. macraei* and *B. vernalis* gave further evidence for *B. × septentrionalis*. The rarity of this hybrid may be due to the non‐overlapping flowering periods of the two putative parental species. However, exceptional flowering off the season has been reported earlier (Hellwig 1990), which could facilitate the occurrence of rare hybridization events.

All four taxa under investigation were clearly separated by both PCA and clustering (see Figure 3). *Baccharis macraei* was split into a northern and southern cluster, with the population at Quintay in an intermediate position. This population is (as far as sampled) composed of individuals showing admixture from both clusters (see below). A north–south differentiation within the species was already reported by Schneider and Hellwig (2024) and Zafra‐Delgado et al. (2025). Likewise, *Baccharis vernalis* splits up into a northern and southern cluster.

Furthermore, polytopic origins can be assumed for *Baccharis × intermedia* based on genetic data. The individuals assembled under this name cluster into northern (Pichidangui), central (Quintay), and southern (Topocalma, Pichilemu) groups (see Figure 3). This is caused by the north–south differentiation in *B. macraei*, while no structuring could be recognized for *B. linearis* . Latitudinal structuring of genetic diversity has been shown for several taxa in Chile (see Villagrán and Hinojosa 2025 for examples and discussion of underlying dynamics in regional vegetation during the Pleistocene and Holocene).

The lack of spatial differentiation in the genome of *Baccharis linearis* indicates either extensive admixture among populations, or relatively recent range expansion, or both. The significantly lower genetic variation within *B. linearis* is also reflected in the low *F*_ST_–value (see Table S4 in Appendix S5). This suggests a high degree of interconnectivity between the populations in question, which are situated in close proximity to one another, thereby facilitating gene flow. Perhaps this is one reason why the observed heterozygosity is lower than in the other taxa (see Table 2). These results fit well with the theory that the expansion of *B. linearis* habitat happened rather recently after the destruction of the primary forest in Central Chile (Balduzzi et al. 1982; Hellwig 1990; Armesto and Arroyo 2007; Schulz et al. 2010). Destruction of sclerophyllous forest in Central Chile and the transformation and degradation of vegetation cover by farming and overgrazing originated many disturbed places, where hybrids may find favorable conditions to persist (Anderson 1949; Grant 1976; Grabenstein and Taylor 2018).

The mean percentage of heterozygous alleles (*S*_H_) was plotted according to the latitude (Figure 4). An optimum can be described for *Baccharis macraei*. *S*_H_ is lowest at the periphery and highest at the centre of the range. It is evident that *Baccharis* is dioecious; therefore, discrepancies in heterozygosity cannot be explained by inbreeding, but rather, they are indicative of genetic drift occurring within small populations. This finding is consistent with the theory that the proportion of heterozygous alleles decreases with increasing strength of the edge effect at the periphery, due to founder events, isolation, or limited outbreeding (Cook 1961; Logan et al. 2019; Cisternas‐Fuentes and Koski 2023). The latter can be excluded here because of dioecy. The amount of heterozygous alleles is maximum at Quintay (see Figure 4). There are different ways of explaining this pattern. It could be that already diverged northern (Huentelauquén, Pichidangui) and southern lineages (Navidad, Topocalma, Pichilemu) of *B. macraei* mix at Quintay, leading to higher heterozygous individuals. An alternative hypothesis suggests that the region surrounding Quintay (situated along the coastline around Valparaíso) represents an ancestral population from which the northern and southern populations are derived. Nevertheless, no definitive statement can be made about the direction of gene flow. In order to obtain a conclusive picture, further independent investigation methods such as SDM or MSMC are required.

In *Baccharis vernalis* the highest levels of heterozygosity (*S*_H_) were observed in the northern locations, specifically Fray Jorge and Talinay. The decline was gradual at the subsequent site, Pichidangui. However, the standard deviation is higher in this instance. The heterozygosity of the populations in Navidad, Topocalma, and Pichilemu remains at a comparable value, before dropping towards Hualpén. The elevated values observed in the northern populations may be attributed to a presumed ancient hybridization event with *B. linearis* or another *Baccharis* species (see Treemix, *D*‐stastic and Figure 6). Alternatively, the ancestral populations of *B. vernalis* were situated in the northern region and subsequently migrated southwards. Consequently, the edge effects exerted by this dispersal pattern have resulted in the observed reduction in heterozygosity values in the southern region, specifically in Hualpén. Support for the second hypothesis may come from vegetation history and research of genetic diversity patterns in other taxa in the region, such as *Aextoxicon punctatum* (Núñez‐Ávila and Armesto 2006) and *Drimys winteri* (Jara‐Arancio et al. 2012) where highest values of genetic diversity have been found in the northernmost populations.

The observed heterozygosity was found to be significantly higher than the expected heterozygosity (based on HWE) in each of the investigated populations (see Table 2). This suggests a high level of genetic diversity within these populations. In the case of *Baccharis linearis*, the difference between the observed and expected heterozygosity was at its lowest, at approximately 0.3, while in the other taxa the difference was approximately 0.4. This is also reflected in the inbreeding coefficient (*F*_IS_). The results were consistently negative across all populations, with values ranging from −0.61 to −0.78. In general, high negative *F*_IS_ values are indicative of an excess of heterozygous sites and may be indicative of outbreeding (Llambí et al. 2020). It is possible that past and present inter‐ and intraspecific admixture events may have contributed to these high values. Furthermore, it should be noted that dioecy in *Baccharis* is a factor that promotes heterozygosity (The observed sex ratio in the field, which is approximately 1:1, indicates that sex is genetically determined). This may contribute to an increased level of heterozygosity in comparison to hermaphrodite species (Muyle et al. 2021; Nakamura et al. 2021).

For the following populations, Tajima's *D* values were found to be within the range of ±0 (see Table 2). For *Baccharis macraei* sampled in Navidad (−0.07) and *B. linearis* sampled in Pichilemu (0.06) and Topocalma (0.04), the observed variation was equal to the expected variation. This indicates that the populations are potentially evolving in accordance with the mutation‐drift equilibrium, and that no selection should occur (Tajima 1989). Most other populations exhibited slightly positive values within the range of 0.14–0.29. Notably, the highest values were observed in *B. linearis* from Cavilolén (0.32), *B. macraei* from Huentelauquén (0.35), and *B. vernalis* from Pichidangui (0.37) and Hualpén (0.65). This indicates that these populations are lacking rare alleles, which is likely due to selection, founder effects, or a sudden contraction of the population size (Tajima 1989). Additionally, populations in the northern regions (Cavilolén, Pichidangui, and Huentelauquén) may also be affected by overgrazing, a phenomenon that was frequently observed during the field trip.

The results of the triangle plot (see Figure 5) demonstrated that 82% of the hybrid samples exhibited the characteristics associated with F_1_‐hybrids, as indicated by the hybrid index and interclass heterozygosity. Therefore, it can be concluded that hybridization is occurring repeatedly and there are no indications of hybrid speciation (e.g., a reduction in heterozygosity while the hybrid index remains unchanged, see Wiens and Colella 2024). Nevertheless, despite the occurrence of backcrossing, the complete dissolution of species boundaries between the parent species and the hybrid, or panmixia, is not observed in this hybrid complex. The three groups, namely *Baccharis macraei*, *B. linearis* , and the F_1_‐hybrids, can be effectively distinguished both morphologically and genetically. There is evidence for the existence of various backcrosses; some of them can only be inferred from the presence of the observed backcrosses. The inferred but not observed backcrosses were either not sampled or have already perished, as several generations of the perennial, woody *Baccharis* plants coexist in the same location.

The lack of F_2_‐hybrids (i.e., F_1_ × F_1_ crosses) may be explained by the large number of individuals belonging to the parental species in the immediate neighborhood of F_1_‐hybrids. Thus, it is very unlikely that a female F_1_ individual will be pollinated with pollen of another F_1_‐hybrid rather than by pollen of either *B. macraei* or *B. linearis* . This kind of argumentation was already introduced by Anderson (1949) and explained by Stebbins (1959) (cp. Yakimowski and Rieseberg 2014). This concept is further supported by the generalists' pollinators of these plants, such as diptera, hymenoptera, and coleoptera, leading to a lack of assortative mating. If F_2_ individuals occur at all, they will be rare and just by chance have not been sampled by us.

F_1_‐hybrids seem to have higher proportions of *Baccharis macraei* than *B. linearis* , as can be seen in the hybrid index. Similar differences in the F_1_‐hybrids' parental proportions have been observed in the genus *Psoralea* L. and *Fundulus* Lacépède (Bello et al. 2018; Hardy 2022). These discrepancies may be attributed to variations in genome size between the parental lineages or an artifact associated with the SNP variation. Genome size differences between several *Baccharis* species, including *B. linearis* and *B. macraei*, were observed by Schneider (2025). Furthermore, it should be noted that *B. macraei* exhibits greater genetic diversity than *B. linearis* . In total, *Baccharis macraei* has 16% invariant SNPs, whereas *B. linearis* has 69% invariant SNPs in this data set. This is also reflected in the Nei‐*F*_ST_ values between the species, which lends support to the hypothesis that the shift is caused by one of the parental species exhibiting greater genetic diversity than the other.

The hybrid complex is evidently in a state wherein hybrid fitness on one hand permits formation of backcrosses, while on the other hand it seems that due to restricted gene flow between the primary hybrids and parental species there is no panmixia among both parental species. However, hybrid fitness is also not sufficiently low to result in the complete separation (e.g., through reinforcement) of the parental species. The number of backcrosses (18%) with both parental species indicates that hybrids are generally not unfit. But other (esp. environmental) selective pressures may lead to the lack of F_2_‐hybrids as was observed in *Populus* L. (Roe et al. 2014; Lindtke et al. 2014). The fact that no F_2_‐hybrids have been detected in our study may be due to the relatively recent origin of the hybrid population or even resulted from a sampling bias as reported by Gramlich et al. (2018) in a hybrid population of two willow (*Salix* L.) species on a glacier forefield. They found that all sampled hybrid individuals were F_1_ plants, perhaps due to the sampling strategy which avoided sampling juvenile plants. The young age of the hybrid population (20–30 years old) may be responsible for the lack of adult F_2_ and later backcrosses in the samples. Such deficiencies have been reported from different plant groups and regions, e.g., for oaks in North America (Nason et al. 1992), *Rhododendron* in NE Turkey (Milne et al. 2003), or *Populus* in Europe (Bialozyt et al. 2012).

In contrast in *Salix* Fogelqvist et al. (2015) and Marinček et al. (2023) observed lack or deficiency in F_1_‐hybrids in different hybrid swarms a fact they explained with a long‐term persistence of the hybrid population or with limited recent hybridization or just conditions unfavorable to hybridization. The underlying causes or mechanisms preventing formation of later generation hybrids but rather foster backcrosses with and introgression to parental species are discussed by many authors (for overview see Mallet 2005, 2007; Rieseberg and Willis 2007; Yakimowski and Rieseberg 2014). In general fitness of hybrids may be compromised by internal factors. However, the extent of introgression in the individual populations cannot be comprehensively estimated and should be examined more closely, particularly in larger populations, using larger sample sizes.

Genome size differences in combination with potential chromosomal rearrangements in hybrids may lead to an increased hybrid load and compromise the fitness of subsequent hybrid generations. Further generations are expected to contain more deleterious variations compared with F_1_ generations (Rieseberg et al. 1999; Burton et al. 2006). It would be advisable to undertake a detailed examination of the fitness distribution in the hybrid complex in order to gain a comprehensive understanding of the current state and to make informed predictions for evolutionary perspectives.

It is possible that ecological barriers may prevent panmixia even at high fitness. *Baccharis macraei* is found in the azonal coastal vegetation of central Chile (Hellwig 1990). The high salinity of the site may act as a barrier to gene flow, maintaining species identity. In contrast, *Baccharis linearis* is adapted to the hinterland. It is possible that hybridization results in a hybrid where the genetic part of *B. macraei* is not as dominant as that of *B. linearis* . Such maladaptation to the coastal zones would be the inevitable consequence. Indeed, no or almost no hybrids are observed in populations of *B. macraei* on sand dunes in close proximity to the beaches. In a comparable situation, Burge et al. (2013) found evidence for selection against hybrids in edaphic conditions where only one of the parental species is adapted to a special soil type, the other being soil‐generalist in the genus *Ceanothus* (Rhamnaceae). Consequently, this may contribute to the maintenance of the hybrid zone, as ecogeographic isolation has long been regarded as the most significant reproductive barrier in plants (Rieseberg and Willis 2007). An example of a similar situation to the species group studied here was reported in *Encelia* species in Baja California by Kyhos et al. (1981). Here again one species inhabits coastal dunes while the others occur in areas more distant from the coastline. Hybrids are found in the lee side of coastal dunes, i.e., in between both ranges. This is paralleled in our present study. Hybrids are never found within the range of the more specialized salt‐tolerant parent but are found in adjacent areas often mixed with the other generalist parent ( *B. linearis* in our case). Soil salinity tolerance in *B. macraei* and drought tolerance of *B. linearis* may limit expansion of the hybrid zone, while F_1_‐hybrids are fit enough to persist in between and cause introgression into both parents. Recently, this situation was revisited and further studied by DiVittorio et al. (2020). As in Baccharis, in Encelia a narrow cline was identified mirroring edaphic conditions. Like in Encelia, we found limited traces of introgression more distant from the proper zone of contact between both parental species. Indeed, introgression can be observed in the parental species in Baccharis. Evidence of this was found between *Baccharis macraei*–Quintay and the *B. linearis* populations indicated by the presence of signals (Treemix, *D*‐Statistics, *f*‐Statistics) for such a process (see Table 3 and Figure 6). Furthermore, the *B. macraei* population in Quintay showed an abundance of rare alleles, which is indicated by a Tajima's *D* value of −1.42. Checking the loci, it becomes clear that these alleles have originated from *B. linearis* , further supporting the hypothesis of introgression. Furthermore, in Treemix, introgression was detected between *B. vernalis* –North and *B. linearis* . Morphological characteristics of northern populations of *B. vernalis* exhibit notable divergence from those observed in southern populations. For example, northern populations exhibit a cuneiform leaf shape, whereas southern populations display an obovate to orbicular shape (Hellwig 1990, unpublished data from the author). However, due to the scarcity of recent hybridization between both taxa (*B. vernalis* flowering in spring while *B. linearis* flowers in late summer and autumn, see Hellwig 1990), further research should be conducted to clarify this potential hybridization event. Testing Nei‐*F*_ST_ for isolation by distance showed that genetic distances among *Baccharis linearis*, *B. macraei*, and *B. vernalis* populations are strongly correlated to their geographical distance. Therefore, assuming strong anthropogenic alteration of the population composition, e.g., through transport of genetic material over large distances, is not plausible, even if *B. linearis* most probably expanded its range following roadsides and taking advantage of disturbance caused by e.g., road construction to establish new populations.

To briefly summarize the main findings of this study: *Baccharis × intermedia* consists of mainly F_1_‐hybrids with 18% backcrossing to both parental species. There is no indication of hybrid speciation. *Baccharis vernalis* is not involved in recent hybridization with the other two species *B. macraei* and *B. linearis*. Furthermore, two instances of introgression were identified, one involving *B. linearis* and (possibly) *B. vernalis* and the other involving *B. linearis* and the Quintay population of *B. macraei*. In further studies, genome size estimation and sequencing should be used to clarify the closer relationship of the hybrid to *Baccharis macraei*. However, even in the presence of backcrossing, no panmixia is observable in this hybrid complex. It would be advisable to take a closer look at the fitness distribution and environmental limitations in the hybrid complex in order to further characterize the state of the complex.

## Author Contributions

**Fabian Schneider:** formal analysis (lead), investigation (equal), methodology (equal), resources (lead), software (lead), validation (lead), visualization (lead), writing – original draft (lead). **Olga Zafra‐Delgado:** data curation (equal), investigation (equal), resources (equal), writing – review and editing (equal). **Tobias G. Köllner:** conceptualization (equal), funding acquisition (equal), investigation (equal), project administration (equal), resources (equal), supervision (supporting), writing – review and editing (supporting). **Frank Hellwig:** conceptualization (equal), data curation (equal), funding acquisition (equal), methodology (equal), project administration (equal), resources (equal), supervision (lead), writing – review and editing (lead).

## Conflicts of Interest

The authors declare no conflicts of interest.

## Supporting information


**Data S1:** ece372249‐sup‐0001‐DataS1.docx.

## Data Availability

Individual genotype and metadata are available on Max Planck Society's Edmond data repository (https://doi.org/10.17617/3.MWU9ML). Benefits Generated: Benefits from this research accrue from the sharing of our data and results on public databases as described above. More broadly, our group is committed to international scientific partnerships, as well as institutional capacity building.
