# Supplementary material for: Unraveling Evolution in the Homoploid Complex of Baccharis L. in Chile
Source: Ecol Evol. 2025 Oct 7;15(10):e72249. doi: 10.1002/ece3.72249 (PMC12501958; doi:10.1002/ece3.72249)

## Supplementary Material

**Appendix S1 – Sampling location description**

**Reference locations**

**Fray Jorge – *Baccharis vernalis***

The Altos de Talinay are situated in the northern Cordillera de Talinay, extending north of the Río Limari. It encompasses a portion of the Bosque de Fray Jorge National Park. The precise location of the sample area was not within the boundaries of the national park itself but situated slightly to the north of the park entrance (30°36'04.7" S, 71°41'08.1" W). The population is situated at the northernmost extent of the distribution range of *Baccharis vernalis*.

**Talinay – *Baccharis vernalis***

As with the site in Fray Jorge, the population is situated at the northernmost extent of the distribution range of *Baccharis vernalis*. The Lomas de Los Loros Mountain range (30°45'10.2" S, 71°40'03.4" W) is located to the south of the Río Limari, in close proximity to Cerro Talinay. Collections were made in both the Los Loros valley and on the Los Loros Mountain range.

**Huentelauquén – *Baccharis macraei***

This location (31°36' S, 71° 33' W) represents the northernmost known distribution of *Baccharis macraei*. Large sandy coasts only occur further north from Tongoy onwards. However, the species has not been described in this region (Hellwig 1990). The beach in Huentelauquén is also isolated from *Baccharis linearis*, which makes it a suitable reference point for further study.

**Cavilolén – *Baccharis linearis***

The Cuesta de Cavilolén (31°46' S, 71°19' W) serves as a reference site for *Baccharis linearis* at approximately the same latitude as Los Vilos and Huentelauquén. The site is situated approximately 17 km from the nearest coastline, and therefore is not subject to any influence of *B. macraei*. Additionally, *B. vernalis* was observed at this site. The sample site is located at an altitude of approximately 700 meters and is still accessible by the Camanchaca (coastal fog) from the coast. Consequently, the western slopes exhibit a greater diversity of vegetation than the eastern side.

**Navidad – *Baccharis macraei* & *Baccharis vernalis***

*Baccharis macraei* constitutes an appropriate reference site in Navidad (33°56' S, 71°50' W), situated in a geographically isolated location from *Baccharis linearis*. However, individuals of *B. vernalis* continue to be observed at this site, which could potentially result in the occurrence of hybridization. Nevertheless, due to the disparate flowering periods of the two species, this influence is estimated to be relatively minimal.

**Quinicabén – *Baccharis linearis***

Quinicabén (34°01' S, 71°43' W) is situated at approximately the same latitude as Navidad, the reference site for *Baccharis macraei* and *B. vernalis*. It is located 14 km south-east of Navidad as the crow flies, inland.

**Hualpén – *Baccharis vernalis***

This site is situated on the Hualpén peninsula and represents the southernmost known location of *Baccharis vernalis* in Chile. The plants were discovered on the precipitous coastline (36°48' S, 73°10' W), in close proximity to the "Playa las conchitas" and on the adjoining plateau. This site is geographically distinct from *B. macraei*, which has a distribution range that extends approximately to Pichilemu, and from *B. linearis*, which is more prevalent in inland regions.

**Transects**

**Pichidangui**

The site, situated at 32°07'58.0 "S, 71°30'50.2 "W, is located in the Pichidangui bay and is in close proximity to the mouth of the Río Quilimari. The site is notable for the presence of all species deemed pertinent to the study. Furthermore, an uninterrupted transect was successfully collected between *Baccharis macraei* and *B. linearis*, spanning the transition from beach (sandy soil) to inland (loamy soil).

**Quintay**

The sandy beach of Quintay is encircled by precipitous cliffs. The *Baccharis macraei* population was identified in a location on the sandy beach (33°10'54.3"S, 71°41'02.4"W). The *Baccharis × intermedia* population was identified at an eroded site in the vicinity of a transmission mast (33°11'28.6 "S, 71°41'19.0 "W). The *B. linearis* population was located at a water reservoir (33°11'55.5 "S, 71°39'55.8 "W). This site is an interrupted section, indicating that the individual taxa are separated by buildings or other geographical obstacles. Additionally, *B. vernalis* was identified in a location slightly further south, in the vicinity of Tunquen.

**Topocalma**

Individuals of *Baccharis macraei* and *B. vernalis* were observed at the black sand beach in Topocalma (34°06'12.1 "S, 71°58'32.6 "W). Additionally, specimens of potential hybrids and *B. linearis* were collected at the roadside leading to the beach (34°05'30.2 "S, 71°56'50.6 "W). Another site of *B. linearis* was identified at the outset of Cam. a Puertecillo (34°05'05.7 "S, 71°55'31.8 "W).

**Pichilemu**

The transect is located at Playa La Pichita (34°26'30.3 "S, 72°02'22.9 "W). *Baccharis macraei, B. vernalis* and the hybrid B*. × intermedia* were found there, but no individuals of *B. linearis*. The latter was first encountered further east along the road (34°28'07.5 "S. 72°01'06.3 "W) and again further south of the Río Palmilla (34°30'42.0 "S, 72°00'19.5 "W).

**Appendix S2 – LGC Library preparation protocol and results from GBS analysis**

**I. Restriction digest:**

20-500 ng of genomic DNA were digested with 2 Units each ApekI and PstI-HF(NEB) in 1 times NEBuffer 3.1 in 20μl volume for 30 min at 37°C. The restriction enzymes were heat inactivated by incubation at 75°C for 30 min.

**II. nGBS library construction:**

**a) Ligation Reaction**

10 μl of each restriction digest were transferred to a new 96-well PCR plate, mixed on ice first with 1.5 μl of one of 96 inline-barcoded forward PstI Adaptors (pre-hybridized, concentration 1 pM/μl), followed by addition of 20μl Ligation master mix (contains: 15 μl NEB Quick ligation buffer, 0.4 μl NEB Quick Ligase, 5 pM pre-hybridized common reverse ApekI Adaptors). Ligation reactions were incubated for 1h at RT, followed by heat inactivation for 10 min at 65°C.

**b) Library purification**

all reactions were diluted with 30 μl TE 10/50 (10mM Tris/HCl, 50mM EDTA, pH:8.0) and mixed with 50 μl Agencourt XP beads, incubated for 10 min at RT and placed for 5 min on a magnet to collect the beads. The supernatant was discarded, and the beads were washed two times with 200 μl 80% Ethanol. Beads were air dried for 10 min and libraries were eluted in 15 μl Tris Buffer (10 mM Tris/HCl pH:9).

**c) Library amplification**

10 μl of each of the 96 Libraries were separately amplified in 20 μl PCR reactions using MyTaq (Bioline) and standard Illumina TrueSeq amplification primers. Cycle number was limited to 16 Cycles.

**III. Pooling and clean-up of ddRAD libraries:**

5 μl from each of the 96 amplified libraries were pooled. PCR primer and small amplicons were removed by Agencourt XP bead purification using 0.8 Volume of beads. The PCR enzyme was removed by an additional purification on Qiagen MinElute Columns. The pooled library was eluted in a final volume of 20μl Tris Buffer (5 mM Tris/HCl pH:9).

**IV. Normalisation**

Normalisation was done using Trimmer Kit (Evrogen). 1 μg pooled GBS library in 12 μl was mixed with 4 μl 4x hybridization buffer, denatured for 3 min at 98°C and incubated for 3 hours at 68°C to allow reassociation of DNA fragments. 20 μl of 2x DSN master buffer was added, and the samples were incubated for 10 min at 66°C. One Unit of DSN enzyme (1 U/μl) was added and the reaction was incubated for another 40 min. Reaction was terminated by the addition of 20 μl DSN Stop Solution, purified on a Qiagen MinElute Column and eluted in 10 μl Tris Buffer (5 mM Tris/HCl pH:9).

**V. Reamplification**

The normalized library pool was reamplified in 100μl PCR reactions using MyTaq (Bioline). An i5-Adaptor primer was used to include an i5-Index into the library, allowing parallel sequencing of multiple libraries on the Illumina NextSeq 500/550 sequencer. Cycle number was limited to 14 cycles.

**GBS – Results**

**Table S1:** Results from GBS analysis for the data set including Baccharis linearis, B. macraei, and B. × intermedia: (group: all samples), mapping the genetic data against the reference created in (Schneider & Hellwig 2024).

| Cluster locus count: | 63,348 |
| --- | --- |
| Mapping rate: | 74.3 % |
| Polymorphic locus count: | 30,773 |
| Total number of SNPs across all samples: | 120,064 |
| Total number of SNPs across all samples with min. read count of 8: | 99,995 |
| Total number of fully covered SNPs in 66 % of the samples, with allele frequency at or above 5 % and min. read count of 8: | 4,463 |
| Total number of fully covered SNPs in 66 % of the samples, with allele frequency at or above 10 % and min. read count of 8: | 3,182 |

**Appendix S3 – PCA and Clustering**

**Figure S1:** Cross entropy plots of the genetic clusters from four Baccharis species in Chile. The dotted line indicates the number of clusters (K = 4, 7 and 12) investigated further in this thesis. A) minimal cross entropy for the clusters: K = 0 – 20, B) Mean cross entropy for the clusters: K = 0 – 20 with standard error as bars.


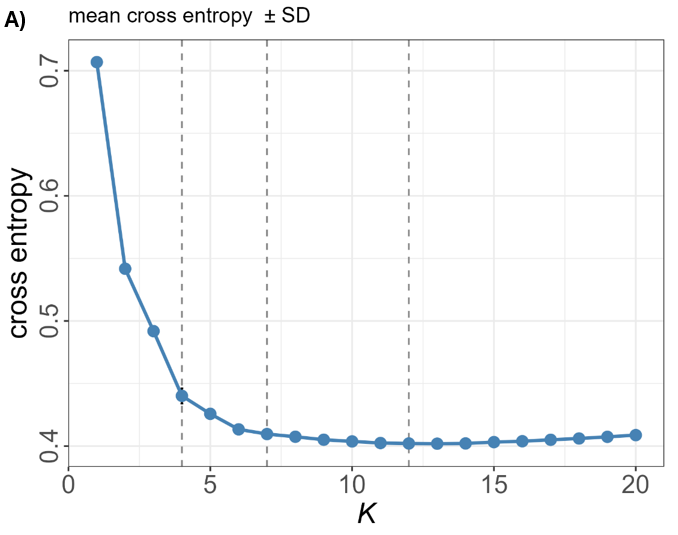

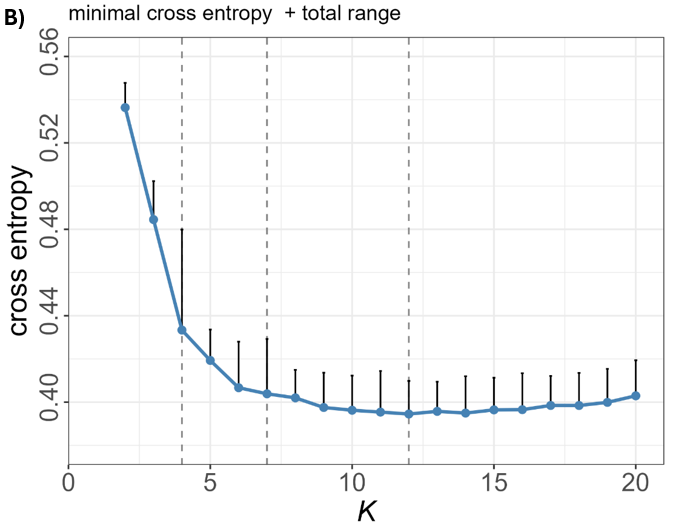


**Figure S2:** Genetic cluster assignment of the investigated *Baccharis* taxa in Chile shown as bar plot. The figure shows the results for four clusters corresponding to the four taxa sampled in the field: *Baccharis linearis, B. × intermedia, B. macraei* and *B. vernalis*, which are indicated by different colours. The samples are ordered by preliminary field determination. The localities are arranged from left to right in an N-S sequence.


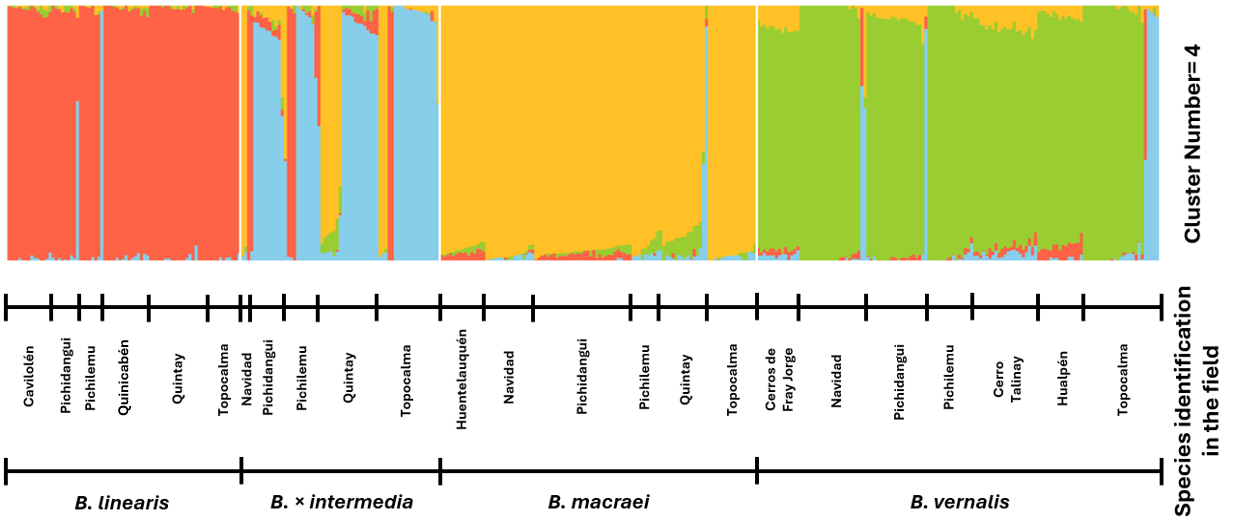


**Table S1:** Principal components, standard deviation and proportion of variance from the PCA performed with the SNP’s from Baccharis.

| **Principal Component** | **Standard**  **deviation** | **Proportion of Variance** | **Cumulative Proportion** |
| --- | --- | --- | --- |
| PC 1 | 25.39 | 0.372 | 0.253 |
| PC 2 | 13.94 | 0.112 | 0.393 |
| PC 3 | 8.01 | 0.037 | 0. 473 |
| PC 4 | 6.73 | 0.026 | 0.541 |
| PC 5 | 4.61 | 0.012 | 0.586 |
| PC 6 | 4.34 | 0.007 | 0.630 |
| PC 7 | 3.54 | 0.007 | 0.665 |
| PC 8 | 3.50 | 0.006 | 0.704 |
| PC 9 | 3.26 | 0.005 | 0.733 |
| PC 10 | 3.05 | 0.005 | 0.764 |

**Figure S3:** PCA results for Baccharis samples from Chile. A) Shown are axis PC 1 (25.39 %) and PC 2 (13.94 %) and B) PC 1 (25.39 %) and PC 3 (8.01 %). Assignment of individuals is done according to the sNMF using K = 4 clusters. Individuals with more than 25 % belonging to another group in the sNMF clustering were grouped into the "not assignable" category.


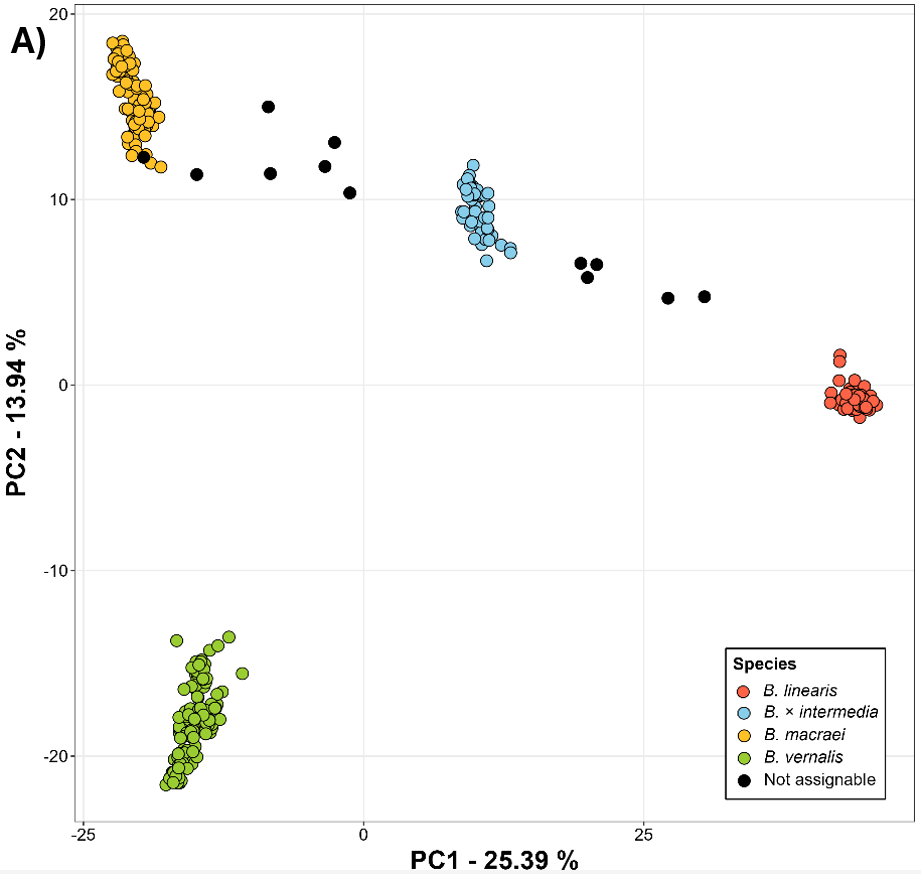


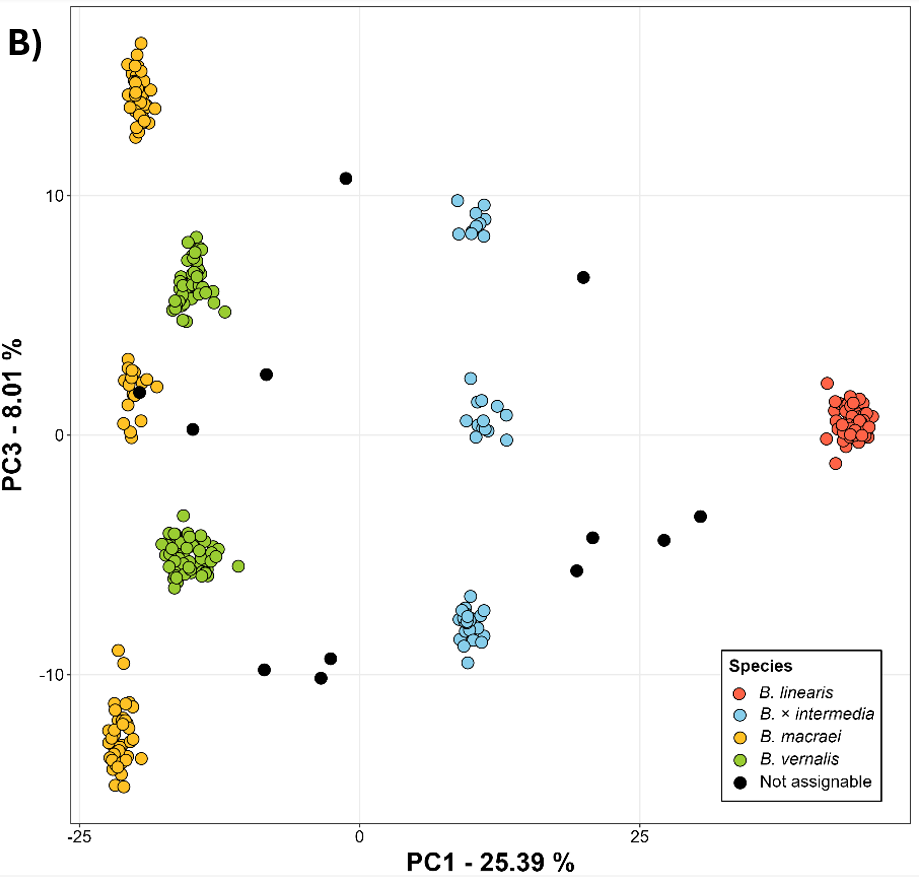


**Appendix S4 – Percentage of heterozygous SNPs**

**Figure S1**: Violin plots of the heterozygosity. Groups resulting from the clustering (K=4). *Baccharis × intermedia* has a higher heterozygosity value compared to all other taxa.


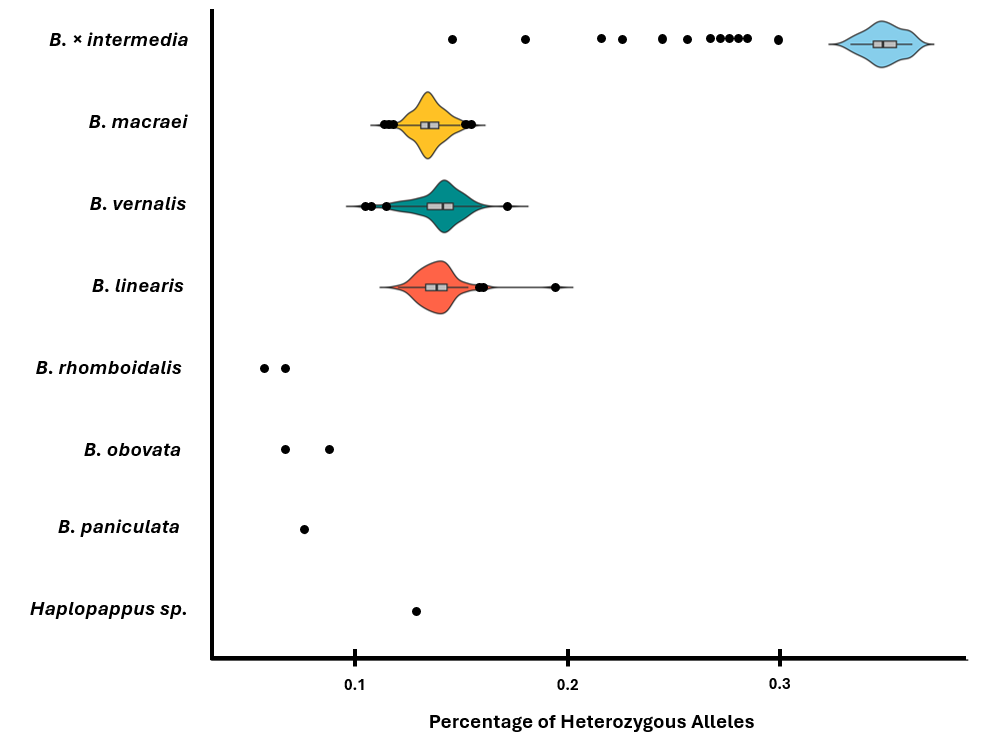


**Figure S2**: Violin plots of the heterozygosity. Groups resulting from the clustering (K=7). *Baccharis × intermedia* has a higher heterozygosity value compared to all other taxa.


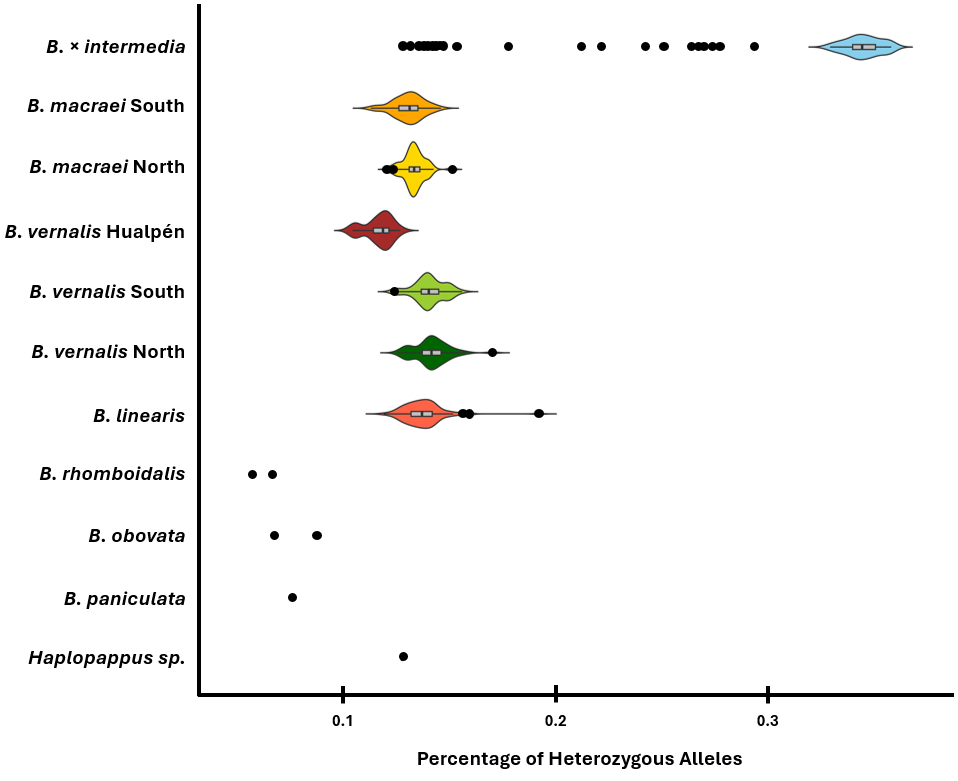


**Figure S3**: Violin plots of the heterozygosity. Groups resulting from the clustering (K=12). *Baccharis × intermedia* has a higher heterozygosity value compared to all other taxa.


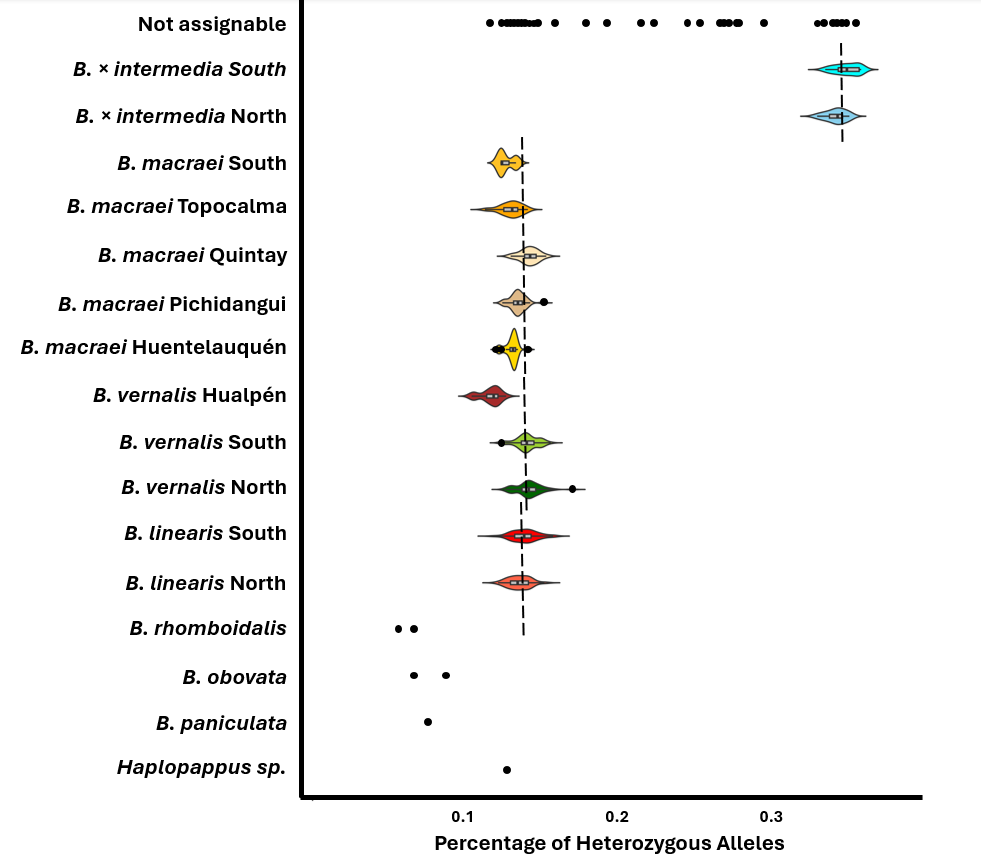


**Figure S4:** Violin plot of percentage of heterozygous SNPs by species and location. *Baccharis × intermedia* has a higher heterozygosity value compared to all other taxa.


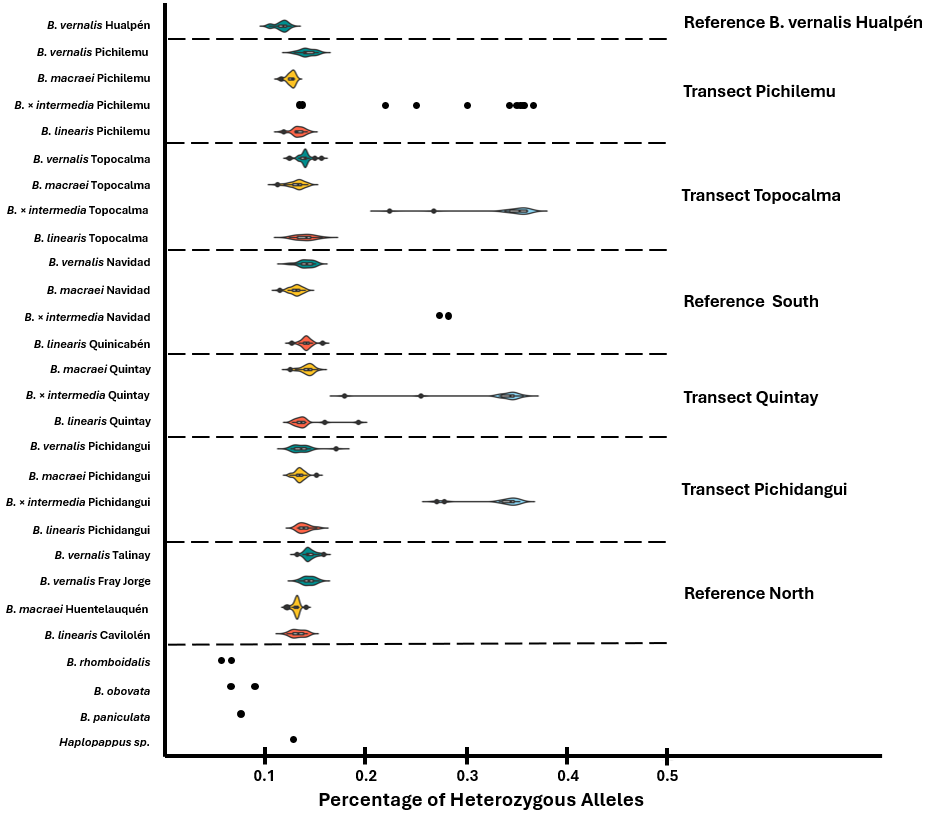


**Appendix S5 – Nei-F_ST_**

**Table S1:** Genetic distances (Nei-F_ST_) between *Baccharis* species in Chile. Groups are resulting from the clustering (K=4).

|  | ***B. × intermedia*** | ***B. linearis*** | ***B. macraei*** | ***B. vernalis*** |
| --- | --- | --- | --- | --- |
| ***B. × intermedia*** | NA | 0.108 | 0.079 | 0.093 |
| ***B. linearis*** | 0.108 | NA | 0.159 | 0.144 |
| ***B. macraei*** | 0.079 | 0.159 | NA | 0.048 |
| ***B. vernalis*** | 0.093 | 0.144 | 0.048 | NA |

**Table S2:** Genetic distances (Nei-F_ST_) between *Baccharis* species in Chile. Groups are resulting from the clustering (K=7).

|  | ***B. × intermedia*** | ***B. linearis*** | ***B. macraei* North** | ***B. macraei* South** | ***B. vernalis* North** | ***B. vernalis* South** | ***B. vernalis* Hualpén** |
| --- | --- | --- | --- | --- | --- | --- | --- |
| ***B. × intermedia*** | NA | 0.108 | 0.088 | 0.088 | 0.095 | 0.106 | 0.114 |
| ***B. linearis*** | 0.108 | NA | 0.165 | 0.172 | 0.149 | 0.158 | 0.161 |
| ***B. macraei* North** | 0.088 | 0.165 | NA | 0.036 | 0.057 | 0.073 | 0.085 |
| ***B. macraei* South** | 0.088 | 0.172 | 0.036 | NA | 0.062 | 0.072 | 0.084 |
| ***B. vernalis* North** | 0.095 | 0.149 | 0.057 | 0.062 | NA | 0.032 | 0.048 |
| ***B. vernalis* South** | 0.106 | 0.158 | 0.073 | 0.072 | 0.032 | NA | 0.032 |
| ***B. vernalis***  **Hualpén** | 0.114 | 0.161 | 0.085 | 0.084 | 0.048 | 0.032 | NA |

**Table S3:** Genetic distances (Nei-F_ST_) between Baccharis species in Chile. Groups are resulting from the clustering (K=12).

|  | ***B. × intermedia***  **North** | ***B. × intermedia***  **South** | ***B. linearis***  **North** | ***B. linearis***  **South** | ***B. macraei* Huentelauquén** | ***B. macraei* Pichidangui** | ***B. macraei***  **Quintay** | ***B. macraei***  **South** | ***B. macraei***  **Topocalma** | ***B. vernalis* North** | ***B. vernalis* South** | ***B. vernalis* Hualpén** |
| --- | --- | --- | --- | --- | --- | --- | --- | --- | --- | --- | --- | --- |
| ***B. × intermedia***  **North** | NA | 0.026 | 0.1151 | 0.1173 | 0.0949 | 0.0851 | 0.0951 | 0.1229 | 0.107 | 0.1013 | 0.1143 | 0.1235 |
| ***B. × intermedia***  **South** | 0.026 | NA | 0.12 | 0.1172 | 0.1043 | 0.0994 | 0.0933 | 0.1033 | 0.0892 | 0.1014 | 0.1117 | 0.1201 |
| ***B. linearis***  **North** | 0.1151 | 0.12 | NA | 0.01 | 0.1734 | 0.1693 | 0.1683 | 0.1935 | 0.1766 | 0.1507 | 0.1608 | 0.1639 |
| ***B. linearis***  **South** | 0.1173 | 0.1172 | 0.01 | NA | 0.1727 | 0.1689 | 0.1678 | 0.1923 | 0.1752 | 0.1498 | 0.1582 | 0.162 |
| ***B. macraei* Huentelauquén** | 0.0949 | 0.1043 | 0.1734 | 0.1727 | NA | 0.0187 | 0.0327 | 0.0711 | 0.0463 | 0.0633 | 0.0792 | 0.0922 |
| ***B. macraei* Pichidangui** | 0.0851 | 0.0994 | 0.1693 | 0.1689 | 0.0187 | NA | 0.0266 | 0.0631 | 0.0412 | 0.059 | 0.075 | 0.0872 |
| ***B. macraei***  **Quintay** | 0.0951 | 0.0933 | 0.1683 | 0.1678 | 0.0327 | 0.0266 | NA | 0.0512 | 0.0289 | 0.0551 | 0.0687 | 0.0809 |
| ***B. macraei***  **South** | 0.1229 | 0.1033 | 0.1935 | 0.1923 | 0.0711 | 0.0631 | 0.0512 | NA | 0.0368 | 0.0799 | 0.0881 | 0.1043 |
| ***B. macraei***  **Topocalma** | 0.107 | 0.0892 | 0.1766 | 0.1752 | 0.0463 | 0.0412 | 0.0289 | 0.0368 | NA | 0.0639 | 0.0735 | 0.0856 |
| ***B. vernalis* North** | 0.1013 | 0.1014 | 0.1507 | 0.1498 | 0.0633 | 0.059 | 0.0551 | 0.0799 | 0.0639 | NA | 0.0315 | 0.0479 |
| ***B. vernalis* South** | 0.1143 | 0.1117 | 0.1608 | 0.1582 | 0.0792 | 0.075 | 0.0687 | 0.0881 | 0.0735 | 0.0315 | NA | 0.0319 |
| ***B. vernalis***  **Hualpén** | 0.1235 | 0.1201 | 0.1639 | 0.162 | 0.0922 | 0.0872 | 0.0809 | 0.1043 | 0.0856 | 0.0479 | 0.0319 | NA |

**Table S4:** Pairwise Nei-F_ST_ between Baccharis species in Chile analysed for all populations.

|  | ***B.× intermedia*** | | | | | ***B. linearis*** | | | | | | ***B. macraei*** | | | | | |
| --- | --- | --- | --- | --- | --- | --- | --- | --- | --- | --- | --- | --- | --- | --- | --- | --- | --- |
|  | Navidad | Pichi-dangui | Pichi-lemu | Quintay | Topo-calma | Cavi-lolén | Pichi-dangui | Pichi-lemu | Quini-cabén | Quintay | Topo-calma | Huentel-auquén | Navidad | Pichi-dangui | Pichi-lemu | Quintay | Topo-calma |
| ***B.×-intermedia*** |  |  |  |  |  |  |  |  |  |  |  |  |  |  |  |  |  |
| Navidad | NA | 0.033 | 0.006 | 0.021 | 0.016 | 0.119 | 0.114 | 0.112 | 0.114 | 0.115 | 0.113 | 0.100 | 0.086 | 0.093 | 0.081 | 0.072 | 0.086 |
| Pichidangui | 0.033 | NA | 0.025 | 0.016 | 0.024 | 0.112 | 0.105 | 0.112 | 0.113 | 0.112 | 0.112 | 0.090 | 0.105 | 0.081 | 0.098 | 0.083 | 0.105 |
| Pichilemu | 0.006 | 0.025 | NA | 0.013 | 0.007 | 0.090 | 0.085 | 0.084 | 0.086 | 0.087 | 0.085 | 0.085 | 0.073 | 0.081 | 0.062 | 0.064 | 0.073 |
| Quintay | 0.021 | 0.016 | 0.013 | NA | 0.013 | 0.109 | 0.105 | 0.107 | 0.109 | 0.107 | 0.108 | 0.087 | 0.088 | 0.084 | 0.081 | 0.064 | 0.088 |
| Topocalma | 0.016 | 0.024 | 0.007 | 0.013 | NA | 0.113 | 0.110 | 0.108 | 0.110 | 0.111 | 0.109 | 0.100 | 0.087 | 0.097 | 0.083 | 0.081 | 0.087 |
| ***B. linearis*** |  |  |  |  |  |  |  |  |  |  |  |  |  |  |  |  |  |
| Cavilolén | 0.119 | 0.112 | 0.090 | 0.109 | 0.113 | NA | 0.005 | 0.012 | 0.013 | 0.007 | 0.010 | 0.175 | 0.178 | 0.166 | 0.180 | 0.152 | 0.176 |
| Pichidangui | 0.114 | 0.105 | 0.085 | 0.105 | 0.110 | 0.005 | NA | 0.013 | 0.012 | 0.008 | 0.011 | 0.172 | 0.175 | 0.163 | 0.177 | 0.149 | 0.174 |
| Pichilemu | 0.112 | 0.112 | 0.084 | 0.107 | 0.108 | 0.012 | 0.013 | NA | 0.003 | 0.007 | 0.002 | 0.173 | 0.175 | 0.164 | 0.177 | 0.150 | 0.174 |
| Quinicabén | 0.114 | 0.113 | 0.086 | 0.109 | 0.110 | 0.013 | 0.012 | 0.003 | NA | 0.007 | 0.003 | 0.174 | 0.177 | 0.166 | 0.179 | 0.152 | 0.175 |
| Quintay | 0.115 | 0.112 | 0.087 | 0.107 | 0.111 | 0.007 | 0.008 | 0.007 | 0.007 | NA | 0.006 | 0.174 | 0.177 | 0.165 | 0.179 | 0.151 | 0.175 |
| Topocalma | 0.113 | 0.112 | 0.085 | 0.108 | 0.109 | 0.010 | 0.011 | 0.002 | 0.003 | 0.006 | NA | 0.173 | 0.176 | 0.165 | 0.178 | 0.151 | 0.174 |
| ***B. macraei*** |  |  |  |  |  |  |  |  |  |  |  |  |  |  |  |  |  |
| Huentel-auquén | 0.100 | 0.090 | 0.085 | 0.087 | 0.100 | 0.175 | 0.172 | 0.173 | 0.174 | 0.174 | 0.173 | NA | 0.049 | 0.019 | 0.046 | 0.030 | NA |
| Navidad | 0.086 | 0.105 | 0.073 | 0.088 | 0.087 | 0.178 | 0.175 | 0.175 | 0.177 | 0.177 | 0.176 | 0.049 | NA | 0.044 | 0.012 | 0.027 | 0.049 |
| Pichidangui | 0.093 | 0.081 | 0.081 | 0.084 | 0.097 | 0.166 | 0.163 | 0.164 | 0.166 | 0.165 | 0.165 | 0.019 | 0.044 | NA | 0.039 | 0.025 | 0.019 |
| Pichilemu | 0.081 | 0.098 | 0.062 | 0.081 | 0.083 | 0.180 | 0.177 | 0.177 | 0.179 | 0.179 | 0.178 | 0.046 | 0.012 | 0.039 | NA | 0.021 | 0.046 |
| Quintay | 0.072 | 0.083 | 0.064 | 0.064 | 0.081 | 0.152 | 0.149 | 0.150 | 0.152 | 0.151 | 0.151 | 0.030 | 0.027 | 0.025 | 0.021 | NA | 0.030 |
| Topocalma | 0.082 | 0.102 | 0.070 | 0.085 | 0.084 | 0.176 | 0.174 | 0.174 | 0.175 | 0.175 | 0.174 | 0.045 | 0.006 | 0.039 | 0.005 | 0.023 | 0.045 |

**Table S5:** Pairwise Nei–F_ST_ between Baccharis species in Chile analysed for all populations (continued).

|  | ***B.× intermedia*** | | | | | ***B. linearis*** | | | | | | ***B. macraei*** | | | | | |
| --- | --- | --- | --- | --- | --- | --- | --- | --- | --- | --- | --- | --- | --- | --- | --- | --- | --- |
|  | Navidad | Pichi-dangui | Pichi-lemu | Quintay | Topo-calma | Cavi-lolén | Pichi-dangui | Pichi-lemu | Quini-cabén | Quintay | Topo-calma | Huentel-auquén | Navidad | Pichi-dangui | Pichi-lemu | Quintay | Topo-calma |
| ***B. vernalis*** |  |  |  |  |  |  |  |  |  |  |  |  |  |  |  |  |  |
| Fray Jorge | 0.096 | 0.100 | 0.084 | 0.090 | 0.101 | 0.157 | 0.154 | 0.155 | 0.157 | 0.157 | 0.156 | 0.068 | 0.071 | 0.064 | 0.067 | 0.056 | 0.069 |
| Navidad | 0.101 | 0.110 | 0.090 | 0.099 | 0.108 | 0.161 | 0.158 | 0.158 | 0.159 | 0.161 | 0.159 | 0.079 | 0.073 | 0.074 | 0.069 | 0.063 | 0.073 |
| Pichidangui | 0.103 | 0.107 | 0.089 | 0.096 | 0.107 | 0.161 | 0.159 | 0.159 | 0.160 | 0.161 | 0.159 | 0.072 | 0.074 | 0.067 | 0.068 | 0.058 | 0.071 |
| Pichilemu | 0.104 | 0.113 | 0.093 | 0.103 | 0.112 | 0.165 | 0.159 | 0.160 | 0.161 | 0.163 | 0.162 | 0.086 | 0.080 | 0.082 | 0.078 | 0.072 | 0.082 |
| Talinay | 0.090 | 0.097 | 0.080 | 0.087 | 0.098 | 0.149 | 0.147 | 0.147 | 0.148 | 0.149 | 0.148 | 0.065 | 0.068 | 0.061 | 0.063 | 0.053 | 0.066 |
| Hualpén | 0.112 | 0.118 | 0.097 | 0.107 | 0.116 | 0.165 | 0.162 | 0.163 | 0.163 | 0.164 | 0.163 | 0.092 | 0.086 | 0.087 | 0.085 | 0.075 | 0.087 |
| Topocalma | 0.101 | 0.110 | 0.090 | 0.100 | 0.109 | 0.161 | 0.157 | 0.157 | 0.158 | 0.160 | 0.159 | 0.079 | 0.074 | 0.075 | 0.072 | 0.065 | 0.075 |

**Table S6:** Pairwise Nei–F_ST_ between Baccharis species in Chile analysed for all populations (continued).

|  | ***B. vernalis*** | | | | | | |
| --- | --- | --- | --- | --- | --- | --- | --- |
|  | Fray Jorge | Navidad | Pichidangui | Pichilemu | Talinay | Hualpén | Topocalma |
| ***B.× intermedia*** |  |  |  |  |  |  |  |
| Navidad | 0.096 | 0.101 | 0.103 | 0.104 | 0.090 | 0.112 | 0.101 |
| Pichidangui | 0.100 | 0.110 | 0.107 | 0.113 | 0.097 | 0.118 | 0.110 |
| Pichilemu | 0.084 | 0.090 | 0.089 | 0.093 | 0.080 | 0.097 | 0.090 |
| Quintay | 0.090 | 0.099 | 0.096 | 0.103 | 0.087 | 0.107 | 0.100 |
| Topocalma | 0.101 | 0.108 | 0.107 | 0.112 | 0.098 | 0.116 | 0.109 |
| ***B. linearis*** |  |  |  |  |  |  |  |
| Cavilolén | 0.157 | 0.161 | 0.161 | 0.165 | 0.149 | 0.165 | 0.161 |
| Pichidangui | 0.154 | 0.158 | 0.159 | 0.159 | 0.147 | 0.162 | 0.157 |
| Pichilemu | 0.155 | 0.158 | 0.159 | 0.160 | 0.147 | 0.163 | 0.157 |
| Quinicabén | 0.157 | 0.159 | 0.160 | 0.161 | 0.148 | 0.163 | 0.158 |
| Quintay | 0.157 | 0.161 | 0.161 | 0.163 | 0.149 | 0.164 | 0.160 |
| Topocalma | 0.156 | 0.159 | 0.159 | 0.162 | 0.148 | 0.163 | 0.159 |
| ***B. macraei*** |  |  |  |  |  |  |  |
| Huentelauquén | 0.068 | 0.079 | 0.072 | 0.086 | 0.065 | 0.092 | 0.079 |
| Navidad | 0.071 | 0.073 | 0.074 | 0.080 | 0.068 | 0.086 | 0.074 |
| Pichidangui | 0.064 | 0.074 | 0.067 | 0.082 | 0.061 | 0.087 | 0.075 |
| Pichilemu | 0.067 | 0.069 | 0.068 | 0.078 | 0.063 | 0.085 | 0.072 |
| Quintay | 0.056 | 0.063 | 0.058 | 0.071 | 0.053 | 0.075 | 0.065 |
| Topocalma | 0.069 | 0.073 | 0.071 | 0.082 | 0.066 | 0.087 | 0.075 |
| ***B. vernalis*** |  |  |  |  |  |  |  |
| Fray Jorge | NA | 0.038 | 0.020 | 0.047 | 0.007 | 0.055 | 0.039 |
| Navidad | 0.038 | NA | 0.035 | 0.008 | 0.033 | 0.034 | 0.002 |
| Pichidangui | 0.020 | 0.035 | NA | 0.045 | 0.018 | 0.056 | 0.037 |
| Pichilemu | 0.047 | 0.008 | 0.045 | NA | 0.041 | 0.035 | 0.005 |
| Talinay | 0.007 | 0.033 | 0.018 | 0.041 | NA | 0.049 | 0.034 |
| Hualpén | 0.055 | 0.034 | 0.056 | 0.035 | 0.049 | NA | 0.032 |
| Topocalma | 0.039 | 0.002 | 0.037 | 0.005 | 0.034 | 0.032 | NA |

**Appendix S6 – Isolation by distance**

**Figure S1:** Linear regressions plot between geographical distance and Nei-F_ST_, using the "Pearson" correlation method, showing also the results of the Mantel test (r and p-value) A) For all populations of Baccharis linearis, B) For all populations of B. macraei, C) For all populations of B. vernalis.

**
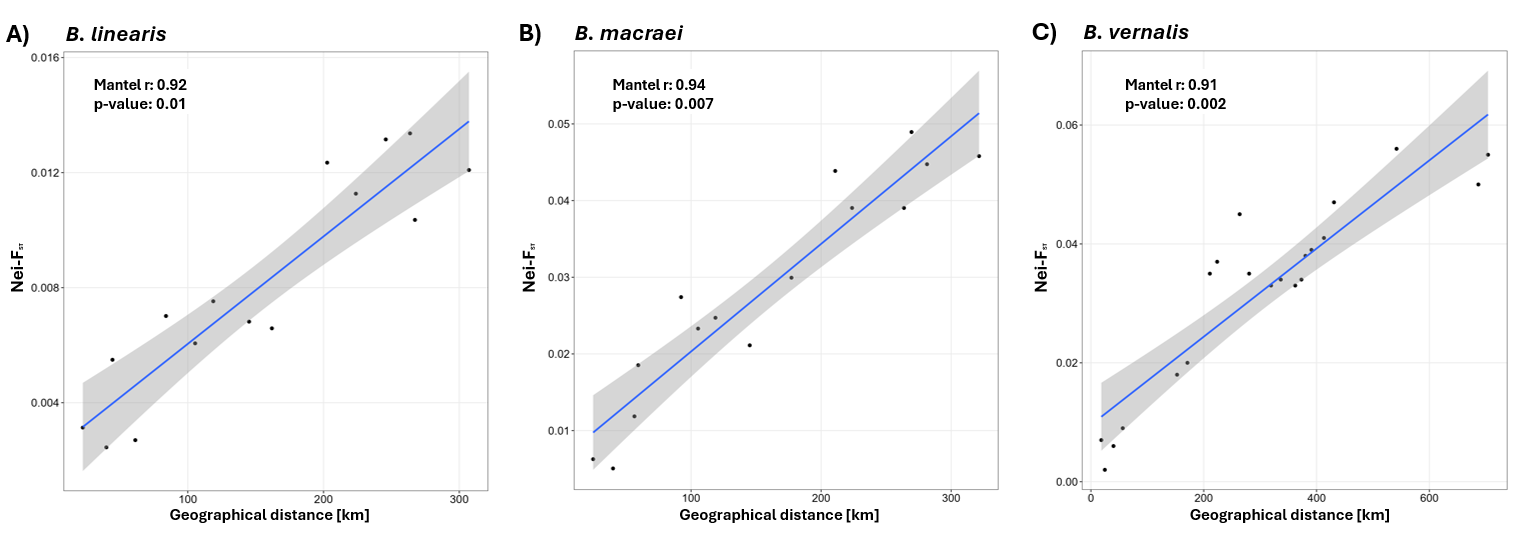
**

**Table S1:** Geographical distances between each population of Baccharis in kilometres measured with spherical geometry.

|  | ***B.× intermedia*** | | | | | ***B. linearis*** | | | | | | ***B. macraei*** | | | | | |
| --- | --- | --- | --- | --- | --- | --- | --- | --- | --- | --- | --- | --- | --- | --- | --- | --- | --- |
|  | Navidad | Pichi-dangui | Pichi-lemu | Quintay | Topo-calma | Cavi-lolén | Pichi-dangui | Pichi-lemu | Quini-cabe | Quintay | Topo-calma | Huentel-auquén | Navidad | Pichi-dangui | Pichi-lemu | Quintay | Topo-calma |
| ***B.×-intermedia*** |  |  |  |  |  |  |  |  |  |  |  |  |  |  |  |  |  |
| Navidad | 0.00 | 210.87 | 56.47 | 92.35 | 24.74 | 253.39 | 210.87 | 56.47 | 14.56 | 92.35 | 24.74 | 269.54 | 0.00 | 210.87 | 56.47 | 92.35 | 24.74 |
| Pichidangui | 210.87 | 0.00 | 263.73 | 118.77 | 223.82 | 44.54 | 0.00 | 263.73 | 202.68 | 118.77 | 223.82 | 59.41 | 210.87 | 0.00 | 263.73 | 118.77 | 223.82 |
| Pichilemu | 56.47 | 263.73 | 0.00 | 145.14 | 40.03 | 307.11 | 263.73 | 0.00 | 61.35 | 145.14 | 40.03 | 321.47 | 56.47 | 263.73 | 0.00 | 145.14 | 40.03 |
| Quintay | 92.35 | 118.77 | 145.14 | 0.00 | 105.42 | 161.96 | 118.77 | 145.14 | 83.94 | 0.00 | 105.42 | 177.21 | 92.35 | 118.77 | 145.14 | 0.00 | 105.42 |
| Topocalma | 24.74 | 223.82 | 40.03 | 105.42 | 0.00 | 267.34 | 223.82 | 40.03 | 22.70 | 105.42 | 0.00 | 281.45 | 24.74 | 223.82 | 40.03 | 105.42 | 0.00 |
| ***B. linearis*** |  |  |  |  |  |  |  |  |  |  |  |  |  |  |  |  |  |
| Cavilolén | 253.39 | 44.54 | 307.11 | 161.96 | 267.34 | 0.00 | 44.54 | 307.11 | 245.86 | 161.96 | 267.34 | 29.95 | 253.39 | 44.54 | 307.11 | 161.96 | 267.34 |
| Pichidangui | 210.87 | 0.00 | 263.73 | 118.77 | 223.82 | 44.54 | 0.00 | 263.73 | 202.68 | 118.77 | 223.82 | 59.41 | 210.87 | 0.00 | 263.73 | 118.77 | 223.82 |
| Pichilemu | 56.47 | 263.73 | 0.00 | 145.14 | 40.03 | 307.11 | 263.73 | 0.00 | 61.35 | 145.14 | 40.03 | 321.47 | 56.47 | 263.73 | 0.00 | 145.14 | 40.03 |
| Quinicabén | 14.56 | 202.68 | 61.35 | 83.94 | 22.70 | 245.86 | 202.68 | 61.35 | 0.00 | 83.94 | 22.70 | 260.80 | 14.56 | 202.68 | 61.35 | 83.94 | 22.70 |
| Quintay | 92.35 | 118.77 | 145.14 | 0.00 | 105.42 | 161.96 | 118.77 | 145.14 | 83.94 | 0.00 | 105.42 | 177.21 | 92.35 | 118.77 | 145.14 | 0.00 | 105.42 |
| Topocalma | 24.74 | 223.82 | 40.03 | 105.42 | 0.00 | 267.34 | 223.82 | 40.03 | 22.70 | 105.42 | 0.00 | 281.45 | 24.74 | 223.82 | 40.03 | 105.42 | 0.00 |
| ***B. macraei*** |  |  |  |  |  |  |  |  |  |  |  |  |  |  |  |  |  |
| Huentel-auquén | 269.54 | 59.41 | 321.47 | 177.21 | 281.45 | 29.95 | 59.41 | 321.47 | 260.80 | 177.21 | 281.45 | 0.00 | 269.54 | 59.41 | 321.47 | 177.21 | 281.45 |
| Navidad | 0.00 | 210.87 | 56.47 | 92.35 | 24.74 | 253.39 | 210.87 | 56.47 | 14.56 | 92.35 | 24.74 | 269.54 | 0.00 | 210.87 | 56.47 | 92.35 | 24.74 |
| Pichidangui | 210.87 | 0.00 | 263.73 | 118.77 | 223.82 | 44.54 | 0.00 | 263.73 | 202.68 | 118.77 | 223.82 | 59.41 | 210.87 | 0.00 | 263.73 | 118.77 | 223.82 |
| Pichilemu | 56.47 | 263.73 | 0.00 | 145.14 | 40.03 | 307.11 | 263.73 | 0.00 | 61.35 | 145.14 | 40.03 | 321.47 | 56.47 | 263.73 | 0.00 | 145.14 | 40.03 |
| Quintay | 92.35 | 118.77 | 145.14 | 0.00 | 105.42 | 161.96 | 118.77 | 145.14 | 83.94 | 0.00 | 105.42 | 177.21 | 92.35 | 118.77 | 145.14 | 0.00 | 105.42 |
| Topocalma | 24.74 | 223.82 | 40.03 | 105.42 | 0.00 | 267.34 | 223.82 | 40.03 | 22.70 | 105.42 | 0.00 | 281.45 | 24.74 | 223.82 | 40.03 | 105.42 | 0.00 |

**Table S2:** Geographical distances between each population of Baccharis in kilometres measured with spherical geometry (continued).

|  | ***B.× intermedia*** | | | | | ***B. linearis*** | | | | | | ***B. macraei*** | | | | | |
| --- | --- | --- | --- | --- | --- | --- | --- | --- | --- | --- | --- | --- | --- | --- | --- | --- | --- |
|  | Navidad | Pichi-dangui | Pichi-lemu | Quintay | Topo-calma | Cavi-lolén | Pichi-dangui | Pichi-lemu | Quini-cabén | Quintay | Topo-calma | Huentel-auquén | Navidad | Pichi-dangui | Pichi-lemu | Quintay | Topo-calma |
| ***B. vernalis*** |  |  |  |  |  |  |  |  |  |  |  |  |  |  |  |  |  |
| Fray Jorge | 380.26 | 171.10 | 430.94 | 287.97 | 390.93 | 134.35 | 171.10 | 430.94 | 370.93 | 287.97 | 390.93 | 111.70 | 380.26 | 171.10 | 430.94 | 287.97 | 390.93 |
| Navidad | 0.00 | 210.87 | 56.47 | 92.35 | 24.74 | 253.39 | 210.87 | 56.47 | 14.56 | 92.35 | 24.74 | 269.54 | 0.00 | 210.87 | 56.47 | 92.35 | 24.74 |
| Pichidangui | 210.87 | 0.00 | 263.73 | 118.77 | 223.82 | 44.54 | 0.00 | 263.73 | 202.68 | 118.77 | 223.82 | 59.41 | 210.87 | 0.00 | 263.73 | 118.77 | 223.82 |
| Pichilemu | 56.47 | 263.73 | 0.00 | 145.14 | 40.03 | 307.11 | 263.73 | 0.00 | 61.35 | 145.14 | 40.03 | 321.47 | 56.47 | 263.73 | 0.00 | 145.14 | 40.03 |
| Talinay | 362.18 | 152.73 | 413.12 | 269.86 | 373.11 | 115.93 | 152.73 | 413.12 | 352.97 | 269.86 | 373.11 | 93.32 | 362.18 | 152.73 | 413.12 | 269.86 | 373.11 |
| Hualpén | 336.58 | 541.80 | 280.51 | 424.49 | 319.35 | 585.87 | 541.80 | 280.51 | 341.59 | 424.49 | 319.35 | 597.75 | 336.58 | 541.80 | 280.51 | 424.49 | 319.35 |
| Topocalma | 24.74 | 223.82 | 40.03 | 105.42 | 0.00 | 267.34 | 223.82 | 40.03 | 22.70 | 105.42 | 0.00 | 281.45 | 24.74 | 223.82 | 40.03 | 105.42 | 0.00 |

**Table S3:** Geographical distances between each population of Baccharis in kilometres measured with spherical geometry (continued).

|  | ***B. vernalis*** | | | | | | |
| --- | --- | --- | --- | --- | --- | --- | --- |
|  | Fray Jorge | Navidad | Pichidangui | Pichilemu | Talinay | Hualpén | Topocalma |
| ***B.× intermedia*** |  |  |  |  |  |  |  |
| Navidad | 380.26 | 0.00 | 210.87 | 56.47 | 362.18 | 336.58 | 24.74 |
| Pichidangui | 171.10 | 210.87 | 0.00 | 263.73 | 152.73 | 541.80 | 223.82 |
| Pichilemu | 430.94 | 56.47 | 263.73 | 0.00 | 413.12 | 280.51 | 40.03 |
| Quintay | 287.97 | 92.35 | 118.77 | 145.14 | 269.86 | 424.49 | 105.42 |
| Topocalma | 390.93 | 24.74 | 223.82 | 40.03 | 373.11 | 319.35 | 0.00 |
| ***B. linearis*** |  |  |  |  |  |  |  |
| Cavilolén | 134.35 | 253.39 | 44.54 | 307.11 | 115.93 | 585.87 | 267.34 |
| Pichidangui | 171.10 | 210.87 | 0.00 | 263.73 | 152.73 | 541.80 | 223.82 |
| Pichilemu | 430.94 | 56.47 | 263.73 | 0.00 | 413.12 | 280.51 | 40.03 |
| Quinicabén | 370.93 | 14.56 | 202.68 | 61.35 | 352.97 | 341.59 | 22.70 |
| Quintay | 287.97 | 92.35 | 118.77 | 145.14 | 269.86 | 424.49 | 105.42 |
| Topocalma | 390.93 | 24.74 | 223.82 | 40.03 | 373.11 | 319.35 | 0.00 |
| ***B. macraei*** |  |  |  |  |  |  |  |
| Huentelauquén | 111.70 | 269.54 | 59.41 | 321.47 | 93.32 | 597.75 | 281.45 |
| Navidad | 380.26 | 0.00 | 210.87 | 56.47 | 362.18 | 336.58 | 24.74 |
| Pichidangui | 171.10 | 210.87 | 0.00 | 263.73 | 152.73 | 541.80 | 223.82 |
| Pichilemu | 430.94 | 56.47 | 263.73 | 0.00 | 413.12 | 280.51 | 40.03 |
| Quintay | 287.97 | 92.35 | 118.77 | 145.14 | 269.86 | 424.49 | 105.42 |
| Topocalma | 390.93 | 24.74 | 223.82 | 40.03 | 373.11 | 319.35 | 0.00 |
| ***B. vernalis*** |  |  |  |  |  |  |  |
| Fray Jorge | 0.00 | 380.26 | 171.10 | 430.94 | 18.47 | 703.93 | 390.93 |
| Navidad | 380.26 | 0.00 | 210.87 | 56.47 | 362.18 | 336.58 | 24.74 |
| Pichidangui | 171.10 | 210.87 | 0.00 | 263.73 | 152.73 | 541.80 | 223.82 |
| Pichilemu | 430.94 | 56.47 | 263.73 | 0.00 | 413.12 | 280.51 | 40.03 |
| Talinay | 18.47 | 362.18 | 152.73 | 413.12 | 0.00 | 686.81 | 373.11 |
| Hualpén | 703.93 | 336.58 | 541.80 | 280.51 | 686.81 | 0.00 | 319.35 |
| Topocalma | 390.93 | 24.74 | 223.82 | 40.03 | 373.11 | 319.35 | 0.00 |

**Appendix S7 – D statistics and f4-ratio**

**Table S1:** D statistics and f4-ratio of different Baccharis species. Haplopappus was used as an outgroup. Taxa were divided into subgroups (North, South Quintay, Hualpén) according to their geography Population 1 = P1, Population 2 = P2, Population 3 = P3. D = Patterson's D.

| **P1** | **P2** | **P3** | **D** | **Z-score** | **p-value** | **f4-ratio** | **BBAA** | **ABBA** | **BABA** |
| --- | --- | --- | --- | --- | --- | --- | --- | --- | --- |
| B.int–North | B.ver–Hualpén | B.mac–South | 0.19 | 6.04 | 0.00 | 0.24 | 36.52 | 87.18 | 59.58 |
| B.mac–South | B.ver–Hualpén | B.lin–North | 0.17 | 4.03 | 0.00 | 0.06 | 137.10 | 40.74 | 28.65 |
| B.mac–South | B.ver–Hualpén | B.lin–Quintay | 0.17 | 3.77 | 0.00 | 0.06 | 136.63 | 39.95 | 28.62 |
| B.int–North | B.ver–Hualpén | B.mac–Quintay | 0.14 | 5.22 | 0.00 | 0.27 | 35.47 | 83.46 | 62.89 |
| B.mac–South | B.ver–Hualpén | B.lin–South | 0.11 | 3.13 | 0.00 | 0.04 | 136.23 | 39.04 | 31.18 |
| B.int–South | B.ver–Hualpén | B.mac–North | 0.11 | 3.53 | 0.00 | 0.16 | 39.61 | 80.27 | 64.54 |
| B.int–South | B.ver–Hualpén | B.mac–Quintay | 0.09 | 3.54 | 0.00 | 0.20 | 37.98 | 80.50 | 66.97 |
| B.int–North | B.ver–South | B.mac–South | 0.23 | 8.68 | 0.00 | 0.30 | 35.38 | 93.61 | 58.03 |
| B.int–North | B.ver–South | B.mac–Quintay | 0.16 | 6.71 | 0.00 | 0.33 | 35.80 | 87.48 | 62.72 |
| B.int–South | B.ver–South | B.mac–North | 0.15 | 5.64 | 0.00 | 0.22 | 39.44 | 84.60 | 62.34 |
| B.int–Quintay | B.ver–South | B.mac–South | 0.13 | 5.19 | 0.00 | 0.19 | 35.17 | 83.66 | 63.79 |
| B.mac–South | B.ver–South | B.lin–North | 0.12 | 4.27 | 0.00 | 0.04 | 146.72 | 37.23 | 29.08 |
| B.int–South | B.ver–South | B.mac–Quintay | 0.12 | 5.05 | 0.00 | 0.26 | 38.67 | 83.40 | 65.56 |
| B.int–Quintay | B.ver–South | B.mac–North | 0.11 | 4.14 | 0.00 | 0.17 | 37.49 | 79.87 | 64.36 |
| B.int–North | B.ver–South | B.mac–North | 0.10 | 3.77 | 0.00 | 0.17 | 32.62 | 84.73 | 69.05 |
| B.mac–South | B.ver–South | B.lin–Quintay | 0.09 | 3.24 | 0.00 | 0.03 | 146.75 | 36.54 | 30.24 |
| B.ver–Hualpén | B.ver–South | B.mac–South | 0.09 | 3.18 | 0.00 | 0.09 | 65.81 | 46.77 | 38.96 |
| B.int–South | B.ver–North | B.mac–North | 0.19 | 6.95 | 0.00 | 0.28 | 34.36 | 86.69 | 59.06 |
| B.int–North | B.ver–North | B.mac–Quintay | 0.18 | 7.13 | 0.00 | 0.34 | 36.06 | 85.02 | 59.05 |
| B.mac–South | B.ver–North | B.lin–North | 0.16 | 4.15 | 0.00 | 0.05 | 139.61 | 37.14 | 26.96 |
| B.int–Quintay | B.ver–North | B.mac–North | 0.15 | 5.57 | 0.00 | 0.22 | 33.96 | 80.82 | 59.94 |
| B.int–North | B.ver–North | B.mac–North | 0.14 | 5.18 | 0.00 | 0.22 | 31.07 | 84.61 | 63.56 |
| B.mac–South | B.ver–North | B.lin–Quintay | 0.14 | 3.53 | 0.00 | 0.05 | 139.93 | 36.37 | 27.62 |
| B.int–South | B.ver–North | B.mac–Quintay | 0.13 | 5.29 | 0.00 | 0.27 | 35.20 | 82.95 | 63.89 |
| B.mac–North | B.ver–North | B.lin–North | 0.12 | 3.20 | 0.00 | 0.04 | 140.40 | 34.42 | 27.08 |
| B.int–Quintay | B.ver–North | B.mac–South | 0.11 | 4.08 | 0.00 | 0.15 | 35.21 | 78.48 | 62.94 |
| B.mac–South | B.ver–North | B.lin–South | 0.10 | 3.02 | 0.00 | 0.04 | 138.77 | 35.95 | 29.50 |
| B.mac–North | B.mac–South | B.int–South | 0.21 | 8.00 | 0.00 | 1.16 | 107.46 | 47.30 | 30.79 |
| B.ver–North | B.mac–South | B.int–North | 0.20 | 6.86 | 0.00 | 0.44 | 87.25 | 56.00 | 37.29 |
| B.mac–Quintay | B.mac–South | B.int–South | 0.15 | 6.60 | 0.00 | 1.19 | 104.73 | 47.17 | 34.65 |
| B.lin–North | B.mac–South | B.int–South | 0.09 | 3.18 | 0.00 | 1.05 | 19.16 | 124.31 | 104.41 |
| B.mac–Quintay | B.mac–South | B.ver–South | 0.08 | 3.52 | 0.00 | 0.17 | 87.56 | 41.87 | 35.32 |
| B.mac–South | B.mac–Quintay | B.lin–North | 0.26 | 9.73 | 0.00 | 0.08 | 183.45 | 35.52 | 20.73 |
| B.mac–South | B.mac–Quintay | B.lin–Quintay | 0.25 | 9.22 | 0.00 | 0.07 | 183.37 | 35.32 | 21.23 |
| B.mac–South | B.mac–Quintay | B.lin–South | 0.22 | 8.36 | 0.00 | 0.07 | 181.46 | 34.71 | 22.18 |
| B.mac–North | B.mac–Quintay | B.lin–North | 0.21 | 7.57 | 0.00 | 0.06 | 179.04 | 34.64 | 22.70 |
| B.mac–North | B.mac–Quintay | B.lin–Quintay | 0.21 | 7.51 | 0.00 | 0.06 | 179.45 | 34.98 | 22.94 |
| B.mac–North | B.mac–Quintay | B.lin–South | 0.20 | 8.03 | 0.00 | 0.07 | 178.41 | 35.11 | 23.17 |

**Table S2:** D statistics and f4-ratio of different Baccharis species. Haplopappus was used as an outgroup. Taxa were divided into subgroups (North, South Quintay, Hualpén) according to their geography Population 1 = P1, Population 2 = P2, Population 3 = P3. D = Patterson's D (continued).

| **P1** | **P2** | **P3** | **D** | **Z-score** | **p-**  **value** | **f4-ratio** | **BBAA** | **ABBA** | **BABA** |
| --- | --- | --- | --- | --- | --- | --- | --- | --- | --- |
| B.ver–South | B.mac–Quintay | B.lin–South | 0.11 | 4.85 | 0.00 | 0.05 | 137.09 | 42.57 | 34.46 |
| B.ver–South | B.mac–Quintay | B.lin–Quintay | 0.10 | 4.20 | 0.00 | 0.04 | 137.68 | 41.88 | 34.08 |
| B.lin–North | B.mac–Quintay | B.int–Quintay | 0.09 | 3.35 | 0.00 | 1.04 | 26.58 | 113.90 | 95.22 |
| B.ver–South | B.mac–Quintay | B.lin–North | 0.09 | 3.67 | 0.00 | 0.04 | 137.30 | 40.99 | 34.35 |
| B.lin–South | B.mac–Quintay | B.int–Quintay | 0.09 | 3.30 | 0.00 | 1.05 | 27.31 | 113.47 | 95.40 |
| B.ver–North | B.mac–Quintay | B.lin–South | 0.08 | 3.36 | 0.00 | 0.03 | 134.58 | 38.85 | 32.77 |
| B.mac–North | B.mac–Quintay | B.int–Quintay | 0.08 | 3.73 | 0.00 | 1.63 | 100.39 | 43.25 | 36.53 |
| B.mac–South | B.mac–North | B.int–North | 0.19 | 6.92 | 0.00 | 0.59 | 113.22 | 43.13 | 29.34 |
| B.mac–Quintay | B.mac–North | B.int–North | 0.12 | 5.08 | 0.00 | 0.50 | 108.14 | 44.10 | 34.60 |
| B.ver–Hualpén | B.lin–South | B.int–North | 0.18 | 6.76 | 0.00 | 0.75 | 30.87 | 112.32 | 77.41 |
| B.ver–South | B.lin–South | B.int–North | 0.17 | 6.55 | 0.00 | 0.74 | 29.99 | 114.87 | 80.94 |
| B.ver–North | B.lin–South | B.int–North | 0.16 | 5.81 | 0.00 | 0.71 | 27.89 | 110.74 | 80.75 |
| B.ver–Hualpén | B.lin–South | B.int–South | 0.14 | 5.24 | 0.00 | 0.66 | 29.67 | 108.06 | 81.69 |
| B.ver–North | B.lin–South | B.int–South | 0.13 | 5.15 | 0.00 | 0.64 | 28.52 | 108.32 | 82.59 |
| B.ver–South | B.lin–South | B.int–South | 0.12 | 4.75 | 0.00 | 0.63 | 28.76 | 110.58 | 86.66 |
| B.ver–Hualpén | B.lin–South | B.int–Quintay | 0.09 | 3.22 | 0.00 | 0.51 | 29.94 | 102.70 | 86.29 |
| B.ver–South | B.lin–South | B.int–Quintay | 0.08 | 3.07 | 0.00 | 0.50 | 29.16 | 105.36 | 89.85 |
| B.lin–North | B.lin–South | B.int–South | 0.06 | 3.08 | 0.00 | 0.23 | 128.81 | 36.65 | 32.31 |
| B.ver–Hualpén | B.lin–Quintay | B.int–North | 0.19 | 6.63 | 0.00 | 0.81 | 30.69 | 114.09 | 77.01 |
| B.ver–South | B.lin–Quintay | B.int–North | 0.18 | 6.62 | 0.00 | 0.80 | 28.81 | 117.23 | 80.89 |
| B.ver–North | B.lin–Quintay | B.int–North | 0.17 | 5.91 | 0.00 | 0.78 | 26.98 | 112.94 | 80.68 |
| B.ver–Hualpén | B.lin–Quintay | B.int–South | 0.13 | 4.53 | 0.00 | 0.62 | 30.56 | 106.51 | 81.94 |
| B.ver–North | B.lin–Quintay | B.int–South | 0.12 | 4.50 | 0.00 | 0.60 | 28.72 | 107.23 | 83.47 |
| B.ver–South | B.lin–Quintay | B.int–South | 0.11 | 4.19 | 0.00 | 0.58 | 28.67 | 109.64 | 87.46 |
| B.ver–Hualpén | B.lin–Quintay | B.int–Quintay | 0.09 | 3.22 | 0.00 | 0.56 | 29.87 | 103.41 | 85.65 |
| B.ver–South | B.lin–Quintay | B.int–Quintay | 0.09 | 3.19 | 0.00 | 0.54 | 28.19 | 106.75 | 89.76 |
| B.ver–Hualpén | B.lin–North | B.int–North | 0.19 | 6.71 | 0.00 | 0.81 | 31.37 | 114.18 | 77.00 |
| B.ver–South | B.lin–North | B.int–North | 0.18 | 6.55 | 0.00 | 0.80 | 29.65 | 116.40 | 80.91 |
| B.ver–North | B.lin–North | B.int–North | 0.17 | 5.98 | 0.00 | 0.77 | 27.90 | 112.61 | 80.34 |
| B.ver–Hualpén | B.lin–North | B.int–South | 0.12 | 4.10 | 0.00 | 0.55 | 31.33 | 104.50 | 82.56 |
| B.ver–North | B.lin–North | B.int–South | 0.11 | 4.11 | 0.00 | 0.53 | 29.77 | 104.84 | 83.54 |
| B.ver–South | B.lin–North | B.int–South | 0.10 | 3.64 | 0.00 | 0.50 | 29.73 | 106.85 | 87.96 |
| B.mac–South | B.int–South | B.int–North | 0.08 | 3.35 | 0.00 | 0.44 | 84.08 | 71.06 | 60.37 |
| B.int–North | B.int–South | B.ver–South | 0.06 | 3.56 | 0.00 | 0.08 | 88.16 | 61.77 | 54.83 |
| B.int–Quintay | B.int–South | B.lin–South | 0.04 | 3.08 | 0.00 | 0.06 | 78.28 | 71.94 | 66.31 |
| B.int–North | B.int–Quintay | B.ver–Hualpén | 0.09 | 4.16 | 0.00 | 0.09 | 87.12 | 60.47 | 50.66 |
| B.int–North | B.int–Quintay | B.ver–South | 0.09 | 5.38 | 0.00 | 0.11 | 87.76 | 61.76 | 52.02 |
| B.int–North | B.int–Quintay | B.ver–North | 0.06 | 3.05 | 0.00 | 0.08 | 84.12 | 59.01 | 52.32 |

**Table S3:** D statistics and f4-ratio of different Baccharis populations in Chile. Haplopappus was used as an outgroup (excluding B. vernalis and B. × intermedia). Population 1 = P1, Population 2 = P2, Population 3 = P3. D = Patterson's D.

| **P1** | **P2** | **P3** | **D** | **Z-score** | **p-**  **value** | **f4-**  **ratio** | **BBAA** | **ABBA** | **BABA** |
| --- | --- | --- | --- | --- | --- | --- | --- | --- | --- |
| B.mac–Navidad | B.mac–Pichilemu | B.lin–Quintay | 0.12 | 3.07 | 0.00 | 0.03 | 212.37 | 22.54 | 17.64 |
| B.lin–Pichilemu | B.lin–Quintay | B.mac–Quintay | 0.08 | 3.13 | 0.00 | 0.02 | 197.12 | 22.99 | 19.63 |
| B.lin–Pichilemu | B.lin–Cavilolén | B.mac–Quintay | 0.08 | 3.15 | 0.00 | 0.02 | 195.14 | 23.81 | 20.24 |
| B.mac–Navidad | B.mac–Pichilemu | B.lin–Cavilolen | 0.13 | 3.23 | 0.00 | 0.03 | 212.03 | 22.41 | 17.24 |
| B.mac–Pichilemu | B.mac–Quintay | B.lin–Quinicabén | 0.17 | 5.90 | 0.00 | 0.05 | 181.77 | 32.82 | 23.48 |
| B.mac–Pichilemu | B.mac–Quintay | B.lin–Quintay | 0.18 | 6.38 | 0.00 | 0.05 | 180.77 | 33.61 | 23.43 |
| B.mac–Pichidangui | B.mac–Quintay | B.lin–Pichidangui | 0.18 | 6.40 | 0.00 | 0.06 | 179.93 | 33.62 | 23.22 |
| B.mac–Pichilemu | B.mac–Quintay | B.lin–Topocalma | 0.18 | 6.41 | 0.00 | 0.05 | 180.84 | 33.39 | 23.28 |
| B.mac–Pichilemu | B.mac–Quintay | B.lin–Pichilemu | 0.19 | 6.55 | 0.00 | 0.05 | 182.59 | 33.22 | 22.81 |
| B.mac–Pichidangui | B.mac–Quintay | B.lin–Pichilemu | 0.19 | 6.55 | 0.00 | 0.06 | 182.53 | 34.38 | 23.46 |
| B.mac–Pichilemu | B.mac–Quintay | B.lin–Cavilolén | 0.19 | 6.59 | 0.00 | 0.06 | 180.06 | 33.32 | 22.84 |
| B.mac–Pichidangui | B.mac–Quintay | B.lin–Quinicabén | 0.20 | 7.13 | 0.00 | 0.06 | 182.05 | 34.43 | 22.95 |
| B.mac–Pichidangui | B.mac–Quintay | B.lin–Cavilolén | 0.20 | 7.15 | 0.00 | 0.06 | 179.37 | 34.66 | 23.07 |
| B.mac–Pichidangui | B.mac–Quintay | B.lin–Topocalma | 0.20 | 7.17 | 0.00 | 0.06 | 180.70 | 34.61 | 23.05 |
| B.mac–Huentel  auquén | B.mac–Quintay | B.lin–Quinicabén | 0.21 | 7.20 | 0.00 | 0.06 | 181.22 | 33.79 | 22.11 |
| B.mac–Pichidangui | B.mac–Quintay | B.lin–Quintay | 0.20 | 7.24 | 0.00 | 0.06 | 180.29 | 35.23 | 23.29 |
| B.mac–Huentel  auquén | B.mac–Quintay | B.lin–Pichilemu | 0.21 | 7.26 | 0.00 | 0.06 | 181.93 | 34.14 | 22.07 |
| B.mac–Pichilemu | B.mac–Quintay | B.lin–Pichidangui | 0.21 | 7.38 | 0.00 | 0.06 | 181.03 | 33.35 | 21.99 |
| B.mac–Huentelauquén | B.mac–Quintay | B.lin–Pichidangui | 0.22 | 7.40 | 0.00 | 0.06 | 179.39 | 33.44 | 21.58 |
| B.mac–Huentelauquén | B.mac–Quintay | B.lin–Topocalma | 0.22 | 7.42 | 0.00 | 0.06 | 179.91 | 34.07 | 22.00 |
| B.mac–Huentelauquén | B.mac–Quintay | B.lin–Quintay | 0.23 | 7.89 | 0.00 | 0.07 | 179.73 | 34.86 | 21.87 |
| B.mac–Huentelauquén | B.mac–Quintay | B.lin–Cavilolén | 0.23 | 8.04 | 0.00 | 0.07 | 179.00 | 34.48 | 21.37 |
| B.mac–Topocalma | B.mac–Quintay | B.lin–Quinicabén | 0.22 | 8.21 | 0.00 | 0.07 | 184.48 | 34.92 | 22.11 |
| B.mac–Topocalma | B.mac–Quintay | B.lin–Topocalma | 0.24 | 8.72 | 0.00 | 0.07 | 183.48 | 35.50 | 21.90 |
| B.mac–Topocalma | B.mac–Quintay | B.lin–Quintay | 0.25 | 8.95 | 0.00 | 0.07 | 183.70 | 35.90 | 21.74 |
| B.mac–Navidad | B.mac–Quintay | B.lin–Quinicabén | 0.25 | 8.97 | 0.00 | 0.07 | 185.02 | 34.71 | 20.96 |
| B.mac–Topocalma | B.mac–Quintay | B.lin–Pichilemu | 0.25 | 8.98 | 0.00 | 0.07 | 185.35 | 35.47 | 21.23 |
| B.mac–Topocalma | B.mac–Quintay | B.lin–Cavilolén | 0.26 | 9.52 | 0.00 | 0.08 | 183.15 | 35.93 | 21.08 |
| B.mac–Navidad | B.mac–Quintay | B.lin–Quintay | 0.27 | 9.57 | 0.00 | 0.08 | 184.14 | 35.70 | 20.74 |
| B.mac–Navidad | B.mac–Quintay | B.lin–Pichilemu | 0.27 | 9.60 | 0.00 | 0.08 | 185.99 | 35.35 | 20.38 |
| B.mac–Navidad | B.mac–Quintay | B.lin–Topocalma | 0.26 | 9.62 | 0.00 | 0.08 | 184.19 | 35.46 | 20.73 |
| B.mac–Topocalma | B.mac–Quintay | B.lin–Pichidangui | 0.27 | 9.93 | 0.00 | 0.08 | 183.75 | 35.58 | 20.45 |
| B.mac–Navidad | B.mac–Quintay | B.lin–Cavilolén | 0.28 | 10.04 | 0.00 | 0.08 | 183.56 | 35.69 | 20.13 |
| B.mac–Navidad | B.mac–Quintay | B.lin–Pichidangui | 0.28 | 10.35 | 0.00 | 0.08 | 184.16 | 35.34 | 19.69 |

**Table S4:** D statistics and f4-ratio of different Baccharis populations in Chile. Haplopappus was used as an outgroup (excluding B. × intermedia). Population 1 = P1, Population 2 = P2, Population 3 = P3. D = Patterson's D.

| **P1** | **P2** | **P3** | **D** | **Z-score** | **p-**  **value** | **f4-ratio** | **BBAA** | **ABBA** | **BABA** |
| --- | --- | --- | --- | --- | --- | --- | --- | --- | --- |
| B.lin–Pichilemu | B.lin–Cavilolén | B.mac–Quintay | 0.09 | 3.28 | 0.00 | 0.03 | 196.56 | 24.23 | 20.42 |
| B.lin–Pichilemu | B.lin–Topocalma | B.ver–Navidad | 0.10 | 3.13 | 0.00 | 0.02 | 209.38 | 21.94 | 18.00 |
| B.mac–Navidad | B.mac–Quintay | B.lin–Pichidangui | 0.28 | 10.10 | 0.00 | 0.08 | 185.64 | 36.58 | 20.63 |
| B.mac–Navidad | B.mac–Quintay | B.lin–Cavilolén | 0.27 | 9.86 | 0.00 | 0.08 | 183.98 | 36.60 | 20.87 |
| B.mac–Topocalma | B.mac–Quintay | B.lin–Pichidangui | 0.26 | 9.63 | 0.00 | 0.08 | 185.16 | 36.58 | 21.43 |
| B.mac–Navidad | B.mac–Quintay | B.lin–Topocalma | 0.26 | 9.59 | 0.00 | 0.08 | 185.61 | 36.04 | 21.05 |
| B.mac–Navidad | B.mac–Quintay | B.lin–Quintay | 0.26 | 9.24 | 0.00 | 0.08 | 185.21 | 35.92 | 21.23 |
| B.mac–Topocalma | B.mac–Quintay | B.lin–Cavilolén | 0.25 | 9.18 | 0.00 | 0.07 | 183.37 | 36.52 | 21.93 |
| B.mac–Navidad | B.mac–Quintay | B.lin–Pichilemu | 0.25 | 9.05 | 0.00 | 0.07 | 187.37 | 35.66 | 21.19 |
| B.mac–Navidad | B.mac–Quintay | B.lin–Quinicabén | 0.25 | 9.00 | 0.00 | 0.07 | 185.96 | 35.56 | 21.43 |
| B.mac–Topocalma | B.mac–Quintay | B.lin–Topocalma | 0.23 | 8.43 | 0.00 | 0.07 | 184.73 | 35.78 | 22.43 |
| B.mac–Topocalma | B.mac–Quintay | B.lin–Quintay | 0.23 | 8.34 | 0.00 | 0.07 | 184.53 | 35.79 | 22.46 |
| B.mac–Topocalma | B.mac–Quintay | B.lin–Pichilemu | 0.23 | 8.22 | 0.00 | 0.07 | 186.49 | 35.38 | 22.14 |
| B.mac–Huentelauquén | B.mac–Quintay | B.lin–Cavilolén | 0.23 | 8.11 | 0.00 | 0.07 | 179.52 | 35.63 | 22.09 |
| B.mac–Topocalma | B.mac–Quintay | B.lin–Quinicabén | 0.22 | 7.91 | 0.00 | 0.06 | 185.15 | 35.38 | 22.80 |
| B.mac–Huentelauquén | B.mac–Quintay | B.lin–Quintay | 0.23 | 7.80 | 0.00 | 0.07 | 180.76 | 35.21 | 22.28 |
| B.mac–Huentelauquén | B.mac–Quintay | B.lin–Pichilemu | 0.22 | 7.67 | 0.00 | 0.07 | 183.39 | 35.36 | 22.37 |
| B.mac–Huentelauquén | B.mac–Quintay | B.lin–Pichidangui | 0.22 | 7.57 | 0.00 | 0.06 | 180.37 | 34.82 | 22.37 |
| B.mac–Huentelauquén | B.mac–Quintay | B.lin–Quinicabén | 0.21 | 7.43 | 0.00 | 0.06 | 181.63 | 34.95 | 22.66 |
| B.mac–Huentelauquén | B.mac–Quintay | B.lin–Topocalma | 0.21 | 7.43 | 0.00 | 0.06 | 180.53 | 34.63 | 22.42 |
| B.mac–Pichilemu | B.mac–Quintay | B.lin–Pichidangui | 0.20 | 7.07 | 0.00 | 0.06 | 182.27 | 34.66 | 23.25 |
| B.mac–Pichidangui | B.mac–Quintay | B.lin–Cavilolén | 0.20 | 7.03 | 0.00 | 0.06 | 179.62 | 35.65 | 24.00 |
| B.mac–Pichidangui | B.mac–Quintay | B.lin–Quinicabén | 0.19 | 7.00 | 0.00 | 0.06 | 182.04 | 35.33 | 23.84 |
| B.mac–Pichilemu | B.mac–Quintay | B.lin–Cavilolén | 0.19 | 6.69 | 0.00 | 0.06 | 180.41 | 34.63 | 23.64 |
| B.mac–Pichidangui | B.mac–Quintay | B.lin–Pichilemu | 0.19 | 6.58 | 0.00 | 0.06 | 183.44 | 35.36 | 24.14 |
| B.mac–Pichidangui | B.mac–Quintay | B.lin–Quintay | 0.18 | 6.54 | 0.00 | 0.06 | 180.77 | 35.19 | 24.27 |
| B.mac–Pichidangui | B.mac–Quintay | B.lin–Topocalma | 0.18 | 6.52 | 0.00 | 0.06 | 180.67 | 34.71 | 24.04 |
| B.mac–Pichilemu | B.mac–Quintay | B.lin–Topocalma | 0.18 | 6.51 | 0.00 | 0.06 | 182.03 | 34.31 | 23.79 |
| B.mac–Pichidangui | B.mac–Quintay | B.lin–Pichidangui | 0.18 | 6.41 | 0.00 | 0.05 | 180.38 | 34.83 | 24.12 |
| B.mac–Pichilemu | B.mac–Quintay | B.lin–Quintay | 0.17 | 6.24 | 0.00 | 0.05 | 181.55 | 34.19 | 24.02 |
| B.mac–Pichilemu | B.mac–Quintay | B.lin–Quinicabén | 0.17 | 6.03 | 0.00 | 0.05 | 182.53 | 33.94 | 24.09 |
| B.mac–Pichilemu | B.mac–Quintay | B.lin–Pichilemu | 0.17 | 6.01 | 0.00 | 0.05 | 183.59 | 33.64 | 23.82 |
| B.ver–Topocalma | B.mac–Quintay | B.lin–Quintay | 0.17 | 5.98 | 0.00 | 0.07 | 139.96 | 43.41 | 30.94 |
| B.ver–Navidad | B.mac–Quintay | B.lin–Quintay | 0.17 | 5.97 | 0.00 | 0.07 | 138.42 | 43.64 | 31.03 |
| B.ver–Navidad | B.mac–Quintay | B.lin–Pichilemu | 0.16 | 5.68 | 0.00 | 0.06 | 141.14 | 43.33 | 31.06 |
| B.ver–Topocalma | B.mac–Quintay | B.lin–Cavilolén | 0.16 | 5.58 | 0.00 | 0.06 | 138.48 | 42.98 | 31.35 |
| B.ver–Navidad | B.mac–Quintay | B.lin–Cavilolén | 0.16 | 5.58 | 0.00 | 0.06 | 136.85 | 43.22 | 31.48 |
| B.ver–Topocalma | B.mac–Quintay | B.lin–Pichilemu | 0.16 | 5.55 | 0.00 | 0.06 | 142.48 | 42.94 | 31.11 |
| B.ver–Pichilemu | B.mac–Quintay | B.lin–Quintay | 0.16 | 5.48 | 0.00 | 0.06 | 139.30 | 43.22 | 31.56 |
| B.ver–Pichilemu | B.mac–Quintay | B.lin–Cavilolén | 0.15 | 5.28 | 0.00 | 0.06 | 137.73 | 42.87 | 31.70 |

**Table S5:** D statistics and f4-ratio of different Baccharis populations in Chile. Haplopappus was used as an outgroup (excluding B. × intermedia). Population 1 = P1, Population 2 = P2, Population 3 = P3. D = Patterson's D (continued).

| **P1** | **P2** | **P3** | **D** | **Z-score** | **p-**  **value** | **f4-ratio** | **BBAA** | **ABBA** | **BABA** |
| --- | --- | --- | --- | --- | --- | --- | --- | --- | --- |
| B.ver–Topocalma | B.mac–Quintay | B.lin–Topocalma | 0.15 | 5.24 | 0.00 | 0.06 | 139.56 | 42.81 | 31.82 |
| B.ver–Navidad | B.mac–Quintay | B.lin–Topocalma | 0.15 | 5.23 | 0.00 | 0.06 | 138.14 | 43.21 | 32.04 |
| B.ver–Pichilemu | B.mac–Quintay | B.lin–Pichilemu | 0.15 | 5.12 | 0.00 | 0.06 | 141.64 | 42.71 | 31.70 |
| B.ver–Topocalma | B.mac–Quintay | B.lin–Quinicabén | 0.13 | 4.67 | 0.00 | 0.05 | 140.50 | 42.28 | 32.33 |
| B.ver–Navidad | B.mac–Quintay | B.lin–Pichidangui | 0.13 | 4.65 | 0.00 | 0.05 | 137.74 | 42.42 | 32.42 |
| B.ver–Pichilemu | B.mac–Quintay | B.lin–Topocalma | 0.13 | 4.63 | 0.00 | 0.05 | 138.74 | 42.57 | 32.70 |
| B.ver–Navidad | B.mac–Quintay | B.lin–Quinicabén | 0.13 | 4.63 | 0.00 | 0.05 | 139.01 | 42.49 | 32.48 |
| B.ver–Topocalma | B.mac–Quintay | B.lin–Pichidangui | 0.13 | 4.59 | 0.00 | 0.05 | 139.38 | 42.18 | 32.44 |
| B.ver–Pichilemu | B.mac–Quintay | B.lin–Pichidangui | 0.12 | 4.14 | 0.00 | 0.05 | 138.54 | 42.06 | 33.12 |
| B.ver–Pichilemu | B.mac–Quintay | B.lin–Quinicabén | 0.12 | 4.09 | 0.00 | 0.05 | 139.63 | 41.96 | 33.12 |
| B.ver–Fray Jorge | B.mac–Quintay | B.lin–Quintay | 0.09 | 3.13 | 0.00 | 0.04 | 136.56 | 39.31 | 32.79 |
| B.ver–Fray Jorge | B.mac–Quintay | B.lin–Pichilemu | 0.09 | 3.05 | 0.00 | 0.03 | 138.88 | 39.56 | 33.01 |
| B.mac–Quintay | B.mac–Topocalma | B.ver–Pichilemu | 0.09 | 3.33 | 0.00 | 0.13 | 88.46 | 42.77 | 35.52 |
| B.mac–Huentelauquén | B.mac–Topocalma | B.ver–Pichilemu | 0.11 | 3.27 | 0.00 | 0.15 | 90.22 | 41.98 | 33.40 |
| B.mac–Quintay | B.mac–Topocalma | B.ver–Navidad | 0.09 | 3.08 | 0.00 | 0.12 | 89.35 | 41.68 | 35.11 |
| B.mac–Navidad | B.ver–Fray Jorge | B.lin–Pichidangui | 0.17 | 3.72 | 0.00 | 0.05 | 142.50 | 38.15 | 27.32 |
| B.mac–Navidad | B.ver–Fray Jorge | B.lin–Topocalma | 0.15 | 3.48 | 0.00 | 0.05 | 141.87 | 37.04 | 27.51 |
| B.mac–Topocalma | B.ver–Fray Jorge | B.lin–Pichidangui | 0.15 | 3.44 | 0.00 | 0.05 | 142.70 | 38.09 | 28.06 |
| B.mac–Navidad | B.ver–Fray Jorge | B.lin–Cavilolén | 0.14 | 3.32 | 0.00 | 0.05 | 141.19 | 37.28 | 27.86 |
| B.mac–Navidad | B.ver–Fray Jorge | B.lin–Quinicabén | 0.13 | 3.04 | 0.00 | 0.04 | 142.63 | 36.85 | 28.40 |
| B.mac–Navidad | B.ver–Pichidangui | B.lin–Pichidangui | 0.21 | 4.72 | 0.00 | 0.07 | 140.34 | 40.31 | 26.28 |
| B.mac–Navidad | B.ver–Pichidangui | B.lin–Topocalma | 0.20 | 4.63 | 0.00 | 0.07 | 139.38 | 39.24 | 26.27 |
| B.mac–Topocalma | B.ver–Pichidangui | B.lin–Pichidangui | 0.20 | 4.51 | 0.00 | 0.07 | 141.88 | 39.97 | 26.73 |
| B.ver–Navidad | B.ver–Pichidangui | B.lin–Topocalma | 0.17 | 4.33 | 0.00 | 0.05 | 149.64 | 32.26 | 23.10 |
| B.ver–Topocalma | B.ver–Pichidangui | B.lin–Topocalma | 0.16 | 4.27 | 0.00 | 0.05 | 151.24 | 32.10 | 23.13 |
| B.mac–Navidad | B.ver–Pichidangui | B.lin–Quinicabén | 0.18 | 4.20 | 0.00 | 0.06 | 139.90 | 38.61 | 26.80 |
| B.ver–Navidad | B.ver–Pichidangui | B.lin–Pichilemu | 0.17 | 4.19 | 0.00 | 0.05 | 153.35 | 31.95 | 22.87 |
| B.mac–Navidad | B.ver–Pichidangui | B.lin–Cavilolén | 0.18 | 4.13 | 0.00 | 0.06 | 138.91 | 38.48 | 26.73 |
| B.mac–Topocalma | B.ver–Pichidangui | B.lin–Topocalma | 0.17 | 4.07 | 0.00 | 0.06 | 140.69 | 38.71 | 27.38 |
| B.ver–Topocalma | B.ver–Pichidangui | B.lin–Pichilemu | 0.16 | 4.01 | 0.00 | 0.04 | 154.76 | 31.67 | 23.04 |
| B.ver–Navidad | B.ver–Pichidangui | B.lin–Quintay | 0.16 | 3.98 | 0.00 | 0.04 | 151.09 | 31.74 | 23.17 |
| B.ver–Topocalma | B.ver–Pichidangui | B.lin–Quintay | 0.15 | 3.94 | 0.00 | 0.04 | 152.77 | 31.69 | 23.26 |
| B.mac–Navidad | B.ver–Pichidangui | B.lin–Pichilemu | 0.17 | 3.92 | 0.00 | 0.06 | 141.58 | 38.12 | 26.86 |
| B.mac–Navidad | B.ver–Pichidangui | B.lin–Quintay | 0.17 | 3.78 | 0.00 | 0.06 | 139.58 | 37.54 | 26.88 |
| B.mac–Topocalma | B.ver–Pichidangui | B.lin–Cavilolén | 0.16 | 3.77 | 0.00 | 0.05 | 140.42 | 38.16 | 27.53 |
| B.ver–Navidad | B.ver–Pichidangui | B.lin–Pichidangui | 0.15 | 3.69 | 0.00 | 0.04 | 149.03 | 31.24 | 23.16 |
| B.mac–Topocalma | B.ver–Pichidangui | B.lin–Quinicabén | 0.16 | 3.68 | 0.00 | 0.05 | 141.22 | 38.09 | 27.85 |
| B.mac–Huentelauquén | B.ver–Pichidangui | B.lin–Topocalma | 0.16 | 3.66 | 0.00 | 0.05 | 141.94 | 36.58 | 26.39 |
| B.ver–Pichilemu | B.ver–Pichidangui | B.lin–Topocalma | 0.14 | 3.64 | 0.00 | 0.04 | 150.23 | 32.32 | 24.43 |

**Table S6:** D statistics and f4-ratio of different Baccharis populations in Chile. Haplopappus was used as an outgroup (excluding B. × intermedia). Population 1 = P1, Population 2 = P2, Population 3 = P3. D = Patterson's D (continued).

| **P1** | **P2** | **P3** | **D** | **Z-**  **score** | **p-**  **value** | **f4-**  **ratio** | **BBAA** | **ABBA** | **BABA** |
| --- | --- | --- | --- | --- | --- | --- | --- | --- | --- |
| B.ver–Navidad | B.ver–Pichidangui | B.lin–Cavilolén | 0.14 | 3.64 | 0.00 | 0.04 | 149.83 | 31.25 | 23.48 |
| B.ver–Navidad | B.ver–Pichidangui | B.lin–Quinicabén | 0.14 | 3.64 | 0.00 | 0.04 | 150.46 | 31.19 | 23.51 |
| B.ver–Topocalma | B.ver–Pichidangui | B.lin–Quinicabén | 0.14 | 3.64 | 0.00 | 0.04 | 152.11 | 31.19 | 23.56 |
| B.mac–Huentelauquén | B.ver–Pichidangui | B.lin–Quinicabén | 0.16 | 3.61 | 0.00 | 0.05 | 142.99 | 36.54 | 26.57 |
| B.mac–Huentelauquén | B.ver–Pichidangui | B.lin–Pichidangui | 0.16 | 3.59 | 0.00 | 0.05 | 142.53 | 37.18 | 26.65 |
| B.ver–Topocalma | B.ver–Pichidangui | B.lin–Cavilolén | 0.14 | 3.59 | 0.00 | 0.04 | 151.38 | 31.20 | 23.55 |
| B.ver–Topocalma | B.ver–Pichidangui | B.lin–Pichidangui | 0.14 | 3.57 | 0.00 | 0.04 | 150.60 | 31.14 | 23.32 |
| B.ver–Pichilemu | B.ver–Pichidangui | B.lin–Pichilemu | 0.14 | 3.55 | 0.00 | 0.04 | 153.80 | 32.02 | 24.16 |
| B.mac–Topocalma | B.ver–Pichidangui | B.lin–Pichilemu | 0.15 | 3.53 | 0.00 | 0.05 | 142.92 | 37.59 | 27.55 |
| B.mac–Pichidangui | B.ver–Pichidangui | B.lin–Quinicabén | 0.15 | 3.48 | 0.00 | 0.05 | 142.37 | 36.11 | 26.95 |
| B.mac–Huentelauquén | B.ver–Pichidangui | B.lin–Cavilolén | 0.15 | 3.46 | 0.00 | 0.05 | 141.86 | 36.20 | 26.64 |
| B.mac–Huentelauquén | B.ver–Pichidangui | B.lin–Pichilemu | 0.16 | 3.44 | 0.00 | 0.05 | 145.14 | 36.46 | 26.68 |
| B.ver–Pichilemu | B.ver–Pichidangui | B.lin–Quintay | 0.14 | 3.42 | 0.00 | 0.04 | 152.02 | 32.09 | 24.43 |
| B.mac–Topocalma | B.ver–Pichidangui | B.lin–Quintay | 0.14 | 3.31 | 0.00 | 0.05 | 141.10 | 37.22 | 27.92 |
| B.ver–Pichilemu | B.ver–Pichidangui | B.lin–Cavilolén | 0.13 | 3.30 | 0.00 | 0.04 | 150.70 | 31.66 | 24.44 |
| B.mac–Pichidangui | B.ver–Pichidangui | B.lin–Topocalma | 0.14 | 3.26 | 0.00 | 0.05 | 141.08 | 35.92 | 27.27 |
| B.ver–Fray Jorge | B.ver–Pichidangui | B.ver–Pichilemu | 0.09 | 3.24 | 0.00 | 0.17 | 55.00 | 45.64 | 38.34 |
| B.mac–Huentelauquén | B.ver–Pichidangui | B.lin–Quintay | 0.14 | 3.18 | 0.00 | 0.05 | 143.00 | 35.72 | 26.83 |
| B.mac–Pichidangui | B.ver–Pichidangui | B.lin–Pichidangui | 0.14 | 3.18 | 0.00 | 0.04 | 141.41 | 36.30 | 27.51 |
| B.ver–Pichilemu | B.ver–Pichidangui | B.lin–Pichidangui | 0.13 | 3.11 | 0.00 | 0.04 | 149.42 | 31.49 | 24.45 |
| B.mac–Pichilemu | B.ver–Pichidangui | B.lin–Pichidangui | 0.14 | 3.08 | 0.00 | 0.05 | 140.35 | 38.64 | 29.07 |
| B.ver–Fray Jorge | B.ver–Pichidangui | B.ver–Topocalma | 0.08 | 3.05 | 0.00 | 0.17 | 53.33 | 45.21 | 38.49 |
| B.ver–Pichilemu | B.ver–Pichidangui | B.mac–Huentelauquén | 0.10 | 3.04 | 0.00 | 0.09 | 60.80 | 48.08 | 39.53 |
| B.ver–Pichilemu | B.ver–Pichidangui | B.lin–Quinicabén | 0.12 | 3.01 | 0.00 | 0.03 | 151.07 | 31.34 | 24.79 |
| B.mac–Navidad | B.ver–Talinay | B.lin–Pichidangui | 0.19 | 4.34 | 0.00 | 0.06 | 141.63 | 38.30 | 26.14 |
| B.mac–Navidad | B.ver–Talinay | B.lin–Topocalma | 0.18 | 4.27 | 0.00 | 0.06 | 140.87 | 37.45 | 26.16 |
| B.mac–Topocalma | B.ver–Talinay | B.lin–Pichidangui | 0.17 | 4.11 | 0.00 | 0.06 | 141.47 | 38.17 | 26.80 |
| B.mac–Navidad | B.ver–Talinay | B.lin–Cavilolén | 0.17 | 3.97 | 0.00 | 0.05 | 140.14 | 37.21 | 26.47 |
| B.mac–Navidad | B.ver–Talinay | B.lin–Quinicabén | 0.16 | 3.95 | 0.00 | 0.05 | 141.44 | 37.40 | 26.81 |
| B.mac–Topocalma | B.ver–Talinay | B.lin–Topocalma | 0.15 | 3.67 | 0.00 | 0.05 | 140.49 | 37.15 | 27.50 |
| B.mac–Navidad | B.ver–Talinay | B.lin–Quintay | 0.15 | 3.60 | 0.00 | 0.05 | 140.90 | 36.27 | 26.64 |
| B.mac–Topocalma | B.ver–Talinay | B.lin–Cavilolén | 0.15 | 3.59 | 0.00 | 0.05 | 139.96 | 37.10 | 27.50 |
| B.mac–Navidad | B.ver–Talinay | B.lin–Pichilemu | 0.15 | 3.48 | 0.00 | 0.05 | 143.00 | 36.10 | 26.75 |
| B.ver–Topocalma | B.ver–Talinay | B.lin–Topocalma | 0.13 | 3.45 | 0.00 | 0.04 | 147.64 | 32.01 | 24.72 |
| B.ver–Topocalma | B.ver–Talinay | B.lin–Quintay | 0.13 | 3.45 | 0.00 | 0.04 | 148.97 | 31.98 | 24.59 |
| B.ver–Navidad | B.ver–Talinay | B.lin–Topocalma | 0.13 | 3.44 | 0.00 | 0.04 | 145.39 | 32.21 | 24.73 |
| B.ver–Navidad | B.ver–Talinay | B.lin–Quintay | 0.13 | 3.42 | 0.00 | 0.04 | 146.64 | 32.09 | 24.56 |

**Table S7:** D statistics and f4-ratio of different Baccharis populations in Chile. Haplopappus was used as an outgroup (excluding B. × intermedia). Population 1 = P1, Population 2 = P2, Population 3 = P3. D = Patterson's D (continued).

| **P1** | **P2** | **P3** | **D** | **Z-score** | **p-**  **value** | **f4-**  **ratio** | **BBAA** | **ABBA** | **BABA** |
| --- | --- | --- | --- | --- | --- | --- | --- | --- | --- |
| B.mac–Topocalma | B.ver–Talinay | B.lin–Quinicabén | 0.14 | 3.40 | 0.00 | 0.05 | 141.09 | 37.13 | 28.10 |
| B.ver–Navidad | B.ver–Talinay | B.lin–Pichilemu | 0.13 | 3.25 | 0.00 | 0.04 | 149.15 | 31.72 | 24.57 |
| B.mac–Huentelauquén | B.ver–Talinay | B.lin–Quinicabén | 0.14 | 3.22 | 0.00 | 0.05 | 142.62 | 36.04 | 27.30 |
| B.mac–Huentelauquén | B.ver–Talinay | B.lin–Topocalma | 0.14 | 3.18 | 0.00 | 0.04 | 141.44 | 35.44 | 26.93 |
| B.mac–Pichidangui | B.ver–Talinay | B.lin-Quinicabén | 0.13 | 3.16 | 0.00 | 0.04 | 142.15 | 35.15 | 27.21 |
| B.mac–Huentelauquén | B.ver–Talinay | B.lin–Cavilolén | 0.14 | 3.15 | 0.00 | 0.04 | 141.24 | 35.70 | 27.16 |
| B.ver–Topocalma | B.ver–Talinay | B.lin–Pichilemu | 0.12 | 3.14 | 0.00 | 0.03 | 151.25 | 31.45 | 24.74 |
| B.ver–Topocalma | B.ver–Talinay | B.lin–Cavilolén | 0.12 | 3.11 | 0.00 | 0.03 | 147.47 | 31.49 | 24.86 |
| B.mac–Topocalma | B.ver–Talinay | B.lin–Quintay | 0.13 | 3.11 | 0.00 | 0.04 | 140.71 | 36.14 | 27.88 |
| B.ver–Navidad | B.ver–Talinay | B.lin–Cavilolén | 0.12 | 3.10 | 0.00 | 0.04 | 145.26 | 31.56 | 24.82 |
| B.ver–Topocalma | B.ver–Talinay | B.lin–Quinicabén | 0.11 | 3.06 | 0.00 | 0.03 | 148.44 | 31.49 | 25.09 |
| B.mac–Topocalma | B.ver–Talinay | B.lin–Pichilemu | 0.13 | 3.06 | 0.00 | 0.04 | 142.64 | 35.78 | 27.65 |
| B.mac–Huentelauquén | B.ver–Talinay | B.lin–Pichidangui | 0.14 | 3.05 | 0.00 | 0.04 | 142.03 | 35.89 | 27.24 |
| B.mac–Navidad | B.ver–Hualpén | B.lin–Pichidangui | 0.21 | 4.51 | 0.00 | 0.07 | 140.31 | 42.48 | 28.01 |
| B.mac–Topocalma | B.ver–Hualpén | B.lin–Pichidangui | 0.19 | 4.35 | 0.00 | 0.07 | 139.43 | 42.03 | 28.35 |
| B.ver–Navidad | B.ver–Hualpén | B.lin–Quintay | 0.17 | 4.17 | 0.00 | 0.05 | 159.86 | 32.49 | 22.87 |
| B.ver–Topocalma | B.ver–Hualpén | B.lin–Quintay | 0.17 | 4.15 | 0.00 | 0.05 | 162.39 | 32.55 | 23.07 |
| B.mac–Navidad | B.ver–Hualpén | B.lin–Topocalma | 0.18 | 4.05 | 0.00 | 0.06 | 140.12 | 41.14 | 28.59 |
| B.mac–Navidad | B.ver–Hualpén | B.lin–Cavilolén | 0.18 | 3.93 | 0.00 | 0.06 | 139.26 | 41.24 | 28.78 |
| B.ver–Navidad | B.ver–Hualpén | B.lin–Pichilemu | 0.16 | 3.80 | 0.00 | 0.04 | 162.86 | 31.74 | 23.03 |
| B.ver–Navidad | B.ver–Hualpén | B.lin–Topocalma | 0.16 | 3.79 | 0.00 | 0.05 | 159.53 | 32.47 | 23.72 |
| B.mac–Navidad | B.ver–Hualpén | B.lin–Quintay | 0.17 | 3.77 | 0.00 | 0.06 | 139.71 | 40.07 | 28.35 |
| B.ver–Topocalma | B.ver–Hualpén | B.lin–Topocalma | 0.15 | 3.74 | 0.00 | 0.05 | 161.87 | 32.52 | 23.96 |
| B.ver–Pichilemu | B.ver–Hualpén | B.lin–Quintay | 0.15 | 3.72 | 0.00 | 0.05 | 162.33 | 32.52 | 23.80 |
| B.ver–Navidad | B.ver–Hualpén | B.lin–Pichidangui | 0.16 | 3.66 | 0.00 | 0.04 | 158.00 | 31.67 | 23.14 |
| B.ver–Navidad | B.ver–Hualpén | B.lin–Cavilolén | 0.15 | 3.65 | 0.00 | 0.04 | 158.82 | 32.10 | 23.63 |
| B.mac–Topocalma | B.ver–Hualpén | B.lin–Cavilolén | 0.16 | 3.64 | 0.00 | 0.06 | 138.42 | 40.85 | 29.52 |
| B.mac–Navidad | B.ver–Hualpén | B.lin–Quinicabén | 0.16 | 3.63 | 0.00 | 0.06 | 140.73 | 40.60 | 29.28 |
| B.ver–Topocalma | B.ver–Hualpén | B.lin–Cavilolén | 0.15 | 3.63 | 0.00 | 0.04 | 161.35 | 32.24 | 23.88 |
| B.ver–Topocalma | B.ver–Hualpén | B.lin–Pichilemu | 0.15 | 3.62 | 0.00 | 0.04 | 165.08 | 31.79 | 23.52 |
| B.ver–Topocalma | B.ver–Hualpén | B.lin–Pichidangui | 0.15 | 3.59 | 0.00 | 0.04 | 160.52 | 31.68 | 23.41 |
| B.mac–Topocalma | B.ver–Hualpén | B.lin–Topocalma | 0.16 | 3.58 | 0.00 | 0.06 | 139.00 | 40.53 | 29.62 |
| B.mac–Navidad | B.ver–Hualpén | B.lin–Pichilemu | 0.16 | 3.46 | 0.00 | 0.05 | 142.24 | 40.02 | 29.12 |
| B.mac–Topocalma | B.ver–Hualpén | B.lin–Quintay | 0.15 | 3.39 | 0.00 | 0.05 | 138.79 | 39.63 | 29.28 |
| B.ver–Pichilemu | B.ver–Hualpén | B.lin–Cavilolén | 0.14 | 3.38 | 0.00 | 0.04 | 161.31 | 32.21 | 24.28 |
| B.ver–Pichidangui | B.ver–Hualpén | B.ver–Pichilemu | 0.10 | 3.24 | 0.00 | 0.31 | 39.29 | 59.76 | 48.58 |
| B.ver–Pichilemu | B.ver–Hualpén | B.lin–Pichilemu | 0.13 | 3.23 | 0.00 | 0.04 | 164.93 | 31.52 | 24.02 |
| B.ver–Pichilemu | B.ver–Hualpén | B.lin–Topocalma | 0.13 | 3.19 | 0.00 | 0.04 | 161.77 | 32.33 | 24.84 |
| B.mac–Topocalma | B.ver–Hualpén | B.lin–Quinicabén | 0.14 | 3.19 | 0.00 | 0.05 | 139.66 | 40.04 | 30.29 |
| B.ver–Navidad | B.ver–Hualpén | B.lin–Quinicabén | 0.13 | 3.17 | 0.00 | 0.04 | 160.17 | 31.14 | 23.95 |

**Table S8:** D statistics and f4-ratio of different Baccharis populations in Chile. Haplopappus was used as an outgroup (excluding B. × intermedia). Population 1 = P1, Population 2 = P2, Population 3 = P3. D = Patterson's D (continued).

| **P1** | **P2** | **P3** | **D** | **Z-score** | **p-**  **value** | **f4-**  **ratio** | **BBAA** | **ABBA** | **BABA** |
| --- | --- | --- | --- | --- | --- | --- | --- | --- | --- |
| B.ver–Pichilemu | B.ver–Hualpén | B.lin–Pichidangui | 0.13 | 3.17 | 0.00 | 0.04 | 160.24 | 31.60 | 24.11 |
| B.mac–Pichilemu | B.ver–Hualpén | B.lin–Pichidangui | 0.14 | 3.15 | 0.00 | 0.05 | 137.33 | 39.57 | 29.56 |
| B.mac–Topocalma | B.ver–Hualpén | B.lin–Pichilemu | 0.14 | 3.14 | 0.00 | 0.05 | 141.16 | 39.45 | 29.77 |
| B.ver–Topocalma | B.ver–Hualpén | B.lin–Quinicabén | 0.13 | 3.14 | 0.00 | 0.04 | 162.67 | 31.47 | 24.34 |
| B.mac–Huentelauquén | B.ver–Hualpén | B.lin–Pichidangui | 0.15 | 3.06 | 0.00 | 0.06 | 135.10 | 42.40 | 31.43 |
| B.ver–Hualpén | B.ver–Topocalma | B.ver–Talinay | 0.10 | 3.47 | 0.00 | 0.30 | 61.44 | 46.52 | 37.91 |
| B.ver–Hualpén | B.ver–Topocalma | B.ver–Fray Jorge | 0.10 | 3.35 | 0.00 | 0.25 | 62.65 | 46.08 | 37.46 |

**Table S9:** Estimation of f4-ratios for all possible topologies of four Baccharis species in Chile, dividing the data set into 7 blocks of 500 bp each. Taxa were divided into subgroups (North, South Quintay, Hualpén) according to their geography.

| **Population 1** | **Population 2** | **Population 3** | **Population 4** | **f4 value** | **standard error** | **Z-score** |
| --- | --- | --- | --- | --- | --- | --- |
| *B. linearis*  North | *B. macraei*  North | *B. linearis*  South | *B. macraei* Quintay | 0.032323 | 0.001683 | 19.2065 |
| *B. linearis*  South | *B. macraei* Quintay | *B. linearis*  Quintay | *B. macraei*  North | 0.032382 | 0.001743 | 18.5753 |
| *B. linearis*  North | *B. macraei*  North | *B. linearis*  South | *B. macraei*  South | 0.032385 | 0.001583 | 20.4608 |
| *B. linearis*  South | *B. macraei*  North | *B. linearis*  Quintay | *B. macraei* Quintay | 0.032547 | 0.001854 | 17.554 |
| *B. linearis*  North | *B. vernalis*  North | *B. linearis*  South | *B. vernalis*  South | 0.033388 | 0.001474 | 22.655 |
| *B. linearis*  North | *B. vernalis*  North | *B. linearis*  South | *B. vernalis*  Hualpén | 0.033611 | 0.001562 | 21.5165 |
| *B. linearis*  North | *B. vernalis*  South | *B. linearis*  Quintay | *B. vernalis*  North | 0.033733 | 0.001562 | 21.6008 |
| *B. linearis*  South | *B. vernalis*  South | *B. linearis*  Quintay | *B. vernalis*  North | 0.033766 | 0.001523 | 22.164 |
| *B. linearis*  North | *B. vernalis*  North | *B. linearis*  Quintay | *B. vernalis*  South | 0.033877 | 0.001575 | 21.5081 |
| *B. linearis*  North | *B. vernalis*  South | *B. linearis*  South | *B. vernalis*  North | 0.033913 | 0.001412 | 24.0221 |
| *B. linearis*  North | *B. vernalis*  Hualpén | *B. linearis*  Quintay | *B. vernalis*  North | 0.033934 | 0.001694 | 20.0273 |
| *B. linearis*  South | *B. vernalis*  Hualpén | *B. linearis*  Quintay | *B. vernalis*  North | 0.033967 | 0.001658 | 20.4863 |
| *B. linearis*  North | *B. vernalis*  North | *B. linearis*  Quintay | *B. vernalis*  Hualpén | 0.0341 | 0.001667 | 20.4596 |
| *B. linearis*  North | *B. vernalis*  Hualpén | *B. linearis*  South | *B. vernalis*  North | 0.034226 | 0.001509 | 22.6866 |
| *B. linearis*  South | *B. vernalis*  North | *B. linearis*  Quintay | *B. vernalis*  South | 0.034436 | 0.001478 | 23.3019 |
| *B. linearis*  South | *B. vernalis*  North | *B. linearis*  Quintay | *B. vernalis*  Hualpén | 0.034748 | 0.001585 | 21.9241 |
| *B. linearis*  North | *B. vernalis*  Hualpén | *B. linearis*  Quintay | *B. vernalis*  South | 0.041569 | 0.001875 | 22.1727 |
| *B. linearis*  North | *B. vernalis*  South | *B. linearis*  Quintay | *B. vernalis*  Hualpén | 0.041592 | 0.001865 | 22.3061 |
| *B. linearis*  North | *B. vernalis*  South | *B. linearis*  South | *B. vernalis*  Hualpén | 0.041772 | 0.001758 | 23.7587 |
| *B. linearis*  North | *B. vernalis*  Hualpén | *B. linearis*  South | *B. vernalis*  South | 0.041861 | 0.001763 | 23.75 |
| *B. linearis*  South | *B. vernalis*  Hualpén | *B. linearis*  Quintay | *B. vernalis*  South | 0.042128 | 0.001833 | 22.9781 |
| *B. linearis*  South | *B. vernalis*  South | *B. linearis*  Quintay | *B. vernalis*  Hualpén | 0.042239 | 0.001836 | 23.0024 |

**Appendix S8 – f–Statistics**

**Table S1:** Estimation of f3-ratios for all possible topologies of four Baccharis species in Chile. The data set was divided into 7 blocks of 500 bp each. f3-statistics were calculated with Treemix v. 1.13. Taxa were divided into subgroups (North, South Quintay, Hualpén) according to their geography.

| **Target** | **Source 1** | **Source 2** | **f3 - ratio** | **standard error** | **z-score** |
| --- | --- | --- | --- | --- | --- |
| *B. × intermedia* South | *B. linearis*  North | *B. macraei*  Quintay | -0.00168 | 0.000546 | -3.0826 |
| *B. × intermedia* Quintay | *B. linearis*  North | *B. macraei*  North | -0.00204 | 0.000646 | -3.15757 |
| *B. × intermedia* South | *B. linearis*  Quintay | *B. macraei*  Quintay | -0.00241 | 0.000581 | -4.14761 |
| *B. × intermedia* South | *B. linearis*  Quintay | *B. macraei*  North | -0.00242 | 0.000662 | -3.66148 |
| *B. × intermedia* South | *B. linearis*  South | *B. vernalis*  South | -0.00251 | 0.000827 | -3.03534 |
| *B. × intermedia* South | *B. linearis*  South | *B. vernalis*  Hualpén | -0.00295 | 0.0009 | -3.27578 |
| *B. × intermedia* Quintay | *B. linearis*  South | *B. macraei*  South | -0.00335 | 0.000656 | -5.10684 |
| B. × intermedia North | *B. linearis*  North | *B. macraei*  North | -0.00337 | 0.000914 | -3.69343 |
| *B. × intermedia* South | *B. linearis*  South | *B. macraei*  North | -0.00339 | 0.000673 | -5.0445 |
| B. × intermedia North | *B. linearis*  Quintay | *B. macraei*  North | -0.00342 | 0.000973 | -3.51516 |
| *B. × intermedia* Quintay | *B. linearis*  Quintay | *B. macraei*  South | -0.00347 | 0.000761 | -4.55843 |
| *B. × intermedia* South | *B. linearis*  South | *B. macraei*  Quintay | -0.00355 | 0.000667 | -5.31757 |
| *B. × intermedia* Quintay | *B. linearis*  Quintay | *B. macraei*  North | -0.00371 | 0.000815 | -4.55128 |
| *B. × intermedia* Quintay | *B. linearis*  North | *B. macraei*  Quintay | -0.00375 | 0.000645 | -5.81271 |
| *B. × intermedia* Quintay | *B. linearis*  North | *B. macraei*  South | -0.00376 | 0.000672 | -5.59699 |
| B. × intermedia North | *B. linearis*  South | *B. macraei*  North | -0.00389 | 0.000995 | -3.91065 |
| *B. × intermedia* Quintay | *B. linearis*  South | *B. macraei*  North | -0.00395 | 0.000778 | -5.0752 |
| *B. × intermedia* Quintay | *B. linearis*  Quintay | *B. macraei*  Quintay | -0.00408 | 0.000686 | -5.95527 |
| *B. × intermedia* Quintay | *B. linearis*  South | *B. macraei*  Quintay | -0.00449 | 0.000694 | -6.46001 |
| *B. × intermedia* South | *B. linearis*  North | *B. macraei*  South | -0.00503 | 0.000587 | -8.57082 |
| *B. × intermedia* South | *B. linearis*  Quintay | *B. macraei*  South | -0.00513 | 0.00067 | -7.65881 |
| *B. × intermedia* South | *B. linearis*  South | *B. macraei*  South | -0.00575 | 0.000632 | -9.09752 |

**Table S2:** Estimation of f3-ratios for all possible topologies of four *Baccharis* species in Chile. The data set was divided into 7 blocks of 500 bp each. f3-statistics were calculated with Treemix v. 1.13. Taxa were divided into subgroups (North, South Quintay, Hualpén) according to their geography.

| **Target** | **Source 1** | **Source 2** | **f3 - ratio** | **standard error** | **z-score** |
| --- | --- | --- | --- | --- | --- |
| *B. × intermedia* South | *B. linearis*  North | *B. macraei*  Quintay | -0.00168 | 0.000546 | -3.0826 |
| *B. × intermedia* Quintay | *B. linearis*  North | *B. macraei*  North | -0.00204 | 0.000646 | -3.15757 |
| *B. × intermedia* South | *B. linearis*  Quintay | *B. macraei*  Quintay | -0.00241 | 0.000581 | -4.14761 |
| *B. × intermedia* South | *B. linearis*  Quintay | *B. macraei*  North | -0.00242 | 0.000662 | -3.66148 |
| *B. × intermedia* South | *B. linearis*  South | *B. vernalis*  South | -0.00251 | 0.000827 | -3.03534 |
| *B. × intermedia* South | *B. linearis*  South | *B. vernalis*  Hualpén | -0.00295 | 0.0009 | -3.27578 |
| *B. × intermedia* Quintay | *B. linearis*  South | *B. macraei*  South | -0.00335 | 0.000656 | -5.10684 |
| B. × intermedia North | *B. linearis*  North | *B. macraei*  North | -0.00337 | 0.000914 | -3.69343 |
| *B. × intermedia* South | *B. linearis*  South | *B. macraei*  North | -0.00339 | 0.000673 | -5.0445 |
| B. × intermedia North | *B. linearis*  Quintay | *B. macraei*  North | -0.00342 | 0.000973 | -3.51516 |
| *B. × intermedia* Quintay | *B. linearis*  Quintay | *B. macraei*  South | -0.00347 | 0.000761 | -4.55843 |
| *B. × intermedia* South | *B. linearis*  South | *B. macraei*  Quintay | -0.00355 | 0.000667 | -5.31757 |
| *B. × intermedia* Quintay | *B. linearis*  Quintay | *B. macraei*  North | -0.00371 | 0.000815 | -4.55128 |
| *B. × intermedia* Quintay | *B. linearis*  North | *B. macraei*  Quintay | -0.00375 | 0.000645 | -5.81271 |
| *B. × intermedia* Quintay | *B. linearis*  North | *B. macraei*  South | -0.00376 | 0.000672 | -5.59699 |
| B. × intermedia North | *B. linearis*  South | *B. macraei*  North | -0.00389 | 0.000995 | -3.91065 |
| *B. × intermedia* Quintay | *B. linearis*  South | *B. macraei*  North | -0.00395 | 0.000778 | -5.0752 |
| *B. × intermedia* Quintay | *B. linearis*  Quintay | *B. macraei*  Quintay | -0.00408 | 0.000686 | -5.95527 |
| *B. × intermedia* Quintay | *B. linearis*  South | *B. macraei*  Quintay | -0.00449 | 0.000694 | -6.46001 |
| *B. × intermedia* South | *B. linearis*  North | *B. macraei*  South | -0.00503 | 0.000587 | -8.57082 |
| *B. × intermedia* South | *B. linearis*  Quintay | *B. macraei*  South | -0.00513 | 0.00067 | -7.65881 |
| *B. × intermedia* South | *B. linearis*  South | *B. macraei*  South | -0.00575 | 0.000632 | -9.09752 |

**Table S3:** Estimation of f3-ratios for all possible topologies of four *Baccharis* species in Chile. The data set was divided into 7 blocks of 500 bp each. f3-statistics were calculated with Treemix v. 1.13. Taxa were divided into subgroups (North, South Quintay, Hualpén) according to their geography (continued).

| **Population 1** | **Population 2** | **Population 3** | **Population 4** | **f4 value** | **standard error** | **Z-score** |
| --- | --- | --- | --- | --- | --- | --- |
| *B. linearis*  North | *B. macraei*  North | *B. linearis*  South | *B. macraei* Quintay | 0.032323 | 0.001683 | 19.2065 |
| *B. linearis*  South | *B. macraei* Quintay | *B. linearis*  Quintay | *B. macraei*  North | 0.032382 | 0.001743 | 18.5753 |
| *B. linearis*  North | *B. macraei*  North | *B. linearis*  South | *B. macraei*  South | 0.032385 | 0.001583 | 20.4608 |
| *B. linearis*  South | *B. macraei*  North | *B. linearis*  Quintay | *B. macraei* Quintay | 0.032547 | 0.001854 | 17.554 |
| *B. linearis*  North | *B. vernalis*  North | *B. linearis*  South | *B. vernalis*  South | 0.033388 | 0.001474 | 22.655 |
| *B. linearis*  North | *B. vernalis*  North | *B. linearis*  South | *B. vernalis*  Hualpén | 0.033611 | 0.001562 | 21.5165 |
| *B. linearis*  North | *B. vernalis*  South | *B. linearis*  Quintay | *B. vernalis*  North | 0.033733 | 0.001562 | 21.6008 |
| *B. linearis*  South | *B. vernalis*  South | *B. linearis*  Quintay | *B. vernalis*  North | 0.033766 | 0.001523 | 22.164 |
| *B. linearis*  North | *B. vernalis*  North | *B. linearis*  Quintay | *B. vernalis*  South | 0.033877 | 0.001575 | 21.5081 |
| *B. linearis*  North | *B. vernalis*  South | *B. linearis*  South | *B. vernalis*  North | 0.033913 | 0.001412 | 24.0221 |
| *B. linearis*  North | *B. vernalis*  Hualpén | *B. linearis*  Quintay | *B. vernalis*  North | 0.033934 | 0.001694 | 20.0273 |
| *B. linearis*  South | *B. vernalis*  Hualpén | *B. linearis*  Quintay | *B. vernalis*  North | 0.033967 | 0.001658 | 20.4863 |
| *B. linearis*  North | *B. vernalis*  North | *B. linearis*  Quintay | *B. vernalis*  Hualpén | 0.0341 | 0.001667 | 20.4596 |
| *B. linearis*  North | *B. vernalis*  Hualpén | *B. linearis*  South | *B. vernalis*  North | 0.034226 | 0.001509 | 22.6866 |
| *B. linearis*  South | *B. vernalis*  North | *B. linearis*  Quintay | *B. vernalis*  South | 0.034436 | 0.001478 | 23.3019 |
| *B. linearis*  South | *B. vernalis*  North | *B. linearis*  Quintay | *B. vernalis*  Hualpén | 0.034748 | 0.001585 | 21.9241 |
| *B. linearis*  North | *B. vernalis*  Hualpén | *B. linearis*  Quintay | *B. vernalis*  South | 0.041569 | 0.001875 | 22.1727 |
| *B. linearis*  North | *B. vernalis*  South | *B. linearis*  Quintay | *B. vernalis*  Hualpén | 0.041592 | 0.001865 | 22.3061 |
| *B. linearis*  North | *B. vernalis*  South | *B. linearis*  South | *B. vernalis*  Hualpén | 0.041772 | 0.001758 | 23.7587 |
| *B. linearis*  North | *B. vernalis*  Hualpén | *B. linearis*  South | *B. vernalis*  South | 0.041861 | 0.001763 | 23.75 |
| *B. linearis*  South | *B. vernalis*  Hualpén | *B. linearis*  Quintay | *B. vernalis*  South | 0.042128 | 0.001833 | 22.9781 |
| *B. linearis*  South | *B. vernalis*  South | *B. linearis*  Quintay | *B. vernalis*  Hualpén | 0.042239 | 0.001836 | 23.0024 |

**Appendix S9 – Treemix analysis**

**Figure S1:** ln(likelihood) in comparison of the number of migration events for every population of every taxon analysed in a "Treemix" analysis.

**_
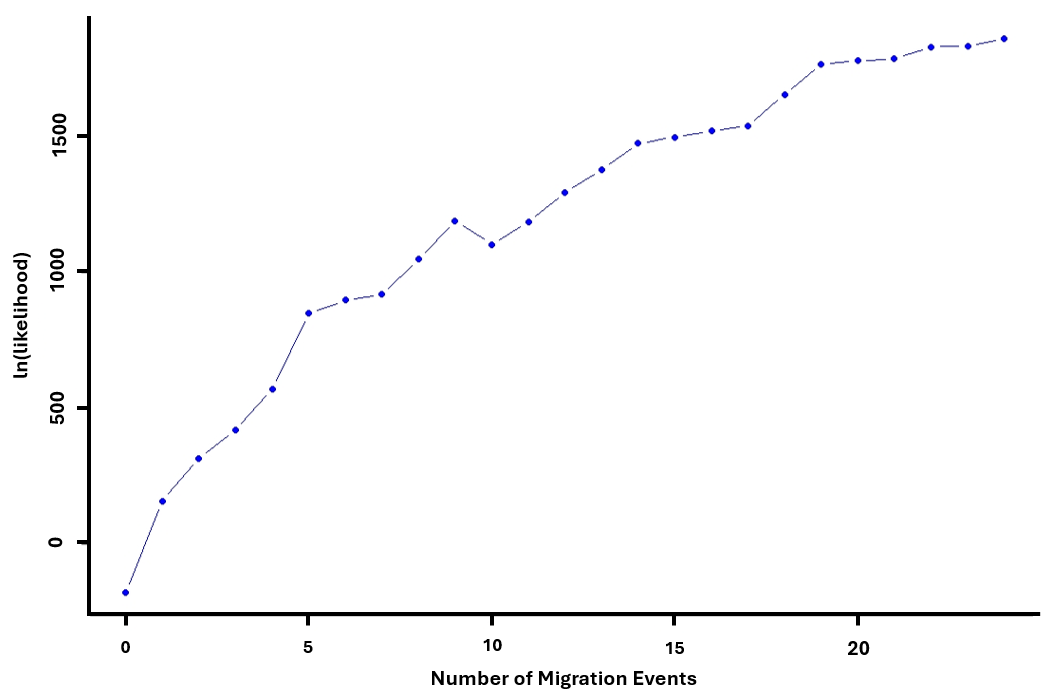
_**

**Figure S2:** ln(likelihood) in comparison of the number of migration events resulting from the "Treemix" analysis for 12 populations. This model used in the study.


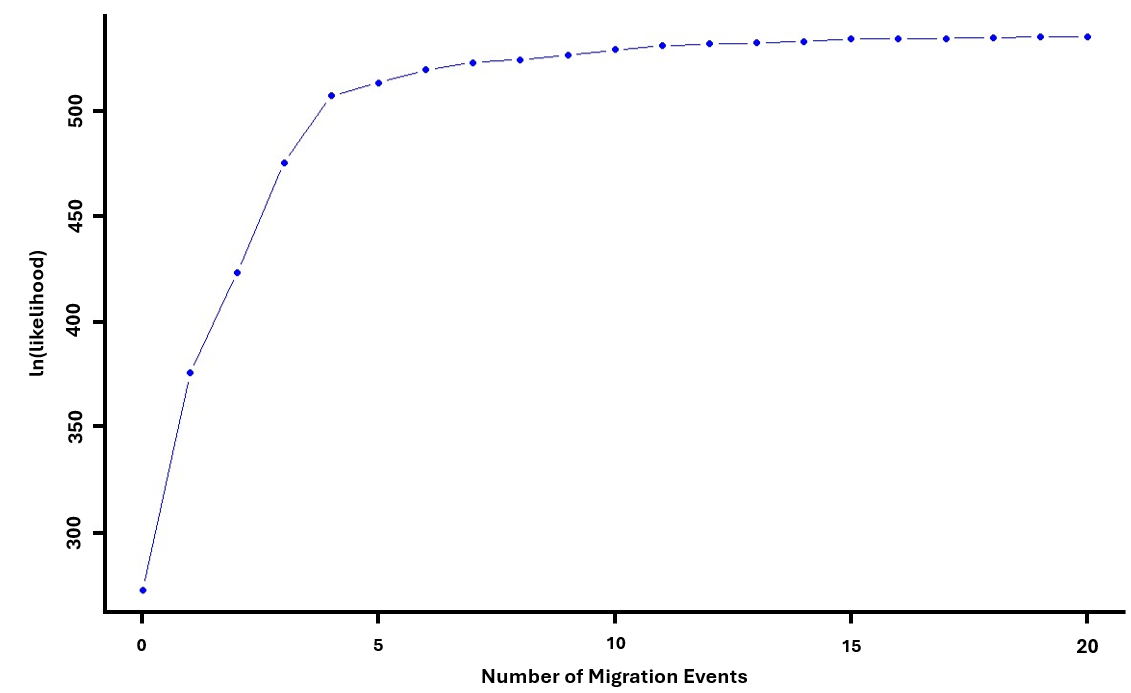


**Figure S3:** Residual fit between the different groups of Baccharis. Taxa were divided into subgroups (North, South Quintay, Hualpén) according to their geography (continued).


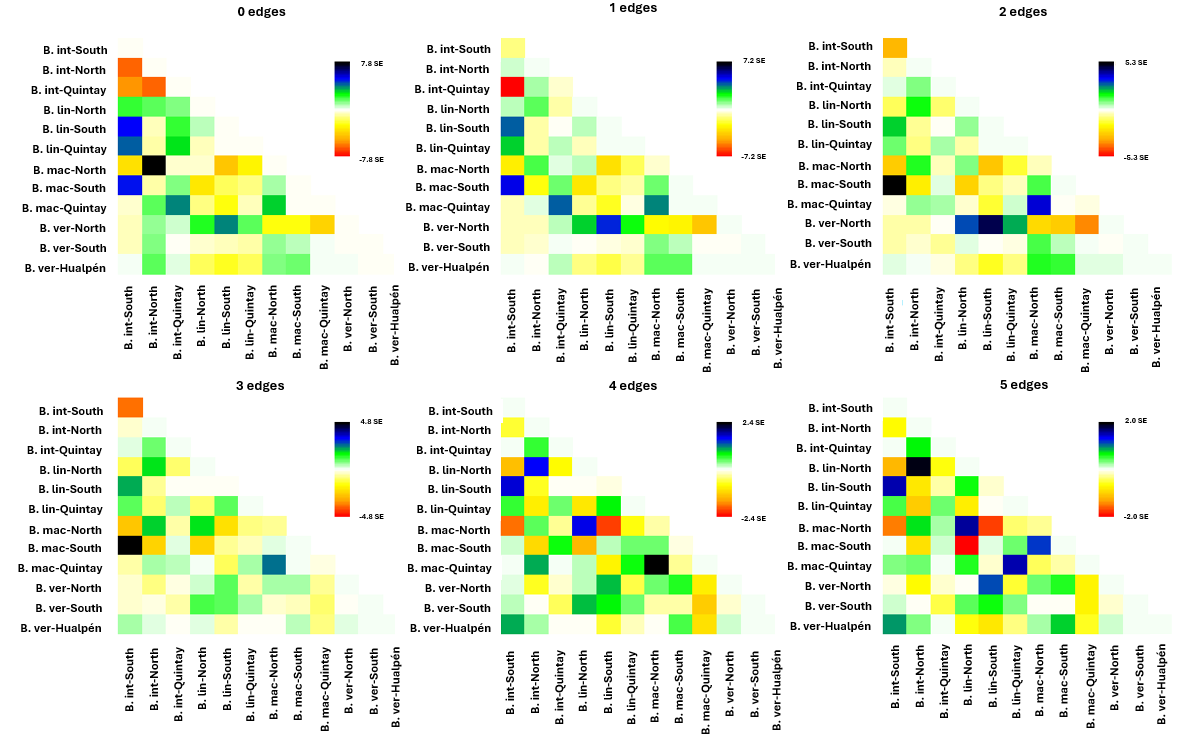


**Figure S4:** Residual fit between the different groups of Baccharis. Taxa were divided into subgroups (North, South Quintay, Hualpén) according to their geography (continued).


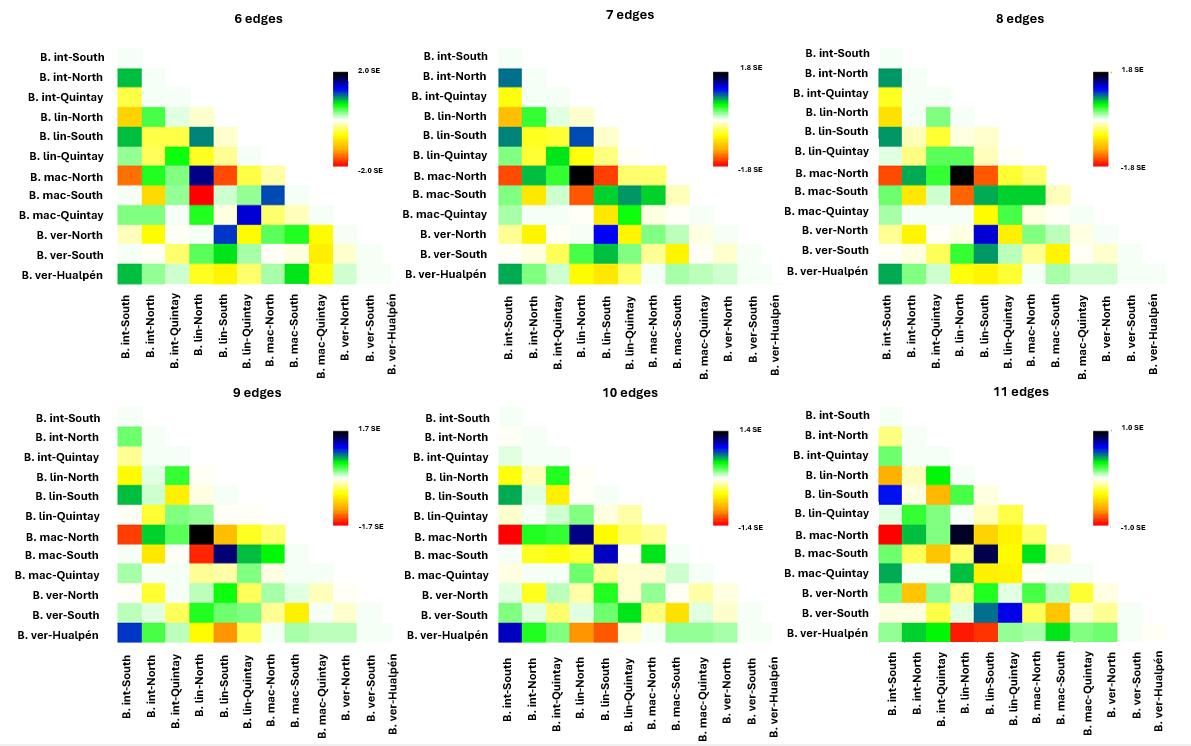


**Figure S5:** Maximum likelihood trees including different number of migration events (edges) between taxa of Baccharis. The arrow is coloured by migration weight, and branch lengths are proportional to genetic drift. Taxa were divided into subgroups (North, South Quintay, Hualpén) according to their geography.


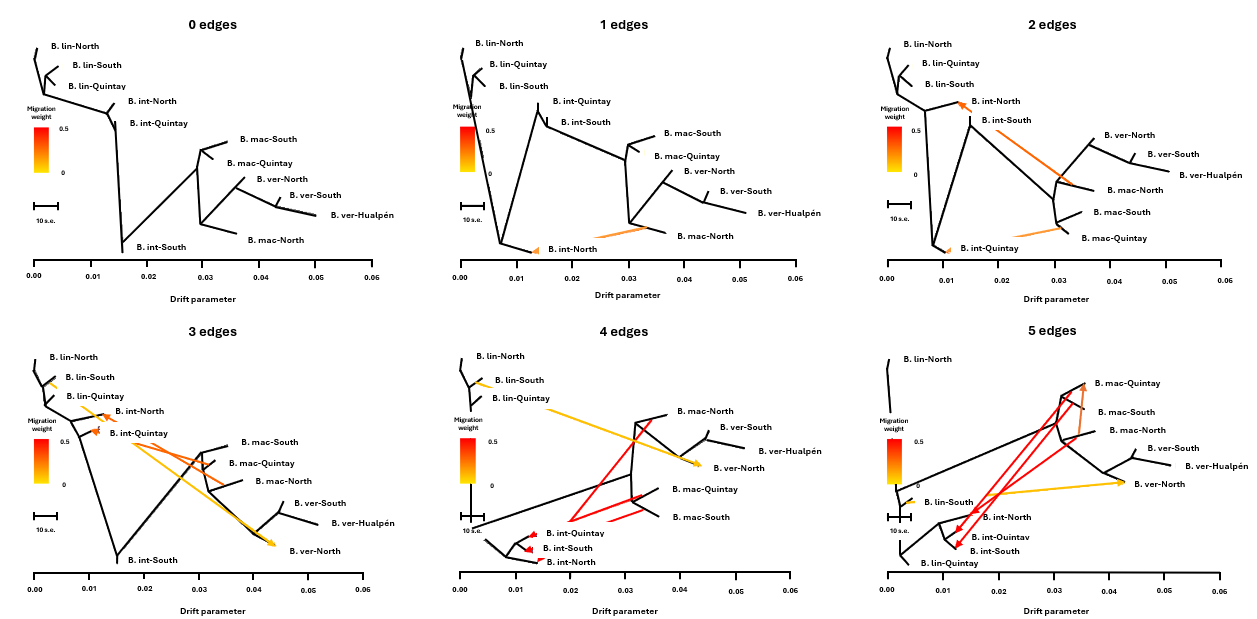


**Figure S6:** Maximum likelihood trees including different number of migration events (edges) between taxa of *Baccharis*. The arrow is coloured by migration weight, and branch lengths are proportional to genetic drift. Taxa were divided into subgroups (North, South Quintay, Hualpén) according to their geography (continued).


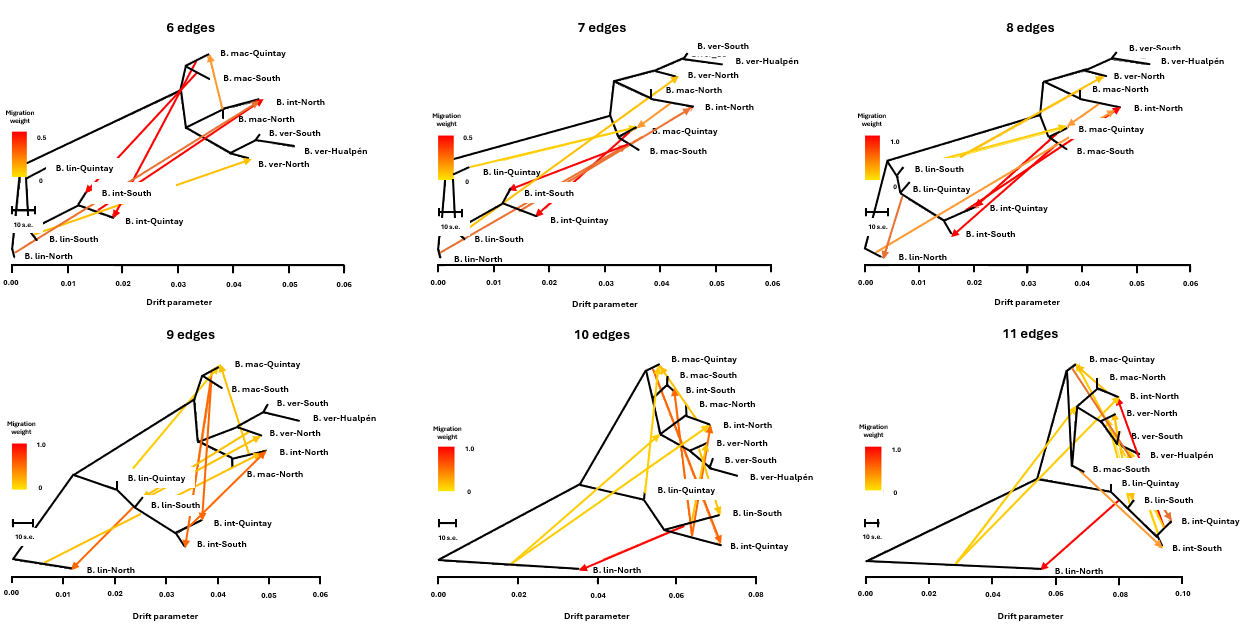

Supplement: Supplementary file 1 — Data S1: ece372249‐sup‐0001‐DataS1.docx. [file ECE3-15-e72249-s001.docx]
